# Health Effects of Increasing Protein Intake Above the Current Population Reference Intake in Older Adults: A Systematic Review of the Health Council of the Netherlands

**DOI:** 10.1093/advances/nmab140

**Published:** 2021-11-23

**Authors:** Linda M Hengeveld, Janette de Goede, Lydia A Afman, Stephan J L Bakker, Joline W J Beulens, Ellen E Blaak, Eric Boersma, Johanna M Geleijnse, Johannes (Hans) B van Goudoever, Maria T E Hopman, Jolein A Iestra, Stef P J Kremers, Ronald P Mensink, Nicole M de Roos, Coen D A Stehouwer, Janneke Verkaik-Kloosterman, Emely de Vet, Marjolein Visser

**Affiliations:** Health Council of the Netherlands, The Hague, The Netherlands; Health Council of the Netherlands, The Hague, The Netherlands; Division of Human Nutrition and Health, Wageningen University and Research, Wageningen, The Netherlands; Department of Internal Medicine, University Medical Center Groningen, University of Groningen, Groningen, The Netherlands; Department of Epidemiology and Data Science, Amsterdam Public Health Research Institute, Amsterdam, The Netherlands; Amsterdam Cardiovascular Sciences Research Institute, Amsterdam University Medical Center, location VUmc, Amsterdam, The Netherlands; Julius Centre for Health Sciences and Primary Care, University Medical Centre Utrecht, Utrecht, The Netherlands; Department of Human Biology, NUTRIM School for Nutrition and Translational Research in Metabolism, Maastricht University, Maastricht, The Netherlands; Erasmus MC, University Medical Center, Department of Cardiology, Rotterdam, The Netherlands; Health Council of the Netherlands, The Hague, The Netherlands; Division of Human Nutrition and Health, Wageningen University and Research, Wageningen, The Netherlands; Amsterdam UMC, University of Amsterdam, Vrije Universiteit, Emma Children's Hospital, Department of Pediatrics, Amsterdam, The Netherlands; Department of Physiology, Radboud Institute for Health Sciences, Radboud University Medical Center, Nijmegen, The Netherlands; Julius Centre for Health Sciences and Primary Care, University Medical Centre Utrecht, Utrecht, The Netherlands; Department of Health Promotion, NUTRIM School of Nutrition and Translational Research in Metabolism, Maastricht University Medical Centre+, Maastricht, The Netherlands; Department of Nutrition and Movement Sciences, NUTRIM School of Nutrition and Translational Research in Metabolism, Maastricht University Medical Center, Maastricht, The Netherlands; Division of Human Nutrition and Health, Wageningen University and Research, Wageningen, The Netherlands; CARIM School for Cardiovascular Diseases, Maastricht University, Maastricht, the Netherlands; Department of Internal Medicine, Maastricht University Medical Centre+, Maastricht, The Netherlands; National Institute of Public Health and the Environment, Bilthoven, The Netherlands; Department of Social Sciences, Chair group Consumption and Healthy Lifestyles, Wageningen University and Research, Wageningen, The Netherlands; Department of Health Sciences, Faculty of Science, Amsterdam Public Health Research Institute, Vrije Universiteit Amsterdam, Amsterdam, The Netherlands

**Keywords:** dietary protein, amino acids, protein supplements, aging, resistant exercise, muscle mass, physical function, intervention studies, systematic literature review, dietary reference value

## Abstract

Whether older adults need more protein than younger adults is debated. The population reference intake for adults set by the European Food Safety Authority is 0.83 g/kg body weight (BW)/d based primarily on nitrogen balance studies, but the underlying data on health outcomes are outdated. An expert committee of the Health Council of the Netherlands conducted a systematic review (SR) of randomized controlled trials (RCTs) examining the effect of increased protein intake on health outcomes in older adults from the general population with an average habitual protein intake ≥0.8 g/(kg BW · d). Exposures were the following: *1*) extra protein compared with no protein and *2*) extra protein and physical exercise compared with physical exercise. Outcomes included lean body mass, muscle strength, physical performance, bone health, blood pressure, serum glucose and insulin, serum lipids, kidney function, and cognition. Data of >1300 subjects from 18 RCTs were used. Risk of bias was judged as high (*n* = 9) or “some concerns” (*n* = 9). In 7 of 18 RCTs, increased protein intake beneficially affected ≥1 of the tested outcome measures of lean body mass. For muscle strength, this applied to 3 of 8 RCTs in the context of physical exercise and in 1 of 7 RCTs without physical exercise. For the other outcomes, <30% (0–29%) of RCTs showed a statistically significant effect. The committee concluded that increased protein intake has a *possible beneficial effect* on lean body mass and, when combined with physical exercise, muscle strength; *likely no effect* on muscle strength when not combined with physical exercise, or on physical performance and bone health; an *ambiguous effect* on serum lipids; and that *too few RCTs were available* to allow for conclusions on the other outcomes. This SR provides insufficiently convincing data that increasing protein in older adults with a protein intake ≥0.8 g/(kg BW · d) elicits health benefits.

## Teaser

**Statement of Significance**: This systematic review provides a comprehensive, transparent, and up-to-date overview of randomized controlled trials that explicitly focus on potential health effects of protein intakes above the nitrogen-balance studies-based population reference intake of 0.8 g/(kg BW · d) in older adults from the general population. This review also serves as an additional source of evidence for deriving a recommended protein intake for older adults.

## Introduction

Dietary proteins are essential for healthy structure and functioning of the human body. The population reference intake (PRI) of protein for (healthy) adults was set at 0.83 g/[kg body weight (BW) ⋅ d] by the WHO in 2007 (1), and a similar PRI was set by the European Food Safety Authority (EFSA) in 2012 (2). This PRI applies to all healthy adults, regardless of age. However, several international groups of scientific experts advocate a higher PRI for older adults than for younger adults, because older adults need larger amounts of protein to optimally preserve muscle mass and function (3–5). Some countries have already set higher dietary reference values (DRVs) for older adults; for example, the Nordic countries in 2012 (6) derived a PRI of 1.1 to 1.3 g/(kg BW ⋅ d) and the German-speaking DACH countries in 2017 (7, 8) derived a PRI of 1.0 g/(kg BW · d) for older adults (age ≥65 y). The Health Council of the Netherlands, commissioned by the Ministry of Health, Welfare, and Sport, periodically evaluates its DRVs for energy, macronutrients, and micronutrients, and recently revised its latest published DRVs for protein from 2001 (9). Because of the ongoing scientific debate about the optimal protein intake for older adults, the evaluation specifically focused on the DRVs for older adults (age ≥60 y).

From the perspective of harmonization of DRVs across the European Union, the Permanent Committee on Nutrition, set by the council, used the EFSA scientific report on protein (2) as a starting point, and evaluated whether it agreed with EFSA's scientific basis and methodology. EFSA, in accordance with the WHO, based its DRVs for protein primarily on a meta-analysis (MA) of nitrogen-balance studies in healthy adults performed by Rand et al. (10) and derived an average protein requirement of 0.66 g/(kg BW ⋅ d) and a PRI of 0.83 g/(kg BW ⋅ d). EFSA also evaluated human observational and intervention studies on health outcomes but concluded that the available data on the effects of protein intake on muscle mass, muscle function, BW control, obesity risk, insulin sensitivity, glucose homeostasis, and bone health could not be used for setting DRVs for protein. The Dutch committee agreed with the approach of EFSA and the conclusions drawn based on the evidence available at that time. However, the committee judged that the literature needed to be updated because many new publications on this topic had been published since the release of the EFSA report in 2012.

The committee searched for recent systematic reviews (SRs) and systematic and transparent reports (i.e., those with a clear description and argumentation of the followed methodology, including weighing of the evidence) on protein intake in relation to health outcomes in older adults, but judged that those available were limited with regard to the degree of detail of the included individual studies required for deriving DRVs. Most importantly, the majority of SRs and reports did not provide information about the total (habitual) protein intake [in g/(kg BW ⋅ d)] of the participants. In addition, various types of exposure were often mixed; for example, protein alone compared with protein combined with physical exercise. Those exposures might differentially affect health outcomes (11) and should therefore be separately investigated. Furthermore, several SRs and reports included cross-sectional studies, which have a high risk of recall bias and provide no evidence for a *temporal* relation (12). The committee judged that none of those SRs or reports provided the information needed to derive DRVs for protein or that they did not include the most recent literature. Moreover, none of the SRs specifically addressed the question of whether increasing protein intake in older adults who meet the current PRI of 0.83 g protein/kg BW/d would yield health benefits. Therefore, the committee performed an SR with the aim of determining whether a protein intake higher than the PRI of 0.83 g/(kg BW ⋅ d) derived from nitrogen-balance data affects health outcomes in older adults. The SR was focused on randomized controlled trials (RCTs) among older adults in the general population with an average habitual protein intake of ≥0.8 g/(kg BW ⋅ d). This SR served as ancillary evidence for the revised DRVs of protein for older adults in the Netherlands.

## Methods

The present SR was conducted by the multidisciplinary Permanent Committee on Nutrition, comprising experts in the research fields of nutrition, health, physiology, epidemiology, and statistics. They filled out declarations of interest, which were published (in Dutch) on the website of the Health Council of the Netherlands (www.gezondheidsraad.nl). The committee performed an SR of peer-reviewed RCTs on the effects of increased protein intake on 9 health outcomes in older adults from the general population. The committee had regular meetings to determine the scope and protocol of the SR, discuss the eligibility and content of the scientific literature, and grade the evidence in order to draw final conclusions.

### Literature search

The committee initially aimed to base its evaluation on SRs of RCTs and prospective cohort studies. Therefore, a systematic literature search was performed to identify relevant SRs, including MAs and individual participant data analyses, on the relation between protein intake and health outcomes in older adults. PubMed was searched on 23 April 2020 for English language publications with no date limit set. The search strategy (**Supplemental Methods 1**) was developed with help from an experienced information specialist. Also, the information specialist conducted the search and removed duplicates. Titles and abstracts of all retrieved references were screened by 1 author (LMH). This author also assessed the full texts of potentially relevant publications based on the predefined inclusion and exclusion criteria detailed below. Any uncertainties regarding the eligibility of a publication were discussed with a second author (JdG) or with the full committee and resolved through consensus. A second author performed an additional literature search for SRs in Scopus, but this yielded no additional relevant SRs.

The committee concluded that none of the retrieved SRs as such was appropriate for the goal of deriving DRVs for protein, for the reasons previously outlined. It therefore decided to base its evaluation on individual studies and used the retrieved SRs to identify those studies. These SRs should cover the totality of the evidence (including the oldest studies) since the majority of SRs searched the literature from database inception (no date limit). To identify recent individual RCTs that had not yet been included in an SR, a second systematic literature search was performed in PubMed and Scopus (Supplemental Methods 1). This search for English language publications was limited to studies published in 2018, 2019, and 2020 (up until 23 April 2020) as this would cover the studies published after the inclusion date of the most recent SRs. Reference lists from eligible publications were hand-searched for relevant studies not found in the database search. Last, to ensure that no relevant publications had been missed, the committee—which included researchers in the field of protein and aging—was asked to report any additional publications that were considered relevant for this advisory report.

### Study selection: inclusion and exclusion criteria

Nine health outcomes were selected for evaluation (based on availability in the literature): lean body mass, muscle strength, physical performance, bone health, blood pressure, serum glucose and insulin, serum lipids, kidney function, and cognition.

The committee included only RCTs, for the following reasons: *1*) RCTs can provide more robust evidence for a *causal* relation than can be provided by prospective cohort studies; and *2*) in the majority of prospective cohort studies available, categories of total protein intake specifically informative to the PRI were not used. For example, these studies often did not include a protein category at the level of the current PRI [0.8 g/(kg BW ⋅ d)], did not use this category as reference, or did not report protein intake in (or in a way that could be recalculated to) g/(kg BW ⋅ d). Because of this, it would be very difficult to specify if any additional protein intake beyond the PRI of 0.83 g/(kg BW ⋅ d) would elicit health benefits (or harm), and if so, what the exact optimal amount of protein would be. All RCTs thus retrieved were further assessed for eligibility by using the prespecified inclusion and exclusion criteria (**Supplemental Methods 2**). In short, the committee included RCTs with a minimum duration of 4 wk that were performed among older adults with a minimal sampling age of 50 y (or—when sampling age was not reported in the study—an average age of ≥65 y), with an average habitual protein intake of ≥0.8 g/(kg BW ⋅ d) and who were living at home (independently), in a care home, or in a nursing home. Studies in which the study population consisted solely of hospitalized or immobilized patients or of individuals with a specific disease, such as chronic heart failure or chronic obstructive pulmonary disease, were excluded. The committee included studies in which the participants were exposed to protein or a mix of (≥4) amino acids, such as protein supplements, amino acid supplements, and protein-rich or protein-enriched foods. The committee excluded studies that were not isocaloric, in which the intervention groups and control groups differed (intentionally) in more ways than protein exposure alone and those that were performed in the context of a weight loss program.

### Data extraction

The following study data were extracted by 1 author (LMH): first author, publication year, country, study population [i.e., age, sex, health characteristics, BMI (in kg/m^2^), race], sample size, (type of) protein intervention and control intervention, total protein intake, habitual protein intake, protein dose, concomitant physical exercise, dietary compliance, study duration, (precalculated) statistical power, funding source, specific outcome measure(s) examined, and results.

#### Total protein intake, habitual protein intake, and protein dose

Since the underlying aim of the present SR was to derive a DRV for protein, the committee was particularly interested in the factual total protein intake [preferentially expressed in g/(kg BW ⋅ d)] of the study population, rather than the supplemented or prescribed amount of protein only. Total protein intake is the sum of the habitual protein intake and the supplemented or prescribed protein dose. Habitual protein intake is the amount of protein that a person usually consumes on an average day outside the trial context and generally is the baseline protein intake during the trial. Protein dose was defined as the difference in achieved total protein intake (i.e., habitual protein intake plus factually consumed amount of supplemented or prescribed protein), between the intervention group and the control group during follow-up. When not specifically reported in the article, the committee assumed that the total protein intake of the control group was similar to the habitual protein intake, since the control group is generally not provided or prescribed additional protein during the trial. Because the effect of extra protein intake might depend on the habitual protein intake, the RCTs were grouped according to the following 4 domains of habitual protein intake (only if sufficient data were available): ≥0.8 to <0.9, ≥0.9 to <1.0, ≥1.0 to <1.1, or ≥1.1 g/(kg BW ⋅ d). Cutoffs were based on currently used PRIs for protein (1, 2, 6–8, 13) and protein recommendations from expert groups (3, 4). Studies in which the habitual protein intake was <0.8 g/(kg BW ⋅ d) were not included, because the committee aimed to determine health effects of protein intake above the PRI derived from nitrogen-balance studies.

#### Concomitant physical exercise

The 2017 Dutch Physical Activity Guidelines (14) recommend that adults, including older adults, perform muscle- and bone-strengthening activities at ≥2 times/wk. Increasing protein intake in the context of physical exercise is suggested to have an additive or synergistic effect on muscle mass and muscle strength in younger and older adults compared with protein intake alone (11). This finding implies that protein alone might exert health effects different from those exerted by protein in the context of physical exercise. Therefore, the committee defined the following 2 study categories: *1*) studies examining the effect of protein intake only (without a physical exercise intervention in both intervention group and control group); and *2*) studies examining the effect of protein intake in the context of physical exercise (both intervention group and control group received a physical exercise intervention). Thus, studies were excluded if protein intake was not the only contrast between the intervention group and the control group.

### Study quality

The risk of bias of the included studies was assessed using the revised Cochrane risk of bias tool for randomized trials (RoB 2) (15). The RoB 2 tool addresses bias arising from the following sources: *1*) the randomization process, *2*) deviations from intended interventions, *3*) missing outcome data, *4*) measurement of the outcome, and *5*) selection of the reported result. The risk of bias in each domain was scored as “low risk of bias,” “some concerns,” or “high risk of bias.” Together, these risk scores resulted in an overall judgment of the risk of bias, also in terms of “low risk of bias,” “some concerns,” or “high risk of bias.” The assessment was performed by 1 author (LMH). Any uncertainties were discussed with a second author (JdG) or with the full committee.

### Data synthesis

#### Evaluation of the evidence

The committee evaluated the scientific evidence regarding the effect of increased protein intake on each of the 9 selected health outcomes in older adults. For each outcome, the totality of the evidence was considered, followed by subgroup analyses, sensitivity analyses, and other considerations. With regard to subgroup analyses, the committee evaluated whether effect modification by concomitant physical exercise or domain of habitual protein intake was present. If the committee's judgment of the totality of the evidence was that there was *likely no effect*, or if there were *too few studies* (see the next paragraph for the possible categories of conclusions drawn by the committee), no stratification was made. Sensitivity analyses were conducted to examine whether heterogeneity in results across studies could be explained by the following factors: type of protein intervention (categorized as protein or amino acid supplements, 1 or a few protein-(en)rich(ed) foods, or high-protein diets), risk of bias (categorized as low, some concerns, or high), and if the study was statistically powered for the given outcome measure or not. The committee also evaluated, as an exploratory analysis, whether there was an indication for a dose–response relation (**Supplemental Methods 3**). This relation could only be evaluated by comparing studies, and not by comparing individuals within studies; therefore, this evaluation was considered “exploratory.” Other considerations refer to limitations of the included RCTs regarding, for example, the validity of the outcome measurements or the sample size of the study.

#### Applying decision rules

Based on the overall effects observed, subgroup evaluations, sensitivity analyses, and other considerations, and by using predefined decision rules (**Table 1**), the committee judged the totality of the evidence for each health outcome. Six predefined categories of conclusions were distinguished: a *convincing (beneficial/unfavorable) effect*, a *likely (beneficial/unfavorable) effect*, a *possible (beneficial/unfavorable) effect*, an *ambiguous effect, likely no effect* or *too few studies*.

**TABLE 1:** Set of possible conclusions, and decision rules for drawing those conclusions for the effect of increased protein intake on health outcomes^1^

Conclusion	Decision rules
A convincing beneficial effect	If a total of ≥3 studies are available, ≥75% of which show a beneficial effect and none of which show an unfavorable effect
A likely beneficial effect	If a total of ≥3 studies are available, 50–74% of which show a beneficial effect and none of which show an unfavorable effect
A possible beneficial effect	If a total of ≥3 studies are available, 25–49% of which show a beneficial effect and none of which show an unfavorable effect
An ambiguous effect	If a total of ≥3 studies are available and studies show conflicting results; this involves a combination of both beneficial effects and unfavorable effects, without the overall picture clearly pointing in 1 direction
Likely no effect	If a total of ≥3 studies are available, <25% of which show a beneficial effect and none of which show an unfavorable effect
Too few studies	A total of <3 studies are available or <3 studies with sufficient statistical power are available

1 Wherever reference is made to beneficial effects or unfavorable effects, this concerns statistically significant beneficial or statistically significant unfavorable effects, respectively. All categories may include neutral studies, i.e., studies in which no statistically significant effect was found. Those rules also apply to an unfavorable effect. Unfavorable effects were not expected based on a first judgment of the literature, so in the interest of readability, those rules are not specified here.

Many RCTs assessed multiple specific outcome measures reflecting a similar health outcome, such as handgrip strength, knee extensor strength, and leg press as measures of muscle strength. Those specific outcome measures, also known as *contrasts*, are likely dependent on each other, which would artificially inflate the number of positive results. Therefore, conclusions were primarily based on the total number of RCTs (instead of contrasts), the percentage of RCTs showing an effect and the direction of the effect in any of the investigated outcome measures. Secondarily, the percentage of included RCTs with a statistically significant result was compared with the percentage of tested contrasts with a statistically significant result, across all RCTs with the given health outcome. If the percentage of contrasts with an effect differed substantially from the percentage of RCTs with an effect, this could lead to a modification (downgrading) of the conclusion. In this process, the committee adopted a liberal approach because it considered the RCTs that showed a beneficial effect for ≥1 of the tested contrasts as an RCT with an overall beneficial effect (even though, in some cases, several of the contrasts examined showed no effects).

## Results

### Study identification

**FIGURE 1:**
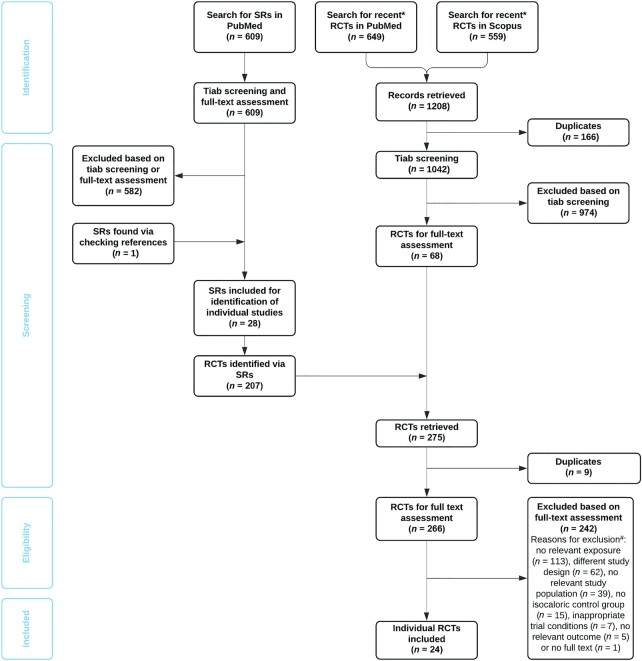
Flow chart of study selection. RCT, randomized controlled trial; SR, systematic review; tiab, title and abstract. *Published in 2018, 2019, or 2020 (up to 23 April 2020). ^#^No relevant exposure concerns, e.g., no protein or protein-based intervention, a cointervention (e.g., vitamin D) provided to the intervention group only or the type or distribution of protein rather than its amount was examined; different study design concerned, e.g., prospective cohort studies, no control group, or an intervention period <4 wk; no relevant study population concerns, e.g., people aged <50 y or hospitalized people; and inappropriate trial conditions concern, e.g., interventions conducted during a weight loss program.

The literature search for SRs in PubMed yielded 609 publications (**Figure 1**). After excluding publications based on title/abstract screening or full-text assessment, the committee selected 27 SRs for the evaluation. Checking reference lists yielded 1 additional SR. From those 28 SRs (16–43) that were used for identifying individual RCTs, 207 individual studies were retrieved. The additional search for recent individual studies published in 2018, 2019, or 2020 in PubMed (*n* = 649) and Scopus (*n* = 559) yielded, after removal of duplicates, 1042 unique publications. Based on title/abstract screening, 974 publications were excluded, leaving 68 publications for full-text assessment. Nine publications were found via SRs as well as the additional literature search and, thus, a total of 266 unique publications remained for full-text assessment. Publications that were unclear with respect to eligibility as well as publications that met the inclusion criteria according to the first assessor were then discussed with the full committee, which judged that a total of 24 publications were eligible for inclusion in the committee's evaluation (44–67). These 24 publications reported on 18 unique RCTs (**Table 2**). No additional studies were identified through consultation of the committee members.

**TABLE 2:** Overview of RCTs and the health outcomes they addressed^1^^,2^

Author(s) and publication year of RCT (reference no.)	Health outcomes addressed
Arnarson et al. 2013 (44), Ramel et al. (45)	Lean body mass, muscle strength, physical performance, kidney function
Bhasin et al. 2018 (46)	Lean body mass, muscle strength, physical performance, serum lipids, kidney function
Campbell et al. 1995 (47)	Lean body mass
Chalé et al. 2013 (48)	Lean body mass, muscle strength, physical performance
Dillon et al. 2009 (49)	Lean body mass, muscle strength
Fernandes et al. 2018 (50), Sugihara Junior et al. 2018 (51)	Lean body mass, muscle strength, bone health, serum glucose and insulin, serum lipids
Hodgson et al. 2012 (52), Zhu et al. 2011 (53), Zhu et al. 2015 (54)	Lean body mass, muscle strength, physical performance, bone health, blood pressure
Ispoglou et al. 2016 (55)	Lean body mass, muscle strength, physical performance, bone health
Kersetter et al. 2015 (56)	Lean body mass, bone health, kidney function
Mitchell et al. 2015 (57)	Lean body mass, muscle strength
Mitchell et al. 2017 (58)	Lean body mass, muscle strength, physical performance
Nabuco et al. 2018 (59), Nabuco et al. 2019a (60), Nabuco et al. 2019b (61)	Lean body mass, muscle strength, physical performance, blood pressure, serum glucose and insulin, serum lipids
Nabuco et al. 2019c (62)	Lean body mass, muscle strength, physical performance, blood pressure, serum glucose and insulin, serum lipids
Ottestad et al. 2017 (63)	Lean body mass, muscle strength, physical performance, serum glucose and insulin, serum lipids, kidney function
Park et al. 2018 (64)	Lean body mass, muscle strength, physical performance, serum glucose and insulin, serum lipids, kidney function, cognition
Ten Haaf et al. 2019 (65)	Lean body mass, muscle strength, physical performance, kidney function
Thomson et al. 2016 (66)	Lean body mass, muscle strength, physical performance
Wright et al. 2018 (67)	Lean body mass, blood pressure, serum glucose and insulin, serum lipids

^1^RCT, randomized controlled trial.

2 Publications listed in the same row report on the same RCT.

### Study characteristics

The characteristics and results of the included RCTs are (per outcome) summarized in **Tables 3–11** and described in detail in **Supplemental Tables 1–9**. The 18 RCTs in the present SR included in total >1300 participants, ranging between 12 (47) and 219 (52) participants per RCT. In all but 1 RCT (66) the mean age of the participants was ≥65 y. Most RCTs included both men and women (44, 45, 47, 48, 55, 56, 63–67), 5 RCTs included only women (49–54, 59–62), and 3 RCTs included only men (46, 57, 58). Six RCTs were conducted in the United States (46–49, 56, 67), 1 RCT in Canada (57), 4 RCTs (5 publications) in Europe (44, 45, 55, 63, 65), 2 RCTs (4 publications) in Australia (52–54, 66), 1 RCT in New Zealand (58), 3 RCTs (6 publications) in Brazil (50, 51, 59–62), and 1 RCT in Korea (64). Publication year of the included RCTs ranged from 1995 to 2019, with the majority of studies (*n* = 17) published since 2015.

#### Protein intake

In most RCTs (*n* = 15), mean habitual protein intake was between 0.8 and 1.1 g/(kg BW ⋅ d). More specifically, mean habitual protein intake was ≥0.8 to <0.9 g/(kg BW · d) in 5 RCTs (7 publications) (44–47, 50, 51, 67), ≥0.9 to <1.0 g/(kg BW ⋅ d) in another 5 RCTs (48, 58, 63–65), and ≥1.0 to <1.1 g/(kg BW ⋅ d) in the remaining 5 RCTs (7 publications) (55, 56, 59–62, 66). Mean habitual protein intake was ≥1.1 g/(kg BW ⋅ d) in 1 RCT (3 publications) (52–54). In 2 RCTs (49, 57), the habitual protein intake was unclear but judged as not <0.8 g/(kg BW ⋅ d) based on the subjects having health characteristics similar to those who participated in the other RCTs. Nine of the 18 RCTs (13 publications) were performed in the context of a concomitant physical exercise intervention (44, 45, 47, 48, 50, 51, 57, 59–62, 65, 66), mostly resistance exercise training. RCTs with and without concomitant physical exercise were available for all outcomes except cognition. The type of protein intervention was protein supplements or amino acid supplements (powders or pills) in 11 RCTs (17 publications) (44, 45, 48–56, 59–62, 64, 65), protein-(en)rich(ed) foods in 4 RCTs (47, 57, 63, 66), high-protein diets in 2 RCTs (58, 67), and a combination of protein supplements and protein-(en)rich(ed) foods in 1 RCT (46).

#### Risk of bias

None of the studies had a low risk of bias, for 50% of the studies there were some concerns regarding the risk of bias and 50% of the studies had a high risk of bias (**Supplemental Table 10**). The most prevalent limitations were the following: *1*) lack of information on randomization of allocation sequence and/or blinding of staff and participants; *2*) lack of information on blinding of outcome assessors; and *3*) missing outcome data, without analyses performed to demonstrate that the result had not been influenced by those missing data.

#### Statistical power

**Supplemental Table 11** specifies the outcome(s) on which the power analysis was based in each RCT. The power analysis was most often based on lean body mass, followed by muscle strength, physical performance, and bone health. In 8 RCTs (11 publications) information on study power was not reported (47, 49–51, 55, 57) or unclear (59–62, 67).

### Results for the effect of increased protein intake on health outcomes

#### Lean body mass

The evaluation of the effect of increased protein intake on lean body mass in older adults included 18 RCTs (21 publications) (44, 46–51, 54–67), with a total of 61 statistically tested contrasts. The characteristics and results of those RCTs are summarized in Table 3 and described in detail in Supplemental Table 1. Assessed outcome measures included, among others, appendicular lean soft tissue (LST), total lean body mass, and muscle cross-sectional area. The risk of bias was scored as “some concerns” (*n* = 9) or “high” (*n* = 9).

**TABLE 3:** Overview of the results of the 18 evaluated RCTs on the effect of increased protein intake on lean body mass in older adults, categorized according to habitual protein intake and ordered by protein dose^1^

	Analytic *n* IG/CG	Total protein intake [g/(kg BW · d)] during intervention^2^	Protein dose^3^ [g/(kg BW · d)]	Protein type^4^	With/without physical exercise	Risk of bias^5^	Outcome measure	Result^6^				
Study								+	NS	−	?	Comments
Habitual protein intake (reference): ≥0.8 to <0.9 g/(kg BW · d)												
Arnarson et al. 2013 (44)	75/66	IG: 1.06 ± 0.23; CG 0.89 ± 0.23	0.17	A	Ex	H	Total LBM^7^		✔			
							aSMM^7^		✔			
Bhasin et al. 2018 (46)	42/39	IG: 1.17 ± 0.13; CG: 0.81 ± 0.10	0.36	A, B	NoEx	SC	Total LBM^7^		✔*			*P = 0.04 for relative total LBM (% of BW), mainly due to greater decrease in total fat mass (kg) in IG than CG (*P* = 0.02)
							Trunk LBM^7^		✔			
							aLBM^7^		✔			
Sugihara Junior et al. 2018 (51), Fernandes et al. 2018 (50)	15/16	IG: 1.4 ± 0.1; CG: 0.87 ± 0.1	0.53	A	Ex	H	Upper-limb LST		✔			
							Lower-limb LST		✔			
							SMM	✔				
							Total LST	✔				
Wright et al. 2018 (67)	12/10	IG: 1.4; CG: 0.8 (prescribed)^9,10^	0.6^9^	C	NoEx	H	Total LBM	✔*				*No significant change in total fat mass (*P* > 0.05)
							Trunk LBM	✔				
							aLBM		✔			
							Muscle CSA, thigh		✔			
							Muscle volume, thigh		✔			
							Muscle CSA, calf		✔			
							Muscle volume, calf		✔			
Campbell et al. 1995 (47)	6/6	IG: 1.62 ± 0.02; CG: 0.80 ± 0.02	0.82	B	Ex	SC	Fat-free mass		✔			
							Muscle CSA, thigh		✔			
* Subtotal (contrasts)*								*4*	*14*	*0*	*0*	*Beneficial effect observed for 4 of 18 contrasts*
* Subtotal (studies)* ^8^								*2*	*5*	*0*	*0*	*Beneficial effect observed in 2 of 5 studies*
Habitual protein intake (reference): ≥0.9 to <1.0 g/(kg BW ⋅ d)												
Park et al. 2018 (64)	40/40	IG1: 1.18 ± 0.23; CG: 0.90 ± 0.38	0.28	A	NoEx	SC	aSMM^7^		✔			
							aSMM relative to BW^7^		✔			
							aSMM relative to squared height^7^		✔			
							aSMM relative to BMI^7^		✔			
	40/40	IG2: 1.37 ± 0.26; CG: 0.90 ± 0.38	0.47				aSMM^7^	✔				
							aSMM relative to BW^7^	✔				
							aSMM relative to squared height^7^	✔				
							aSMM relative to BMI^7^	✔				
Ten Haaf et al. 2019 (65)	58/56	IG: 0.92 ± 0.27 (without protein supplementation of 31 g/d); CG: 0.97 ± 0.23	0.36^9^	A	Ex	SC	Total LBM^7^		✔*			*P = 0.046 for relative total LBM (% of BW), mainly due to greater decrease in total fat mass (kg) in IG than in CG (*P* = 0.013)
Chalé et al. 2013 (48)	42/38	NR (baseline: 0.98)	0.38^11^	A	Ex	SC	Total LBM^7^		✔			
							Muscle CSA, thigh^7^		✔			
Ottestad et al. 2017 (63)	17/19	IG: 1.4 ± 0.5; CG: 0.9 ± 0.4	0.5	B	NoEx	H	Total LBM		✔			
							Trunk LBM		✔			
							aLBM		✔			
Mitchell et al. 2017 (58)	14/15	IG: 1.7 ± 0.1; CG: 0.9 ± 0.1	0.8	C	NoEx	H	Total LBM	✔*				*No significant change in BW (*P* = 0.174), greater decrease in total and % fat mass in IG than CG (both *P* < 0.01)
							Trunk LBM	✔				
							aLBM	✔				
							Muscle CSA, thigh		✔			
* Subtotal (contrasts)*								*7*	*11*	*0*	*0*	*Beneficial effect observed for 7 of 18 contrasts*
* Subtotal (studies)* ^8^								*2*	*5*	*0*	*0*	*Beneficial effect observed in 2 of 5 studies*
Habitual protein intake (reference): ≥1.0 to <1.1 g/(kg BW · d)												
Ispoglou et al. 2016 (55)	8/9	1.02–1.08 (without protein supplementation of ∼0.21 g/[kg BW ⋅ d) in IG1]	0.21	A	NoEx	H	Total LTM		✔			
	8/9	1.02–1.08 (without protein supplementation of ∼0.21 g/[kg BW ⋅ d) in IG2]	0.21				Total LTM		✔			
Nabuco et al. 2019c (62)	13/13	IG: 1.0 ± 0.23 (without ∼35 g whey protein supplementation 3 d/wk); CG: 1.0 ± 0.19	0.24^9^	A	Ex	SC	Total LST	✔				
							Lower LST	✔				
							aLST	✔				
Kerstetter et al. 2015 (56)	105/102	IG: 1.30 ± 0.05; CG: 1.05 ± 0.04	0.25	A	NoEx	SC	Total LBM		✔*			*P = 0.069 (total LBM tended to decrease less in IG than in CG)
							Trunk LBM	✔*				*No significant change in total fat mass (*P* > 0.05)
Thomson et al. 2016 (66)	34/23	IG1: 1.42 ± 0.14; CG: 1.08 ± 0.05	0.34	B	Ex	H	Total LBM		✔			
	26/23	IG2: 1.45 ± 0.14; CG: 1.08 ± 0.05	0.37				Total LBM		✔			
Nabuco et al. 2018 (59), 2019a (60), 2019b (61)	22/23	IG1: 1.38 ± 0.26; CG: 1.0 ± 0.25	0.38	A	Ex	SC	Upper-limb LST		✔			
							Lower-limb LST	✔				
							SMM	✔				
							aLST		✔			
							Total LST	✔				
	21/23	IG2: 1.49 ± 0.46; CG: 1.0 ± 0.25	0.49				Upper-limb LST		✔			
							Lower-limb LST	✔				
							SMM	✔				
							aLST		✔			
							Total LST	✔				
*Subtotal (contrasts)*								*10*	*9*	*0*	*0*	*Beneficial effect observed for 10 of 19 contrasts*
*Subtotal (studies)*^8^								*3*	*4*	*0*	*0*	*Beneficial effect observed in 3 of 5 studies*
Habitual protein intake (reference): ≥1.1 g/(kg BW ⋅ d)												
Zhu et al. 2015 (54)	93/88 (2-y follow-up)	IG: 1.4 ± 0.4; CG: 1.1 ± 0.4	0.3	A	NoEx	SC	Arm LBM^7^		✔			
							Leg LBM^7^		✔			
							aLBM^7^		✔			
							aLBM relative to squared height^7^		✔			
							Muscle CSA, calf^7^		✔			
*Subtotal (contrasts)*								*0*	*5*	*0*	*0*	*No effect observed for any of 5 contrasts*
*Subtotal (studies)*^8^								*0*	*1*	*0*	*0*	*No effect observed in the single study*
Habitual protein intake (reference): Unclear												
Dillon et al. 2009 (49)	7/7	NR	0.20	A	NoEx	H	Total LBM		✔*			*Results for time*group interaction (ANOVA) not reported, suggests protein has no effect
Mitchell et al. 2015 (57)	16 (total)	NR	NR (15 g/d)	B	Ex	H	Muscle fiber area		✔			
*Subtotal (contrasts)*								*0*	*2*	*0*	*0*	*No effect observed for any of 2 contrasts*
* Subtotal (studies)* ^8^								*0*	*2*	*0*	*0*	*No effect observed in either study*
* Total (contrasts)*								*21*	*41*	*0*	*0*	*Beneficial effect observed for 21 of 62 contrasts*
*Total (studies)*^8^								*7*	*17*	*0*	*0*	*Beneficial effect observed in 7 of 18 studies*

1 aLBM, appendicular lean body mass; aLST, appendicular lean soft tissue; aSMM, appendicular skeletal muscle mass; BW, body weight; CG, control group; CSA, cross-sectional area; Ex, with concomitant exercise intervention; H, high risk of bias; IG, intervention group; L, low risk of bias; LBM, lean body mass; LST, lean soft tissue; LTM, lean tissue mass; NoEx, without concomitant exercise intervention; NR, not reported; NS, not significant; RoB 2, revised Cochrane risk of bias tool for randomized trials; SC, some concerns (regarding risk of bias); SMM, skeletal muscle mass; *, the result is accompanied by an explanation (see Comments).

2 Total protein intake during follow-up. If protein intake was assessed at multiple time points, the intake assessed at the final time point was considered.

3 ”Protein dose” indicates the difference in achieved total protein intake between the intervention group and the control group during follow-up (which is not necessarily equal to supplemented/prescribed amount of protein).

4 ”Protein type” indicates the way in which a higher protein intake was achieved and is categorized into protein or amino acid supplements (A), 1 or a few protein-(en)rich(ed) foods (B), or high-protein diets (C).

5 Risk of bias was assessed using the RoB 2 Cochrane collaboration tool and scored as “low” (L), “some concerns” (SC) or “high” (H).

6 The results of the studies are indicated as follows: +, statistically significant beneficial effect (*P* < 0.05); −, statistically significant unfavorable effect (*P* < 0.05); NS, no statistically significant effect (*P* ≥ 0.05); ?, result unclear. In cases where results were reported for multiple time points, only the result for the final time point is reported.

7 Sufficient statistical power to detect an effect is to be expected, based on the sample size calculation.

8 Some studies assessed multiple specific outcomes (i.e., multiple contrasts) for the health outcome “lean body mass,” so 1 study can show both a statistically significant and a nonsignificant effect.

9 Protein intake in g/(kg BW · d) was calculated by using protein intake in g/d and mean BW (and compliance, if available).

10 Actual protein intake may have been different from the prescribed protein intake, due to noncompliance (compliance was 91% on average).

11 (Achieved) protein dose was estimated using prescribed protein dose, compliance rate (72%), and mean BW.

In 7 of the 18 RCTs (39%) a beneficial effect of increased protein intake on lean body mass was found for ≥1 of the statistically tested contrasts [21 of 62 contrasts (34%)] (50, 51, 56, 58, 59, 61, 62, 64, 67). Of the RCTs showing a statistical beneficial effect, 3 RCTs (5 publications) expressed effect sizes as relative change in lean body mass from baseline (50, 51, 59, 61, 62). Those 3 RCTs included a total of 123 participants, all from Brazil, and were performed in the context of physical exercise. Between-group mean differences ranged from 1.2% in total LST [at a protein dose of 0.38 g/(kg BW ⋅ d)] (61) to 3.7% in appendicular LST [at a protein dose of 0.24 g/(kg BW ⋅ d)] (62) in favor of the intervention groups after 12 wk. The 2 RCTs that showed a statistical beneficial effect and reported absolute changes in lean body mass after 10 to 12 wk included a total of 51 participants from the United States or New Zealand and were both not performed in the context of physical exercise (58, 67). Mean differences ranged from 0.8 kg in trunk lean body mass [at a protein dose of 0.6 g/(kg BW ⋅ d)] (67) to 2.0 kg in total lean body mass [at a protein dose of 0.8 g/(kg BW ⋅ d)] (58). No unfavorable effects on lean body mass were observed. The changes in lean body mass did not involve any statistically significant change in BW (**Supplemental Table 12**). In some cases, it was shown that the greater increase in (relative) lean body mass was a result of a significantly greater decrease in fat mass.

#### Muscle strength

The evaluation of the effect of increased protein intake on muscle strength in older adults included 15 RCTs (44, 46, 48, 49, 51, 54, 55, 57–59, 62–66), with a total of 83 statistically tested contrasts (Table 4; Supplemental Table 2). Assessed outcome measures included, among others, handgrip strength, chess press strength, and knee extension peak power. The risk of bias was scored as “some concerns” (*n* = 7) or “high” (*n* = 8).

**TABLE 4:** Overview of the results of the 15 evaluated RCTs on the effect of increased protein intake on muscle strength in older adults, categorized according to habitual protein intake and ordered by protein dose^1^

	Analytic *n* IG/CG	Total protein intake [g/(kg BW · d)] during intervention^2^	Protein dose^3^ [g/(kg BW · d)]	Protein type^4^	With/without physical exercise	Risk of bias^5^	Outcome measure	Result^6^				
Study								+	NS	−	?	Comments
Habitual protein intake (reference): ≥0.8 to <0.9 g/(kg BW · d)												
Arnarson et al. 2013 (44)	75/66	IG: 1.06 ± 0.23; CG 0.89 ± 0.23	0.17	A	Ex	H	Quadriceps strength		✔			
Bhasin et al. 2018 (46)	29–31^†^/32–34^†^	IG: 1.17 ± 0.13; CG: 0.81 ± 0.10	0.36	A,B	NoEx	SC	Leg press strength		✔			
							Chest press strength		✔			
							Leg press peak power		✔			
Sugihara Junior et al. 2018 (51)	15/16	IG: 1.4 ± 0.1; CG: 0.87 ± 0.1	0.53	A	Ex	H	Chest press strength	✔				
							Knee extension strength	✔				
							Preacher curl strength		✔*			*P = 0.07 (strength tended to increase more in IG than in CG)
							Total strength^7^	✔				
							Lower-limb muscle quality index^8^		✔			
							Upper-limb muscle quality index^9^		✔			
							Total muscle quality index^10^		✔			
* Subtotal (contrasts)*								*3*	*8*	*0*	*0*	*Beneficial effect observed for 3 of 11 contrasts*
*Subtotal (studies)*^11^								*1*	*3*	*0*	*0*	*Beneficial effect observed in 1 of 3 studies*
Habitual protein intake (reference): ≥0.9 to <1.0 kg BW/d												
Park et al. 2018 (64)	40/40	IG1: 1.18 ± 0.23; CG: 0.90 ± 0.38	0.28	A	NoEx	SC	Handgrip strength (IG1 vs. CG)		✔			
	40/40	IG2: 1.37 ± 0.26; CG: 0.90 ± 0.38	0.47				Handgrip strength (IG2 vs. CG)		✔			
Ten Haaf et al. 2019 (65)	58/56 for handgrip strength; 22–56^†^ (total) for other outcome measures	IG: 0.92 ± 0.27 (without protein supplementation of 31 g/d); CG: 0.97 ± 0.23	0.36^12^	A	Ex	SC	Handgrip strength^§^		✔			
							Quadriceps MVC^§^		✔			
							Maximal rate of force rise, quadriceps^§^		✔			
							Early relaxation time, quadriceps^§^		✔			
							Half relaxation time, quadriceps^§^		✔			
							Fatigue^§^		✔			
Chalé et al. 2013 (48)	42/38	NR (baseline: 0.98)	0.38^13^	A	Ex	SC	Double leg press strength, 1 RM^§^		✔			
							Knee extension, 1 RM, right^§^		✔			
							Knee extension, 1 RM, left^§^		✔			
							Double leg press peak power, 40% 1 RM^§^		✔			
							Knee extension peak power, 40% 1 RM, right^§^	✔				
							Knee extension peak power, 40% 1 RM, left^§^	✔				
							Double leg press peak power, 70% 1 RM^§^		✔			
							Knee extension peak power, 70% 1 RM, right^§^	✔				
							Knee extension peak power, 70% 1 RM, left^§^	✔				
Ottestad et al. 2017 (63)	16–17^†^/18–19^†^	IG: 1.4 ± 0.5; CG: 0.9 ± 0.4	0.5	B	NoEx	H	Leg press strength		✔			
							Chest press strength		✔			
							Handgrip strength, dominant		✔			
							Handgrip strength, nondominant		✔			
Mitchell et al. 2017 (58)	14/15	IG: 1.7 ± 0.1; CG: 0.9 ± 0.1	0.8	C	NoEx	H	Hand grip strength		✔			
							Knee extension MVC		✔			
							Knee extension peak power	✔				
*Subtotal (contrasts)*								*5*	*19*	*0*	*0*	*Beneficial effect observed for 5 of 24 contrasts*
*Subtotal (studies)*^11^								*2*	*5*	*0*	*0*	*Beneficial effect observed in 2 of 5 studies*
Habitual protein intake (reference): ≥1.0 to <1.1 kg BW/d												
Ispoglou et al. 2016 (55)	8/9	1.02–1.08 [without protein supplementation of ∼0.21 g/(kg BW · d) in IG1]	0.21	A	NoEx	H	Handgrip strength		✔			
							30-s arm-curl test		✔			
	8/9	1.02–1.08 [without protein supplementation of ∼0.21 g/(kg BW · d) in IG2]	0.21				Handgrip strength		✔			
							30-s arm-curl test		✔			
Nabuco et al. 2019c (62)	13/13	IG: 1.0 ± 0.23 (without ∼35 g whey protein supplementation on 3 d/wk); CG: 1.0 ± 0.19	0.24^12^	A	Ex	SC	Knee extension		✔			
							Chest press		✔			
							Preacher curl		✔			
							Total strength^8^		✔			
Thomson et al. 2016 (66)	34/23	IG1: 1.42 ± 0.14; CG: 1.08 ± 0.05	0.34	B	Ex	H	Knee extensor strength		✔			
							Handgrip strength		✔			
							Leg press		✔			
							Chest press		✔			
							Knee extension strength		✔			
							Lat pull-down				✔*	*Smaller % (but not absolute) increase in IG1 than in CG
							Leg curl		✔			
							Total 8RM		✔			
	26/23	IG2: 1.45 ± 0.14; CG: 1.08 ± 0.05	0.37				Knee extensor strength		✔*			*P = 0.08 (strength tended to increase less in IG2 than in CG)
							Handgrip strength		✔			
							Leg press			✔		
							Chest press		✔			
							Knee extension strength		✔			
							Lat pull-down		✔			
							Leg curl		✔			
							Total 8RM			✔		
Nabuco et al. 2018 (59)	22/23	IG1: 1.38 ± 0.26; CG: 1.0 ± 0.25	0.38	A	Ex	SC	Chest press	✔				
							Knee extension	✔				
							Preacher curl		✔			
							Total strength^8^	✔				
	21/23	IG2: 1.49 ± 0.46; CG: 1.0 ± 0.25	0.49				Chest press	✔				
							Knee extension	✔				
							Preacher curl		✔			
							Total strength^8^	✔				
* Subtotal (contrasts)*								*6*	*23*	*2*	*1*	*Beneficial effect observed for 6 of 32 contrasts; unfavorable effect observed for 2 of 32 contrasts*
*Subtotal (studies)*^11^								*1*	*4*	*1*	*1*	*Beneficial effect observed in 1 of 4 studies; unfavorable effect observed in 1 of 4 studies*
Habitual protein intake (reference): ≥1.1 kg BW/d												
Zhu et al. 2015 (54)	93/88 (2-y follow-up)	IG: 1.4 ± 0.4; CG: 1.1 ± 0.4	0.3	A	NoEx	SC	Handgrip strength		✔			
							Ankle dorsiflexion strength		✔			
							Knee flexor strength		✔			
							Knee extensor strength		✔			
							Hip extensor strength		✔			
							Hip abductor strength		✔			
							Hip flexor strength		✔			
							Hip adductor strength		✔			
* Subtotal (contrasts)*								*0*	*8*	*0*	*0*	*No effect observed for any of 8 contrasts*
* Subtotal (studies)* ^11^								*0*	*1*	*0*	*0*	*No effect observed in the single study*
Habitual protein intake (reference): Unclear												
Dillon et al. 2009 (49)	7/7	NR	0.20	A	NoEx	H	Biceps curl		✔*			*Results for time*group interaction (ANOVA) not reported, which suggests that protein has no effect
							Triceps extension		✔*			*Results for time*group interaction (ANOVA) not reported, which suggests that protein has no effect
							Leg extension		✔*			*Results for time*group interaction (ANOVA) not reported, which suggests that protein has no effect
							Leg curl		✔*			*Results for time*group interaction (ANOVA) not reported, which suggests that protein has no effect
Mitchell et al. 2015 (57)	16 (total)	NR	NR (15 g/d)	B	Ex	H	Knee extension isometric MVC		✔			
							Leg press		✔			
							Leg extension		✔			
							Chest press		✔			
*Subtotal (contrasts)*								*0*	*8*	*0*	*0*	*No effect observed for any of 8 contrasts*
* Subtotal (studies)* ^11^								*0*	*2*	*0*	*0*	*No effect observed in either study*
*Total (contrasts)*								*14*	*66*	*2*	*1*	*Beneficial effect observed for 14 of 83 contrasts; unfavorable effect observed for 2 of 83 contrasts*
*Total (studies)*^11^								*4*	*15*	*1*	*1*	*Beneficial effect observed in 4 of 15 studies; unfavorable effect observed in 1 of 15 studies*

^†^Depending on specific outcome measure.

^§^Sufficient statistical power to detect an effect is to be expected, based on the sample size calculation.

1 BW, body weight; CG, control group; Ex, with concomitant exercise intervention; H, high risk of bias; IG, intervention group; L, low risk of bias; MVC, maximal voluntary contraction; NoEx, without concomitant exercise intervention; NR, not reported; NS, not significant; RM, repetition maximum; SC, some concerns (regarding risk of bias) *, the result is accompanied by an explanation (see Comments).

2 Total protein intake during follow-up. If protein intake was assessed at multiple time points, the intake assessed at the final time point was considered.

3 ”Protein dose” indicates the difference in achieved total protein intake between the intervention group and the control group during follow-up (which is not necessarily equal to supplemented/prescribed amount of protein).

4 ”Protein type” indicates the way in which a higher protein intake was achieved and is categorized into protein or amino acid supplements (A), 1 or a few protein-(en)rich(ed) foods (B), or high-protein diets (C).

5 Risk of bias was assessed using the RoB 2 Cochrane collaboration tool and scored as “low” (L), “some concerns” (SC) or “high” (H).

6 The results of the studies are indicated as follows: +, statistically significant beneficial effect (*P* < 0.05); −, statistically significant unfavorable effect (*P* < 0.05); NS, no statistically significant effect (*P* ≥ 0.05); ?, result unclear. In cases where results were reported for multiple time points, only the result for the final time point is reported.

7 Total strength was calculated as the sum of chest press, knee extension and preacher curl strength (kg).

8 Lower-limb muscle quality index was calculated as knee extension strength divided by lower-limb lean soft tissue.

9 Upper-limb muscle quality index was calculated as preacher curl strength divided by upper-limb lean soft tissue.

10 Total muscle quality index was calculated as total strength divided by skeletal muscle mass.

11 Some studies assessed multiple specific outcomes (i.e., multiple contrasts) for the health outcome “muscle strength,” so 1 study can show both a significant and a nonsignificant effect.

12 Protein intake in g/(kg BW ⋅ d) was calculated by using protein intake in g/d and mean BW (and compliance, if available).

13 (Achieved) protein dose was estimated using prescribed protein dose, compliance rate (72%), and mean BW.

In 4 of the 15 RCTs (27%) a beneficial effect of increased protein intake on muscle strength was found for ≥1 of the statistically tested contrasts [14 of 83 contrasts (17%)] (48, 51, 58, 59). Of the RCTs showing a statistical beneficial effect, 2 RCTs expressed effect sizes as relative change in muscle strength from baseline (51, 59). Those 2 RCTs included 97 participants, all from Brazil, and were performed in the context of physical exercise. Between-group mean differences ranged from 1.1% in chest press strength [1 RM; at a protein dose of 0.38 g/(kg BW ⋅ d)] (57) to 4.1% in knee extension strength [1 RM; at a protein dose of 0.53 g/(kg BW ⋅ d)] (54) after 12 wk. The 2 RCTs that showed a statistically significant beneficial effect and reported absolute changes in muscle strength (48, 58) included 109 participants from the United States or New Zealand. Reported between-group mean differences in, for example, knee extension peak power were 38 W after 10 wk [at a protein dose of 0.8 g/(kg BW ⋅ d); not in the context of physical exercise] (58) and 15 W after 6 mo (at a protein dose of 0.38 g/(kg BW ⋅ d); in the context of physical exercise) (48).

An unfavorable effect on muscle strength was observed in 1 RCT (66), which was performed in the context of physical exercise [1 of 8 RCTs (13%); 2 of 55 contrasts (4%)]. This unfavorable effect was observed for 2 specific outcome measurements [i.e., leg press strength and total strength (8 RM)], and only for the group receiving soy protein (*n* = 26) and not for the group receiving a comparable amount of dairy protein (*n* = 34), as compared with the control group (*n* = 23). The reported mean difference in change in leg press strength (8 RM) from baseline was 18.9 kg (70.2%) in favor of the control group compared with the soy protein group after 12 wk. The unfavorable effect of soy protein observed in this RCT was, according to the authors of the original study, most likely attributable to the isoflavones in soy foods that might attenuate the anabolic muscle response through reducing testosterone concentrations. Since the unfavorable effect is likely due to the type of protein and not to the amount of protein, the committee gave less weight to this result.

#### Physical performance

The evaluation of the effect of increased protein intake on physical performance in older adults included 12 RCTs (44, 46, 48, 54, 55, 58, 59, 62–66), with a total of 44 statistically tested contrasts (Table 5; Supplemental Table 3). Almost all RCTs used an objective measure of physical performance, such as gait speed, the Short Physical Performance Battery (SPPB), or the Timed Up and Go (TUG) test. The risk of bias was scored as “some concerns” (*n* = 7) or “high” (*n* = 5).

**TABLE 5:** Overview of the results of the 12 evaluated RCTs on the effect of increased protein intake on physical performance in older adults, categorized according to habitual protein intake and ordered by protein dose^1^

	Analytic *n* IG/CG	Total protein intake [g/(kg BW · d)] during intervention^2^	Protein dose^3^ [g/(kg BW · d)]	Protein type^4^	With/without physical exercise	Risk of bias^5^	Outcome measure	Result^6^				
Study								+	NS	−	?	Comments
Habitual protein intake (reference): ≥0.8 to <0.9 kg BW/d												
Arnarson et al. 2013 (44)	75/66	IG: 1.06 ± 0.23; CG 0.89 ± 0.23	0.17	A	Ex	H	Gait speed, 6-min		✔			
							TUG		✔			
Bhasin et al. 2018 (46)	33–42^†^/32–40^†^	IG: 1.17 ± 0.13; CG: 0.81 ± 0.10	0.36	A,B	NoEx	SC	Gait speed, 6-min		✔			
							Gait speed, 50-meter		✔			
							Stair climb power, unloaded		✔*			*P = 0.08 (power tended to increase less in IG than in CG)
							Stair climb power, loaded		✔			
							Perceived physical function		✔			
* Subtotal (contrasts)*								*0*	*7*	*0*	*0*	*No effect observed for any of 7 contrasts*
* Subtotal (studies)* ^7^								*0*	*2*	*0*	*0*	*No effect observed for either study*
Habitual protein intake (reference): ≥0.9 to <1.0 kg BW/d												
Park et al. 2018 (64)	40/40	IG1: 1.18 ± 0.23; CG: 0.90 ± 0.38	0.28	A	NoEx	SC	SPPB		✔			
							Gait speed, 4-meter		✔			
							Standing balance		✔			
							Chair rise time		✔			
							TUG		✔			
	40/40	IG2: 1.37 ± 0.26; CG: 0.90 ± 0.38	0.47				SPPB		✔			
							Gait speed, 4-meter	✔				
							Standing balance		✔			
							Chair rise time		✔			
							TUG		✔			
Ten Haaf et al. 2019 (65)	58/56 (except chair rise time: total n = 111)	IG: 0.92 ± 0.27 (without protein supplementation of 31 g/d); CG: 0.97 ± 0.23	0.36^8^	A	Ex	SC	SPPB^§^		✔			
							Standing balance^§^		✔			
							Gait speed, 4-meter^§^		✔			
							Chair rise time^§^		✔			
							TUG^§^		✔			
Chalé et al. 2013 (48)	42/38	NR (baseline: 0.98)	0.38^9^	A	Ex	SC	Gait speed, 400-meter^§^		✔			
							Stair climb time^§^		✔			
							Chair rise time^§^		✔			
							SPPB^§^		✔			
Ottestad et al. 2017 (63)	16/15–17^†^	IG: 1.4 ± 0.5; CG: 0.9 ± 0.4	0.5	B	NoEx	H	Chair rise time		✔			
							Stair climb time, unloaded		✔			
							Stair climb time, loaded		✔			
Mitchell et al. 2017 (58)	14/15	IG: 1.7 ± 0.1; CG: 0.9 ± 0.1	0.8	C	NoEx	H	SPPB		✔			
							TUG		✔			
*Subtotal (contrasts)*								*1*	*23*	*0*	*0*	*Beneficial effect observed for 1 of 24 contrasts*
*Subtotal (studies)*^7^								*1*	*5*	*0*	*0*	*Beneficial effect observed in 1 of 5 studies*
Habitual protein intake (reference): ≥1.0 to <1.1 kg BW/d												
Ispoglou et al. 2016 (55)	8/9	1.02–1.08 [without protein supplementation of ∼0.21 g/(kg BW ⋅ d) in IG1]	0.21	A	NoEx	H	Gait speed, 6-min		✔			
							30-s chair-stand test		✔			
	8/9	1.02–1.08 [without protein supplementation of ∼0.21 g/(kg BW ⋅ d) in IG2]	0.21				Gait speed, 6-min		✔			
							30-s chair-stand test		✔			
Nabuco et al. 2019c (62)	13/13	IG: 1.0 ± 0.23 (without ∼35 g whey protein supplementation on 3 d/wk); CG: 1.0 ± 0.19	0.24^7^	A	Ex	SC	Gait speed, 10-meter		✔			
							Chair rise time		✔			
Thomson et al. 2016 (66)	34/23	IG1: 1.42 ± 0.14; CG: 1.08 ± 0.05	0.34	B	Ex	H	Gait speed, 6-min		✔			
	26/23	IG2: 1.45 ± 0.14; CG: 1.08 ± 0.05	0.37				Gait speed, 6-min		✔			
Nabuco et al. 2018 (59)	22/23	IG1: 1.38 ± 0.26; CG: 1.0 ± 0.25	0.38	A	Ex	SC	Gait speed, 10-meter at fast pace	✔				
							Chair rise time		✔			
	21/23	IG2: 1.49 ± 0.46; CG: 1.0 ± 0.25	0.49				Gait speed, 10-meter at fast pace	✔				
							Chair rise time		✔			
*Subtotal (contrasts)*								*2*	*10*	*0*	*0*	*Beneficial effect observed for 2 of 12 contrasts*
*Subtotal (studies)*^7^								*1*	*4*	*0*	*0*	*Beneficial effect observed in 1 of 4 studies*
Habitual protein intake (reference): ≥1.1 kg BW/d												
Zhu et al. 2015 (54)	93/88 (2-y follow-up)	IG: 1.4 ± 0.4; CG: 1.1 ± 0.4	0.3	A	NoEx	SC	TUG		✔			
*Subtotal (contrasts)*								*0*	*1*	*0*	*0*	*No effect observed for the single contrast*
*Subtotal (studies)*^7^								*0*	*1*	*0*	*0*	*No effect observed in the single study*
* Total (contrasts)*								*3*	*41*	*0*	*0*	*Beneficial effect observed for 3 of 44 contrasts*
* Total (studies)* ^7^								*2*	*12*	*0*	*0*	*Beneficial effect observed in 2 of 12 studies*

^†^Depending on specific outcome measure.

^§^Sufficient statistical power to detect an effect is to be expected, based on the sample size calculation.

1 ADL, activities of daily living; BW, body weight; CG, control group; Ex, with concomitant exercise intervention; H, high risk of bias; IG, intervention group; L, low risk of bias; NoEx, without concomitant exercise intervention; NR, not reported; NS, not significant; SC, some concerns (regarding risk of bias); SPPB, short physical performance battery; TUG, Timed Up and Go; *, the result is accompanied by an explanation (see Comments).

2 Total protein intake during follow-up. If protein intake was assessed at multiple time points, the intake assessed at the final time point was considered.

3 ”Protein dose” indicates the difference in achieved total protein intake between the intervention group and the control group during follow-up (which is not necessarily equal to supplemented/prescribed amount of protein).

4 ”Protein type” indicates the way in which a higher protein intake was achieved and is categorized into protein or amino acid supplements (A), 1 or a few protein-(en)rich(ed) foods (B), or high-protein diets (C).

5 Risk of bias was assessed using the RoB 2 Cochrane collaboration tool and scored as “low” (L), “some concerns” (SC) or “high” (H).

6 The results of the studies are indicated as follows: +, statistically significant beneficial effect (*P* < 0.05); −, statistically significant unfavorable effect (*P* < 0.05); NS, no statistically significant effect (*P* ≥ 0.05); ?, result unclear. In cases where results were reported for multiple time points, only the result for the final time point is reported.

7 Some studies assessed multiple specific outcomes (i.e., multiple contrasts) for the health outcome “physical performance,” so 1 study can show both a significant and a nonsignificant effect.

8 Protein intake in g/(kg BW · d) was calculated by using protein intake in g/d and mean BW (and compliance, if available).

9 (Achieved) protein dose was estimated using prescribed protein dose, compliance rate (72%), and mean BW.

In 2 of the 12 RCTs (17%) a beneficial effect of increased protein intake on physical performance was found for ≥1 of the statistically tested contrasts [3 of 44 contrasts (7%)], both concerning gait speed (59, 64). One of those RCTs included 65 participants from Brazil and was performed in the context of physical exercise (59). The other RCT included 80 participants and was not performed in the context of physical exercise (64). No unfavorable effects on physical performance were observed.

#### Bone health

Evaluation of the effect of increased protein intake on bone health was included in 4 RCTs (50, 53, 55, 56), with a total of 23 statistically tested contrasts (Table 6; Supplemental Table 4). Bone health was assessed as bone mineral density, as measured with DXA or CT, in the majority of the RCTs. One RCT assessed bone turnover markers. The risk of bias was scored as “some concerns” (*n* = 2) or “high” (*n* = 2).

**TABLE 6:** Overview of the results of the 4 evaluated RCTs on the effect of increased protein intake on bone health in older adults, categorized according to habitual protein intake and ordered by protein dose^1^

	Analytic *n* IG/CG	Total protein intake [g/(kg BW · d)] during intervention^2^	Protein dose^3^ [g/(kg BW · d)]	Protein type^4^	With/without physical exercise	Risk of bias^5^	Outcome measure	Result^6^				
Study								+	NS	−	?	Comments
Habitual protein intake (reference): ≥0.8 to <0.9 kg BW/d												
Fernandes et al. 2018 (50)	16/16	IG: 1.4 ± 0.1; CG: 0.87 ± 0.1	0.53	A	Ex	H	Total BMC		✔			
*Subtotal (contrasts)*								*0*	*1*	*0*	*0*	*No effect observed for the single contrast*
*Subtotal (studies)*^7^								*0*	*1*	*0*	*0*	*No effect observed in the single study*
Habitual protein intake (reference): ≥1.0 to <1.1 kg BW/d												
Ispoglou et al. 2016 (55)	8/9	1.02–1.08 [without protein supplementation of ∼0.21 g/(kg BW ⋅ d) in IG1]	0.21	A	NoEx	H	Total BMC		✔			
							Total BMD		✔			
	8/9	1.02–1.08 [without protein supplementation of ∼0.21 g/(kg BW ⋅ d) in IG2]	0.21				Total BMC		✔			
							Total BMD		✔			
Kerstetter et al. 2015 (56)	105–106^†^/102 for DXA measurements; 45/44 for QCT measurements; 61/60 for serum markers (all 18-mo follow-up)	IG: 1.30 ± 0.05; CG: 1.05 ± 0.04	0.25	A	NoEx	SC	BMD lumbar spine (DXA)^§^		✔			
							BMD total hip (DXA)^§^		✔			
							BMD femoral neck (DXA)^§^		✔			
							BMD lumbar spine (QCT)^§^		✔			
							BMD femoral neck, cortical (QCT)^§^		✔			
							BMD femoral neck, trabecular (QCT)^§^		✔			
							BMD femoral total, cortical (QCT)^§^		✔			
							BMD femoral total, trabecular (QCT)^§^		✔			
							Serum P1NP				✔*	*No difference at 18 mo, but at 9 mo serum P1NP increased more in IG than in CG (*P* = 0.0007); P1NP should be evaluated together with CTX to determine whether an effect is beneficial or unfavorable
							Serum CTX				✔*	*Serum CTX increased more in IG than in CG at 9 and 18 mo; CTX should be evaluated together with P1NP to determine whether an effect is beneficial or unfavorable
							Serum OC		✔			
* Subtotal (contrasts)*								*0*	*14*	*0*	*2*	*An effect observed for 2 of 15 contrasts*
* Subtotal (studies)* ^7^								*0*	*2*	*0*	*1*	*An effect observed in 1 of 2 studies*
Habitual protein intake (reference): ≥1.1 kg BW/d												
Zhu et al. 2011 (53)	95/88 for DXA measurements; 67/66 for QCT measurements (all 2-y follow-up)	IG: 1.4 ± 0.4; CG: 1.1 ± 0.4	0.3	A	NoEx	SC	Total hip aBMD (DXA)^§^		✔			
							Femoral neck aBMD (DXA)^§^		✔			
							Total hip volumetric BMD (QCT)^§^		✔			
							Femoral neck vBMD (QCT)^§^		✔			
							Femoral neck bone CSA (QCT)^§^		✔			
							Femoral neck buckling ratio (QCT)^§^		✔			
							Femoral neck polar CSMI (QCT)^§^		✔			
* Subtotal (contrasts)*								*0*	*7*	*0*	*0*	*No effect observed for any of 7 contrasts*
*Subtotal (studies)*^7^								*0*	*1*	*0*	*0*	*No effect observed in the single study*
*Total (contrasts)*								*0*	*22*	*0*	*2*	*An effect observed for 2 of 23 contrasts*
*Total (studies)*^7^								*0*	*4*	*0*	*1*	*An effect observed in 1 of 4 studies*

† Depending on specific outcome measure.

§ Sufficient statistical power to detect an effect is to be expected, based on the sample size calculation.

^1^aBMD, areal bone mineral density; BMC, bone mineral content; BMD, bone mineral density; BW, body weight; CG, control group; CSMI, cross-sectional moment of inertia; CTX, C-terminal telopeptide of type 1 collagen; Ex, with concomitant exercise intervention; H, high risk of bias; IG, intervention group; L, low risk of bias; NoEx, without concomitant exercise intervention; NR, not reported; NS, not significant; OC, osteocalcin; P1NP, N-terminal propeptides of type 1 procollagen; QCT, quantitative computed tomography; SC, some concerns (regarding risk of bias); vBMD, volumetric bone mineral density; *, the result is accompanied by an explanation (see Comments).

2 Total protein intake during follow-up. If protein intake was assessed at multiple time points, the intake assessed at the final time point was considered.

3 ”Protein dose” indicates the difference in achieved total protein intake between the intervention group and the control group during follow-up (which is not necessarily equal to supplemented/prescribed amount of protein).

4 ”Protein type” indicates the way in which a higher protein intake was achieved and is categorized into protein or amino acid supplements (A), 1 or a few protein-(en)riche(d) foods (B), or high-protein diets (C).

5 Risk of bias was assessed using the RoB 2 Cochrane collaboration tool and scored as “low” (L), “some concerns” (SC) or “high” (H).

6 The results of the studies are indicated as follows: +, statistically significant beneficial effect (*P* < 0.05); −, statistically significant unfavorable effect (*P* < 0.05); NS, no statistically significant effect (*P* ≥ 0.05); ?, result unclear. In cases where results were reported for multiple time points, only the result for the final time point is reported.

7 Some studies assessed multiple specific outcomes (i.e., multiple contrasts) for the health outcome “bone health,” so 1 study can show both a significant and a nonsignificant effect.

In 1 of the 4 RCTs (25%), an effect of increased protein intake on bone health was found for ≥1 of the statistically tested contrasts [2 of 23 contrasts (8%)] (56). In this RCT of US participants, the bone formation biomarker P1NP and the bone resorption biomarker CTX increased in the protein group (*n* = 61) compared with the control group (*n* = 60). No other effects on bone health were observed.

The committee noted that the percentage of effects based on contrasts (8%) was much lower than the percentage of effects based on studies (25%). The only effects observed were for surrogate outcomes, i.e., bone turnover markers. Furthermore, the effect estimates for the majority of tested contrasts were close to zero (Supplemental Table 4), suggesting no effect of increased protein intake on parameters of bone health. The committee judged that the totality of the evidence is too weak to conclude that there might be an effect on bone health.

#### Blood pressure

The evaluation of the effect of increased protein intake on (systolic or diastolic) blood pressure included in 4 RCTs (52, 60, 62, 67), with a total of 10 statistically tested contrasts (Table 7; Supplemental Table 5). The risk of bias was scored as “some concerns” (*n* = 3) or “high” (*n* = 1). None of those 4 RCTs showed an effect of increased protein intake on blood pressure.

**TABLE 7:** Overview of the results of the 4 evaluated RCTs on the effect of increased protein intake on blood pressure in older adults, categorized according to habitual protein intake and ordered by protein dose^1^

	Analytic *n* IG/CG	Total protein intake [g/(kg BW · d)] during intervention^2^	Protein dose^3^ [g/(kg BW · d)]	Protein type^4^	With/without physical exercise	Risk of bias^5^	Outcome measure	Result^6^				
Study								+	NS	−	?	Comments
Habitual protein intake (reference): ≥0.8 to <0.9 kg BW/d												
Wright et al. 2018 (67)	12/10	IG: 1.4; CG: 0.8 (prescribed)^7^, ^8^	0.6^7^	C	NoEx	H	SBP		✔			
							DBP		✔			
*Subtotal (contrasts)*								*0*	*2*	*0*	*0*	*No effect observed for any of 2 contrasts*
* Subtotal (studies)* ^9^								*0*	*1*	*0*	*0*	*No effect observed in the single study*
Habitual protein intake (reference): ≥1.0 to 1.1 kg BW/d												
Nabuco et al. 2019c (62)	13/13	IG: 1.0 ± 0.23 (without ∼35 g whey protein supplementation on 3 d/wk); CG: 1.0 ± 0.19	0.24^7^	A	Ex	SC	SBP		✔			
							DBP		✔			
Nabuco et al. 2019a (60)	22/23	IG1: 1.38 ± 0.26; CG: 1.0 ± 0.25	0.38	A	Ex	SC	SBP		✔			
							DBP		✔			
	21/23	IG2: 1.49 ± 0.46; CG: 1.0 ± 0.25	0.49				SBP		✔			
							DBP		✔			
* Subtotal (contrasts)*								*0*	*6*	*0*	*0*	*No effect observed for any of 6 contrasts*
*Subtotal (studies)*^9^								*0*	*2*	*0*	*0*	*No effect observed in either study*
Habitual protein intake (reference): ≥1.1 kg BW/d												
Hodgson et al. 2012 (52)	109/110	IG: 1.4 ± 0.4; CG: 1.1 ± 0.4	0.3	A	NoEx	SC	SBP^§^		✔			
							DBP^§^		✔			
*Subtotal (contrasts)*								*0*	*2*	*0*	*0*	*No effect observed for any of 2 contrasts (1 study)*
* Subtotal (studies)* ^9^								*0*	*1*	*0*	*0*	*No effect observed in the single study*
*Total (contrasts)*								*0*	*10*	*0*	*0*	*No effect observed for any of 10 contrasts*
*Total (studies)*^9^								*0*	*4*	*0*	*0*	*No effect observed in any of 4 studies*

§ Sufficient statistical power to detect an effect is to be expected, based on the sample size calculation.

^1^BW, body weight; CG, control group; DBP, diastolic blood pressure; Ex, with concomitant exercise intervention; H, high risk of bias; IG, intervention group; L, low risk of bias; NoEx, without concomitant exercise intervention; NR, not reported; NS, not significant; SBP, systolic blood pressure; SC, some concerns (regarding risk of bias).

2 Total protein intake during follow-up. If protein intake was assessed at multiple time points, the intake assessed at the final time point was considered.

3 ”Protein dose” indicates the difference in achieved total protein intake between the intervention group and the control group during follow-up (which is not necessarily equal to supplemented/prescribed amount of protein).

4 ”Protein type” indicates the way in which a higher protein intake was achieved and is categorized into protein or amino acid supplements (A), 1 or a few protein-(en)rich(ed) foods (B), or high-protein diets (C).

5 Risk of bias was assessed using the RoB 2 Cochrane collaboration tool and scored as “low” (L), “some concerns” (SC) or “high” (H).

6 The results of the studies are indicated as follows: +, statistically significant beneficial effect (*P* < 0.05); −, statistically significant unfavorable effect (*P* < 0.05); NS, no statistically significant effect (*P* ≥ 0.05); ?, result unclear. In cases where results were reported for multiple time points, only the result for the final time point is reported.

7 Protein intake in g/(kg BW · d) was calculated by using protein intake in g/d and mean BW.

8 Actual protein intake may have been different from the prescribed protein intake, due to noncompliance (compliance was 91% on average).

9 Some studies assessed multiple specific outcomes (i.e., multiple contrasts) for the health outcome “blood pressure,” so 1 study can show both a significant and a nonsignificant effect.

The committee noted that the sample size (*n* = 219) of the only RCT in which the power analysis was based on blood pressure (52) was substantially larger than the respective sample sizes of the other 3 RCTs (*n* = 22–45). Also, the committee noted that the effect sizes in those 3 studies were clinically relevant (i.e., reported between-group mean differences in systolic blood pressure were 5 mmHg or 2-4%) but did not reach statistical significance. Therefore, it was presumed that those 3 RCTs may have had insufficient statistical power to demonstrate an effect on blood pressure.

#### Serum glucose and insulin

The evaluation of the effect of increased protein intake on serum glucose and insulin included 6 RCTs (50, 60, 62–64, 67), with a total of 16 statistically tested contrasts (Table 8; Supplemental Table 6). Assessed outcome measures include fasting blood glucose, fasting insulin and HOMA-IR. The risk of bias was scored as “some concerns” (*n* = 3) or “high” (*n* = 3). None of those 6 RCTs showed an effect of increased protein intake on any parameter of glucose and insulin metabolism.

**TABLE 8:** Overview of the results of the 6 evaluated RCTs on the effect of increased protein intake on serum glucose and insulin in older adults, categorized according to habitual protein intake and ordered by protein dose^1^

	Analytic *n* IG/CG	Total protein intake [g/(kg BW · d)] during intervention^2^	Protein dose^3^ [g/(kg BW · d)]	Protein type^4^	With/without physical exercise	Risk of bias^5^	Outcome measure	Result^6^				
Study								+	NS	−	?	Comments
Habitual protein intake (reference): ≥0.8 to <0.9 g/(kg BW · d)												
Fernandes et al. 2018 (50)	16/16	IG: 1.4 ± 0.1; CG: 0.87 ± 0.1	0.53	A	Ex	H	Fasting blood glucose		✔			
Wright et al. 2018 (67)	12/10	IG: 1.4; CG: 0.8 (prescribed)^7^, ^8^	0.6^7^	C	NoEx	H	Fasting blood glucose		✔			
							Fasting insulin		✔			
							HOMA-IR		✔			
* Subtotal (contrasts)*								*0*	*4*	*0*	*0*	*No effect observed for any of 4 contrasts*
* Subtotal (studies)* ^9^								*0*	*2*	*0*	*0*	*No effect observed in either study*
Habitual protein intake (reference): ≥0.9 to <1.0 g/(kg BW · d)												
Park et al. 2018 (64)	40/40	IG1: 1.18 ± 0.23; CG: 0.90 ± 0.38	0.28									
	A	NoEx	SC	Fasting blood glucose		✔						
	40/40	IG2: 1.37 ± 0.26; CG: 0.90 ± 0.38	0.47				Fasting blood glucose		✔			
Ottestad et al. 2017 (63)	17/18	IG: 1.4 ± 0.5; CG: 0.9 ± 0.4	0.5	B	NoEx	H	Fasting blood glucose		✔			
* Subtotal (contrasts)*								*0*	*3*	*0*	*0*	*No effect observed for any of 3 contrasts*
* Subtotal (studies)* ^9^								*0*	*2*	*0*	*0*	*No effect observed in either study*
Habitual protein intake (reference): ≥1.0 to <1.1 g/(kg BW · d)												
Nabuco et al. 2019c (62)	13/13	IG: 1.0 ± 0.23 (without ∼35 g whey protein supplementation on 3 d/wk); CG: 1.0 ± 0.19	0.24^7^	A	Ex	SC	Fasting blood glucose		✔			
							Fasting insulin		✔			
							HOMA-IR		✔			
Nabuco et al. 2019a (60)	22/23	IG1: 1.38 ± 0.26; CG: 1.0 ± 0.25	0.38	A	Ex	SC	Fasting blood glucose		✔			
							Fasting insulin		✔			
							HOMA-IR		✔			
	21/23	IG2: 1.49 ± 0.46; CG: 1.0 ± 0.25	0.49				Fasting blood glucose		✔			
							Fasting insulin		✔			
							HOMA-IR		✔			
* Subtotal (contrasts)*								*0*	*9*	*0*	*0*	*No effect observed for any of 9 contrasts*
* Subtotal (studies)* ^9^								*0*	*2*	*0*	*0*	*No effect observed in either study*
*Total (contrasts)*								*0*	*16*	*0*	*0*	*No effect observed for any of 16 contrasts*
*Total (studies)*^9^								*0*	*6*	*0*	*0*	*No effect observed in any of 6 studies*

§ Sufficient statistical power to detect an effect is to be expected, based on the sample size calculation.

^1^BW, body weight; CG, control group; Ex, with concomitant exercise intervention; H, high risk of bias; IG, intervention group; L, low risk of bias; NoEx, without concomitant exercise intervention; NR, not reported; NS, not significant; SC, some concerns (regarding risk of bias).

2 Total protein intake during follow-up. If protein intake was assessed at multiple time points, the intake assessed at the final time point was considered.

3 ”Protein dose” indicates the difference in achieved total protein intake between the intervention group and the control group during follow-up (which is not necessarily equal to supplemented/prescribed amount of protein).

4 ”Protein type” indicates the way in which a higher protein intake was achieved and is categorized into protein or amino acid supplements (A), 1 or a few protein-(en)rich(ed) foods (B), or high-protein diets (C).

5 Risk of bias was assessed using the RoB 2 Cochrane collaboration tool and scored as “low” (L), “some concerns” (SC) or “high” (H).

6 The results of the studies are indicated as follows: +, statistically significant beneficial effect (*P* < 0.05); −, statistically significant unfavorable effect (*P* < 0.05); NS, no statistically significant effect (*P* ≥ 0.05); ?, result unclear. In cases where results were reported for multiple time points, only the result for the final time point is reported.

7 Protein intake in g/(kg BW · d) was calculated by using protein intake in g/d and mean BW.

8 Actual protein intake may have been different from the prescribed protein intake, due to noncompliance (compliance was 91% on average).

9 Some studies assessed multiple specific outcomes (i.e., multiple contrasts) for the health outcome “serum glucose and insulin, so 1 study can show both a significant and a nonsignificant effect.

The committee considered lack of statistical power as a possible explanation for no observed effects. In 2 of the 6 RCTs (63, 64), the power analysis was not based on serum glucose or insulin and it was unclear for the other 4 RCTs. The committee could, therefore, not exclude the possibility that increased protein intake affects serum glucose or insulin.

#### Serum lipids

The evaluation of the effect of increased protein intake on serum lipids included 7 RCTs (46, 50, 60, 62–64, 67), with a total of 43 statistically tested contrasts (Table 9; Supplemental Table 7). Assessed outcome measures include total cholesterol, LDL cholesterol, HDL cholesterol (or the ratio between 2 of those), and triglycerides. The risk of bias was scored as “some concerns” (*n* = 4) or “high” (*n* = 3).

**TABLE 9:** Overview of the results of the 7 evaluated RCTs on the effect of increased protein intake on serum lipids in older adults, categorized according to habitual protein intake and ordered by protein dose^1^

	Analytic *n* IG/CG	Total protein intake [g/(kg BW · d)] during intervention^2^	Protein dose^3^ [g/(kg BW · d)]	Protein type^4^	With/without physical exercise	Risk of bias^5^	Outcome measure	Result^6^				
Study								+	NS	−	?	Comments
Habitual protein intake (reference): ≥0.8 to <0.9 g/(kg BW · d)												
Bhasin et al. 2018 (46)	40–46^£^/38–46^£^	IG: 1.17 ± 0.13; CG: 0.81 ± 0.10	0.36	A,B	NoEx	SC	TC		✔			
							LDL-C		✔			
							HDL-C		✔			
							Triglycerides		✔*			*P = 0.055 (triglyceride levels tended to decrease more in IG than in CG)
Fernandes et al. 2018 (50)	16/16	IG: 1.4 ± 0.1; CG: 0.87 ± 0.1	0.53	A	Ex	H	TC		✔			
							LDL-C		✔			
							HDL-C		✔			
							Triglycerides		✔			
							TC/HDL-C ratio	✔				
							LDL/HDL-C ratio		✔			
Wright et al. 2018 (67)	12/10	IG: 1.4; CG: 0.8 (prescribed)^7^, ^8^	0.6^7^	C	NoEx	H	TC		✔			
							LDL-C			✔		
							HDL-C		✔			
							Triglycerides		✔			
							TC/HDL-C ratio		✔			
*Subtotal (contrasts)*								*1*	*13*	*1*	*0*	*Beneficial effect observed for 1 of 15 contrasts; unfavorable effect observed for 1 of 15 contrasts*
* Subtotal (studies)* ^9^								*1*	*3*	*1*	*0*	*Beneficial effect observed in 1 of 3 studies; unfavorable effect observed in 1 of 3 studies*
Habitual protein intake (reference): ≥0.9 to <1.0 g/(kg BW · d)												
Park et al. 2018 (64)	40/40	IG1: 1.18 ± 0.23; CG: 0.90 ± 0.38	0.28									
	A	NoEx	SC	TC		✔						
							LDL-C		✔			
							HDL-C		✔			
							Triglycerides		✔			
	40/40	IG2: 1.37 ± 0.26; CG: 0.90 ± 0.38	0.47				TC		✔			
							LDL-C		✔			
							HDL-C		✔			
							Triglycerides		✔			
Ottestad et al. 2017 (63)	16–17^†^/18	IG: 1.4 ± 0.5; CG: 0.9 ± 0.4	0.5	B	NoEx	H	TC		✔*			*P = 0.06 (total cholesterol tended to decrease more in IG than in CG)
							LDL-C		✔			
							HDL-C		✔			
							Triglycerides	✔				
* Subtotal (contrasts)*								*1*	*11*	*0*	*0*	*Beneficial effect observed for 1 of 12 contrasts*
*Subtotal (studies)*^9^								*1*	*2*	*0*	*0*	*Beneficial effect observed in 1 of 2 studies*
Habitual protein intake (reference): ≥1.0 to <1.1 g/(kg BW · d)												
Nabuco et al. 2019c (62)	13/13	IG: 1.0 ± 0.23 (without ∼35 g whey protein supplementation on 3 d/wk); CG: 1.0 ± 0.19	0.24^7^	A	Ex	SC	TC		✔			
							LDL-C		✔			
							HDL-C		✔			
							Triglycerides		✔			
Nabuco et al. 2019a (60)	22/23	IG1: 1.38 ± 0.26; CG: 1.0 ± 0.25	0.38	A	Ex	SC	TC		✔			
							LDL-C		✔			
							HDL-C		✔			
							Triglycerides		✔			
							TC/HDL-C ratio		✔			*P = 0.081 (TC/HDL-C ratio tended to increase more in IG1 than in CG)
							LDL/HDL-C ratio		✔			
	21/23	IG2: 1.49 ± 0.46; CG: 1.0 ± 0.25	0.49				TC		✔			
							LDL-C		✔			
							HDL-C		✔			
							Triglycerides		✔			
							TC/HDL-C ratio		✔			
							LDL/HDL-C ratio		✔			
*Subtotal (contrasts)*								*0*	*16*	*0*	*0*	*No effect observed for any of 16 contrasts*
*Subtotal (studies)*^9^								*0*	*2*	*0*	*0*	*No effect observed in either study*
* Total (contrasts)*								*2*	*40*	*1*	*0*	*Beneficial effect observed for 2 of 43 contrasts; unfavorable effect observed for 1 of 43 contrasts*
* Total (studies)* ^9^								*2*	*7*	*1*	*0*	*Beneficial effect observed in 2 of 7 studies; unfavorable effect observed in 1 of 7 studies*

£ The exact number of participants included in the analyses is not reported. The number must be between the number of participants who were randomized and the number of participants who completed the study.

† Depending on the specific outcome measure.

§ Sufficient statistical power to detect an effect is to be expected, based on the sample size calculation.

^1^BW, body weight; C, cholesterol; CG, control group; Ex, with concomitant exercise intervention; H, high risk of bias; IG, intervention group; L, low risk of bias; NoEx, without concomitant exercise intervention; NR, not reported; NS, not significant; SC, some concerns (regarding risk of bias); TC, total cholesterol; *, the result is accompanied by an explanation (see Comments).

2 Total protein intake during follow-up. If protein intake was assessed at multiple time points, the intake assessed at the final time point was considered.

3 ”Protein dose” indicates the difference in achieved total protein intake between the intervention group and the control group during follow-up (which is not necessarily equal to supplemented/prescribed amount of protein).

4 ”Protein type” indicates the way in which a higher protein intake was achieved and is categorized into protein or amino acid supplements (A), 1 or a few protein-(en)rich(ed) foods (B), or high-protein diets (C).

5 Risk of bias was assessed using the RoB 2 Cochrane collaboration tool and scored as “low” (L), “some concerns” (SC) or “high” (H).

6 The results of the studies are indicated as follows: +, statistically significant beneficial effect (*P* < 0.05); −, statistically significant unfavorable effect (*P* < 0.05); NS, no statistically significant effect (*P* ≥ 0.05); ?, result unclear. In cases where results were reported for multiple time points, only the result for the final time point is reported.

7 Protein intake in g/(kg BW · d) was calculated by using protein intake in g/d and mean BW.

8 Actual protein intake may have been different from the prescribed protein intake, due to noncompliance (compliance was 91% on average).

9 Some studies assessed multiple specific outcomes (i.e., multiple contrasts) for the health outcome “serum lipids,” so 1 study can show both a significant and a nonsignificant effect.

In 2 of the 7 RCTs (29%) a beneficial effect of increased protein intake on serum lipids was found for ≥1 of the statistically tested contrasts [2 of 43 contrasts (5%)], concerning total/HDL cholesterol ratio and triglycerides. A reducing effect on the total/HDL cholesterol ratio was observed in an RCT with 32 Brazilian participants that was performed in the context of physical exercise (51). A reducing effect on triglycerides was observed in an RCT of 34 Norwegian participants that was not performed in the context of physical exercise (63). One unfavorable effect was observed [1 of 43 contrasts (2%)] in an RCT of 22 US participants, where a 0.4-mmol/L greater decrease in LDL cholesterol was observed in the control group than in the intervention group after 12 wk (67). This RCT was not performed in the context of physical exercise. In the 3 RCTs showing an effect (either beneficial or unfavorable), the risk of bias was scored as “high.”

The committee judged that the evidence shows a high degree of ambiguity because of the opposite directions of the observed effects, the wide variety of lipid measures used, and the substantial difference in the proportion of beneficial effects based on the number of studies compared with the proportion of beneficial effects based on the number of contrasts.

#### Kidney function

The evaluation of the effect of increased protein intake on kidney function included 6 RCTs (45, 46, 56, 63–65), with a total of 12 statistically tested contrasts (Table 10; Supplemental Table 8). Assessed outcome measures include serum creatinine, estimated glomerular filtration rate (eGFR; estimated from serum creatinine), and albumin/creatinine ratio. The risk of bias was scored as “some concerns” (*n* = 4) or “high” (*n* = 2).

**TABLE 10:** Overview of the results of the 6 evaluated RCTs on the effect of increased protein intake on kidney function in older adults, categorized according to habitual protein intake and ordered by protein dose^1^

	Analytic *n* IG/CG	Total protein intake [g/(kg BW · d)] during intervention^2^	Protein dose^3^ [g/(kg BW · d)]	Protein type^4^	With/without physical exercise	Risk of bias^5^	Outcome measure	Result^6^				
Study								+	NS	−	?	Comments
Habitual protein intake (reference): ≥0.8 to <0.9 g/(kg BW · d)												
Ramel et al. 2013 (45)	237 (total)	IG: 1.06 ± 0.23; CG 0.89 ± 0.23	0.17	A	Ex	H	eGFR		✔			
Bhasin et al. 2018 (46)	40–46^£^/38–46^£^	IG: 1.17 ± 0.13; CG: 0.81 ± 0.10	0.36	A,B	NoEx	SC	Serum creatinine		✔			
* Subtotal (contrasts)*								*0*	*2*	*0*	*0*	*No effect observed for any of 2 contrasts*
*Subtotal (studies)*^7^								*0*	*2*	*0*	*0*	*No effect observed in either study*
Habitual protein intake (reference): ≥0.9 to <1.0 g/(kg BW · d)												
Ten Haaf et al. 2019 (65)	109–114^†^ (total)	IG: 0.92 ± 0.27 (without protein supplementation of 31 g/d); CG: 0.97 ± 0.23	0.36^8^	A	Ex	SC	Serum creatinine		✔			
							eGFR		✔			
							Albumin/creatinine ratio		✔			
Park et al. 2018 (64)	40/40	IG1: 1.18 ± 0.23; CG: 0.90 ± 0.38	0.28	A	NoEx	SC	Serum creatinine		✔			
							eGFR		✔			
	40/40	IG2: 1.37 ± 0.26; CG: 0.90 ± 0.38	0.47				Serum creatinine		✔			
							eGFR		✔			
Ottestad et al. 2017 (63)	17/18	IG: 1.4 ± 0.5; CG: 0.9 ± 0.4	0.5	B	NoEx	H	Serum creatinine	✔				
							eGFR		✔*			*P = 0.09 (eGFR tended to decrease less in IG than in CG)
* Subtotal (contrasts)*								*1*	*8*	*0*	*0*	*Beneficial effect observed for 1 of 9 contrasts*
*Subtotal (studies)*^7^								*1*	*3*	*0*	*0*	*Beneficial effect observed in 1 of 3 studies*
Habitual protein intake (reference): ≥1.0 to <1.1 g/(kg BW · d)												
Kerstetter et al. 2015 (56)	61/60 (18-mo follow-up)	IG: 1.30 ± 0.05; CG: 1.05 ± 0.04	0.25	A	NoEx	SC	eGFR		✔*			*No difference at 18 mo, but at 9 mo eGFR increased more in IG than in CG (P = 0.006)
*Subtotal (contrasts)*								*0*	*1*	*0*	*0*	*No effect observed for the single contrast*
* Subtotal (studies)* ^7^								*0*	*1*	*0*	*0*	*No effect observed in the single study*
*Total (contrasts)*								*1*	*11*	*0*	*0*	*Beneficial effect observed for 1 of 12 contrasts*
*Total (studies)*^7^								*1*	*6*	*0*	*0*	*Beneficial effect observed in 1 of 6 studies*

£ The exact number of participants included in the analyses is not reported. The number must be between the number of participants who were randomized and the number of participants who completed the study.

† Depending on the specific outcome measure.

§ Sufficient statistical power to detect an effect is to be expected, based on the sample size calculation.

1 BW, body weight; CG, control group; eGFR, estimated glomerular filtration rate; Ex, with concomitant exercise intervention; H, high risk of bias; IG, intervention group; L, low risk of bias; NoEx, without concomitant exercise intervention; NR, not reported; NS, not significant; SC, some concerns (regarding risk of bias); *, the result is accompanied by an explanation (see Comments).

2 Total protein intake during follow-up. If protein intake was assessed at multiple time points, the intake assessed at the final time point was considered.

3 ”Protein dose” indicates the difference in achieved total protein intake between the intervention group and the control group during follow-up (which is not necessarily equal to supplemented/prescribed amount of protein).

4 ”Protein type” indicates the way in which a higher protein intake was achieved and is categorized into protein or amino acid supplements (A), 1 or a few protein-(en)rich(ed) foods (B), or high-protein diets (C).

5 Risk of bias was assessed using the RoB 2 Cochrane collaboration tool and scored as “low” (L), “some concerns” (SC) or “high” (H).

6 The results of the studies are indicated as follows: +, statistically significant beneficial effect (*P* < 0.05); −, statistically significant unfavorable effect (*P* < 0.05); NS, no statistically significant effect (*P* ≥ 0.05); ?, result unclear. In cases where results were reported for multiple time points, only the result for the final time point is reported.

7 Some studies assessed multiple specific outcomes (i.e., multiple contrasts) for the health outcome “kidney function,” so 1 study can show both a significant and a nonsignificant effect.

8 Protein intake in g/(kg BW · d) was calculated by using protein intake in g/d, mean BW, and compliance.

In 1 of the 6 RCTs (17%) an effect of increased protein intake on kidney function was found for 1 of the statistically tested contrasts [1 of 12 contrasts (8%)], concerning serum creatinine (63). In this RCT including 35 participants from Norway, a greater increase in serum creatinine level was observed in the protein group compared with the control group after 12 wk. The risk of bias in this RCT was scored as “high.” No other effects on kidney function were observed.

Of note, a number of RCTs into the effect of increased protein intake assessed (parameters of) kidney function, but not as primary outcome. Kidney function was predominantly evaluated to identify a possible adverse effect of increased protein intake. Whether the study samples in those studies were large enough to detect any (adverse) effect is uncertain. The committee furthermore noted that high-risk groups for deteriorating kidney function (e.g., those with diabetes, hypertension, or pre-existing impaired kidney function) were often excluded from RCTs. Protein may have a different effect in people in those high-risk groups compared with people with good kidney function. Hence, the generalizability of the findings reported here to those in high-risk groups might be limited.

The committee noted serious limitations when using serum creatinine or eGFR based on serum creatinine (68) as an indicator of kidney function in the context of protein RCTs, which most evaluated RCTs did. Many factors, including the amount of protein, the protein source (animal- or plant-based), and any change in muscle mass, can affect the level of serum creatinine (69). These effects may occur simultaneously and in opposing or similar directions, thereby respectively potentially neutralizing the effects of one another or leading to a seemingly changing eGFR without actually changing the true GFR. Because of this complexity, the committee believes that serum creatinine and the creatinine-based eGFR are inappropriate measures to determine the isolated effect of dietary protein intake on kidney function. Hence, the available data are insufficient to exclude an adverse effect of a long-term increase of protein intake on kidney function in older adults.

#### Cognition

The evaluation of the effect of increased protein intake on cognition included 1 RCT (64), in which 2 contrasts were statistically tested (Table 11; Supplemental Table 9). Cognition was assessed using the Mini-Mental State Examination (MMSE). The risk of bias was scored as “some concerns.” This RCT showed no effect of increased protein intake on cognition.

**TABLE 11:** Overview of the results of the evaluated RCT on the effect of increased protein intake on cognition in older adults, ordered by protein dose^1^

	Analytic *n* IG/CG	Total protein intake [g/(kg BW · d)] during intervention^2^	Protein dose^3^ [g/(kg BW · d)]	Protein type^4^	With/without physical exercise	Risk of bias^5^	Outcome measure	Result^6^				
Study								+	NS	−	?	Comments
Habitual protein intake (reference): ≥0.9 to <1.0 g/(kg BW · d)												
Park et al. 2018 (64)	40/40	IG1: 1.18 ± 0.23; CG: 0.90 ± 0.38	0.28	A	NoEx	SC	Korean MMSE		✔			
	40/40	IG2: 1.37 ± 0.26; CG: 0.90 ± 0.38	0.47				Korean MMSE		✔			
*Total (contrasts)*								*0*	*2*	*0*	*0*	*No effect observed for any of 2 contrasts*
*Total (studies)*^7^								*0*	*1*	*0*	*0*	*No effect observed in the single study*

^1^BW, body weight; CG, control group; IG, intervention group; MMSE, Mini-Mental State Examination; NoEx, without concomitant exercise intervention; NS, not significant; SC, some concerns (regarding risk of bias).

2 Total protein intake during follow-up. If protein intake was assessed at multiple time points, the intake assessed at the final time point was considered.

3 ”Protein dose” indicates the difference in achieved total protein intake between the intervention group and the control group during follow-up (which is not necessarily equal to supplemented/prescribed amount of protein).

4 ”Protein type” indicates the way in which a higher protein intake was achieved and is categorized into protein or amino acid supplements (A), 1 or a few protein-(en)rich(ed) foods (B), or high-protein diets (C).

5 Risk of bias was assessed using the RoB 2 Cochrane collaboration tool and scored as “low” (L), “some concerns” (SC) or “high” (H).

6 The results of the studies are indicated as follows: +, statistically significant beneficial effect (*P* < 0.05); −, statistically significant unfavorable effect (*P* < 0.05); NS, no statistically significant effect (*P* ≥ 0.05); ?, result unclear. In cases where results were reported for multiple time points, only the result for the final time point is reported.

7 This study assessed multiple contrasts for the health outcome “cognition,” so could show both a significant and a nonsignificant effect.

#### Subgroup analysis according to concomitant physical exercise

The committee evaluated whether the effect of increased protein intake on health outcomes differed according to whether or not the protein intervention took place in the context of a (concomitant) physical exercise intervention for the following health outcomes: lean body mass, muscle strength, physical function, and serum lipids (Tables 3–5 and 9; Supplemental Tables 1–3 and 7; **Supplemental Results 1**). For the outcomes of lean body mass, physical function, and serum lipids, there were no notable differences between results obtained by RCTs on the effect of increased protein intake alone (compared with placebo) and those obtained by RCTs on the effect of increased protein intake with concomitant physical exercise (compared with physical exercise only). For muscle strength, RCTs on the effect of increased protein intake in the context of physical exercise more often showed a beneficial effect on muscle strength (38% of 8 RCTs evaluated; 24% of 55 contrasts tested) than RCTs on the effect of increased protein intake alone (14% of 7 RCTs evaluated; 4% of 28 contrasts tested). Physical exercise usually concerned resistance training. For the outcomes of blood pressure, serum glucose and insulin, and kidney function, RCTs with and without concomitant physical exercise were available, but results were not stratified since (nearly) all RCTs for the given health outcome showed the same result (i.e.,*likely no effect*). For the outcomes of bone health and cognition, results could not be stratified since an insufficient number of studies was represented in each group.

#### Subgroup analysis according to habitual protein intake

The committee evaluated whether the effect of increased protein intake on health outcomes differed according to domain of habitual protein intake for 4 health outcomes: lean body mass, muscle strength, physical function, and serum lipids. For those 4 outcomes, the percentage of RCTs showing an effect compared with the percentage of RCTs showing no effect was not notably different across domains of habitual protein intake (Tables 3–5 and 9; **Supplemental Results 2**). For the outcomes of bone health, blood pressure, serum glucose and insulin, and kidney function, RCTs in multiple domains of habitual protein intake were available, but results were not stratified since (nearly) all RCTs for the given health outcome showed the same result (i.e.,*likely no effect*). For cognition, results could not be stratified since only 1 RCT was available.

#### Sensitivity analyses

For none of the health outcomes evaluated did the committee find evidence of a dose–response relation (Tables 3–11; **Supplemental Results 2**). The committee also found no indications that the observed results differed according to the type of protein intervention or the level of risk of bias. The lack of information concerning the power calculations in more than one-third of the selected RCTs made it difficult to determine the extent to which statistical power influenced the results obtained.

### Applying decision rules

Based on the overall effects observed in the 18 RCTs included in this SR, as well as subgroup evaluations, sensitivity analyses, and other considerations, the committee drew final conclusions regarding the effect on health outcomes of increasing protein intake in older adults with a habitual protein intake ≥0.8 g/(kg BW ⋅ d). The committee thereby used the prespecified decision rules (Table 1) as a starting point. The final conclusions are as follows. There is a *possible beneficial effect* of increased protein intake on lean body mass in older adults, which does not involve any change in BW. There is also a *possible beneficial effect* on muscle strength, but only for the combination of increased protein intake and concomitant physical exercise (mainly resistance exercise training) compared with physical exercise alone. Increased protein intake alone (not in the context of physical exercise) has *likely no effect*on muscle strength. There is also *likely no effect* of increased protein intake on physical performance and bone health. Effects of increased protein intake on blood pressure, serum glucose and insulin, kidney function, and cognition are unclear because *too few studies* were available (cognition), or *too few studies* with sufficient statistical power (blood pressure, serum glucose, and insulin) or appropriate outcome measures (kidney function) were available to draw conclusions. There is an *ambiguous effect* of increased protein intake on serum lipids. Based on the data available, the committee judged that there were no indications that the results obtained would differ between older adults who engage in physical exercise and those who do not (except for muscle strength), or would depend on the habitual protein intake. In addition, the type of protein intervention, risk of bias, or statistical power did not seem to influence the results obtained. Last, there were no indications for a dose–response relation (exploratory analysis).

## Discussion

The present SR provides a comprehensive, transparent, and up-to-date overview of peer-reviewed human RCTs that investigated the effects of increased protein intake on health outcomes in older adults from the general population with an average habitual protein intake of at least 0.8 g/(kg BW ⋅ d). The study showed that increasing protein intake above 0.8 g/(kg BW ⋅ d) has a*possible beneficial effect* on lean body mass and, only for the combination with physical exercise, muscle strength. However, an effect of protein intake when not accompanied by physical exercise on muscle strength, or on physical function and bone health was *unlikely*. For 5 other health outcomes assessed, effects of extra protein intake are unclear. Taken together, those RCTs did not provide convincing evidence that a protein intake beyond 0.8 g/(kg BW ⋅ d) affects health outcomes in older adults.

The present SR of RCTs has been used by the Committee on Nutrition of the Health Council of the Netherlands to set the PRI for protein for older adults, in addition to an MA of nitrogen-balance studies, which was used as the starting point for this derivation. The MA, performed by Rand et al. (10), showed that the average requirement of high-quality protein for adults aged >18 y was 0.66 g/(kg BW ⋅ d), which resulted in a PRI of 0.83 g/(kg BW ⋅ d). The average nitrogen requirement was higher in a small subgroup of older adults (>67 y; *n* = 14 from 1 RCT) compared with a subgroup of younger adults (<40 y; *n* = 221), but the difference was not statistically significant. This was confirmed by a nitrogen-balance study comparing adults younger with those older than 60 y published thereafter (70). No other, more recent, nitrogen-balance studies were found. Alternative methods such as stable isotope studies do not yet provide enough robust data to base PRI for proteins on. The committee secondarily considered the results of the present SR. The data suggest that increasing protein intake in older adults with a habitual protein intake of at least 0.8 g/(kg BW ⋅ d) has a *possible beneficial effect* on lean body mass and muscle strength (for the combination with physical exercise only), which may be of importance for metabolic and physical health. Still, the majority (over 60%) of the RCTs evaluated showed no effect on (≥1 measure of) those outcomes, nor did it seem to translate into better physical function or to affect bone health. Moreover, effects of extra protein intake on the 5 other health outcomes assessed are unclear (due to an insufficient number of studies available, an assumed lack of statistical power, inappropriateness of outcome measures used, or ambiguous findings). Altogether, the committee judged that the currently available RCTs do not provide sufficiently convincing evidence that a protein intake beyond 0.8 g/(kg BW ⋅ d), compared with ≥0.8 g/(kg BW ⋅ d), affects health outcomes in older adults. Hence, the committee concluded that no higher PRI for (high-quality) protein was needed for (healthy) older adults than for (healthy) younger adults (71).

DRVs are applicable to the general (healthy) population. The committee, therefore, disregarded studies performed among hospitalized patients or specific (older) patient groups such as those with chronic heart failure or chronic obstructive pulmonary disease. The committee does not rule out the possibility that certain subgroups of older adults, such as frail or malnourished older adults, may indeed benefit from more protein than the PRI for older adults in general (5). This question fell outside the scope of this SR and could not be answered based on the literature available in this SR.

For the outcomes of lean body mass and muscle strength (only for the effect of protein in the context of physical exercise) the committee concluded that a beneficial effect of increased protein intake was p*ossible*. Evidence was too limited to allow a conclusion of *likely*or *convincing* due to inconsistency in study findings. As stated before, the inconsistency was likely not explained by the protein dose, the presence of concomitant physical exercise (except for muscle strength), the type of protein intervention, and the risk of bias. Also, the discrepancy in findings for lean body mass was unlikely due to differences in age, gender, or BMI. The committee noted that among the 9 RCTs in the context of physical exercise, (beneficial) effects were observed in 3 RCTs, which had a few things in common that differed from the other 6 RCTs: participants were all female, were all from Brazil, the (high) dose of protein was ingested directly after the training session (instead of during a meal and/or spread over the day), and participants received training 8 wk before the start of the trial. It could not be determined which of those factor(s) explained the positive findings. For muscle strength, the disparity in results could be partially explained by the presence of concomitant physical exercise. However, inconsistency was still present among the RCTs that were performed in the context of physical exercise. Differences in age, gender, BMI, nationality, race, timing of protein intake, or baseline mobility status did likely not explain the inconsistent findings.

The fact that the literature selection and risk of bias assessment in this SR were not (formally) performed in duplicate can be considered a limitation. However, those tasks were performed by 1 researcher in close collaboration with and (quality) control by the full committee, among whom were multiple experts active in the field of protein and aging research. Therefore, we are confident that no (influential) studies were missed and that the likelihood of (substantial) errors in the risk of bias assessment is small. A second limitation is that the literature search was performed in April 2020, and thus, it cannot be ruled out that any very recently published studies regarding the effect of protein intake on health outcomes in older adults have not been included in the present SR.

An important limitation to many published (SRs of) RCTs into increasing protein intake in older adults in general is the lack of information on the participants’ habitual protein intake. Particularly when such studies serve to derive DRVs the interest lies in the total protein intake rather than the protein dose only. In addition, the effect of extra protein may depend on the habitual protein intake and this may also be an explanation for the heterogeneity observed among previous studies. The present SR addressed this issue; it is unique in that it specifically focused on RCTs in which the participants’ habitual protein intake was at least 0.8 g/(kg BW ⋅ d). It is recommended that future studies showing beneficial effects stratify their results by habitual protein intake, in order to provide better insight into the intake domain in which health benefits occur. All available RCTs except 1 (66) used a dairy-based protein intervention (either dairy-based milk, milk protein concentrate, or whey protein concentrate) or a supplement of essential amino acids. Therefore, the committee could not evaluate whether effects of protein on health outcomes depend on the dietary protein source (animal- compared with plant-based). Future research on this topic is important, particularly given the environmental challenges the world is facing today. More RCTs with sufficient statistical power for detecting effects are also needed, especially with regard to cardiometabolic outcomes such as blood pressure and serum glucose and insulin. Furthermore, the protein requirement might be different (higher) in the oldest old (≥85 y), but the committee found no studies that were conducted in this age group. Therefore, studies performed in the oldest old are required. Regarding prospective cohort studies, the committee noted the importance of studies addressing more, and more specifically for the DRV relevant, categories of protein intake in relation to health outcomes. This would contribute to better specifying the optimal protein intake level. Lastly, there is a need for more nitrogen-balance studies in which both younger adults and older adults are represented in order to better study any age differences in protein metabolism.

## Conclusion

The results from the present SR of RCTs indicate that increasing protein intake beyond 0.8 g/(kg BW ⋅ d) has a*possible beneficial effect* on lean body mass in older adults and, when combined with physical exercise, muscle strength, but that an effect on physical performance and bone health is *unlikely*. Limitations with regard to sample size, statistical power, and appropriateness of outcome measures did not allow for conclusions regarding other health outcomes, such as blood pressure, or to rule out potential harmful effects of extra protein, for example on kidney function. The committee judged that the available evidence from human RCTs is not sufficiently convincing to state that increasing protein intake in older adults from the general population with a habitual protein intake of at least 0.8 g/(kg BW ⋅ d) would elicit health benefits.

## Supplementary Material

nmab140_Supplemental_File

## Notes

This systematic review has been adapted from a background document to an advisory report aimed to substantiate Dutch public health policy. The report and systematic review have been funded by the Health Council of the Netherlands, an independent scientific advisory body whose legal task it is to advise ministers and Parliament in the field of public health/healthcare research. LMH and JdG are employees and JMG is a member of the Board of the Health Council of the Netherlands. LAA, SJLB, JWJB, EEB, HB, JBvG, MTEH, JAI, SPJK, RPM, NMdR, CDAS, EdV, and MV are members of the Council's Permanent Committee on Nutrition and received financial compensation for meeting attendance and travelling expenses from the Council. JV-K was involved as a structurally consulted expert to the Council's Permanent Committee on Nutrition. All experts who contribute to the work of the Health Council fill in a declaration of interests. This declaration is part of the Code for the prevention of improper influence due to conflicts of interests. He or she also provides information about research funds received and any personal financial interest. Based on this, the Board of the Health Council of the Netherlands decides whether or not an expert can participate in the committee. In case of direct personal financial interests in relation to nutrition topics, participation is not possible. The declarations of interest are periodically updated and are publicly available on the website (www.gezondheidsraad.nl).

Author disclosures: SJLB has received a research grant from FrieslandCampina for research into biomarkers of dairy intake. JBvG has 2 patents related to the amino acid composition of nutrition for premature born infants until the age of 6 mo. MTEH has received research grants from FrieslandCampina for research into muscle damage markers and soreness following a 15-km road race and from New Care for research into vegetable protein intake compared with animal protein intake in relation to muscle pain and muscle repair after endurance sport. MV is PI of the PRevention Of Malnutriton In Senior Subjects (PROMISS) project, funded by the European Committee's Horizon2020 program, which has a focus on the role of protein intake in the prevention of malnutrition and in physical functioning. All other authors report no conflicts of interest.

Supplemental Methods 1–3, Supplemental Tables 1–13 and Supplemental Results 1–2 are available from the “Supplementary data” link in the online posting of the article and from the same link in the online table of contents at https://academic.oup.com/advances/.

Abbreviations used: BW, body weight; CG, control group; CTX, C-terminal telopeptide of type 1 collagen; DRV, dietary reference value; EFSA, European Food Safety Authority; eGFR, estimated glomerular filtration rate; IG, intervention group; LST, lean soft tissue; P1NP, N-terminal propeptides of type 1 procollagen; PRI, population reference intake; RM, repetition maximum; RoB 2, revised Cochrane risk of bias tool for randomized trials; SPPB, Short Physical Performance Battery; SR, systematic review; TUG, Timed Up and Go.
