# Supplementary material for: Health Effects of Increasing Protein Intake Above the Current Population Reference Intake in Older Adults: A Systematic Review of the Health Council of the Netherlands
Source: Adv Nutr. 2021 Nov 23;13(4):1083–117. doi: 10.1093/advances/nmab140 (PMC9340973; doi:10.1093/advances/nmab140)
Supplement: nmab140_Supplemental_File [file nmab140_supplemental_file.docx]

**Online Supplemental Material**

**Health effects of increasing protein above the current population reference intake in older adults: a systematic review of the Health Council of the Netherlands**

L.M. Hengeveld, J. de Goede, L.A. Afman, S.J.L. Bakker, J.W.J. Beulens, E.E. Blaak, H. Boersma, J.M. Geleijnse, J.B. van Goudoever, M.T.E. Hopman, J.A. Iestra, S.P.J. Kremers, R.P. Mensink, N.M. de Roos, C.D.A. Stehouwer, J. Verkaik-Kloosterman, E. de Vet, M. Visser

Contents

[Supplemental Methods 1 3](#_Toc84515516)

[Supplemental Methods 2 5](#_Toc84515517)

[Supplemental Methods 3 6](#_Toc84515518)

[Supplemental Table 1 8](#_Toc84515519)

[Supplemental Table 2 25](#_Toc84515520)

[Supplemental Table 3 42](#_Toc84515521)

[Supplemental Table 4 51](#_Toc84515522)

[Supplemental Table 5 57](#_Toc84515523)

[Supplemental Table 6 60](#_Toc84515524)

[Supplemental Table 7 65](#_Toc84515525)

[Supplemental Table 8 72](#_Toc84515526)

[Supplemental Table 9 77](#_Toc84515527)

[Supplemental Table 10 79](#_Toc84515528)

[Supplemental Table 11 86](#_Toc84515529)

[Supplemental Table 12 87](#_Toc84515530)

[Supplemental Table 13 89](#_Toc84515531)

[Supplemental Results 1 94](#_Toc84515532)

[Supplemental Results 2 95](#_Toc84515533)

[References 99](#_Toc84515534)

Supplemental Methods 1

Search strategies for literature search in PubMed and Scopus

Search for systematic reviews

Search strategy used by the council’s committee to identify systematic reviews (including meta-analyses and individual participant data analyses) of randomized controlled trials and prospective cohort studies on the relationship between protein intake and health outcomes in older adults. The literature search was conducted in PubMed on 23 April 2020.

*PubMed*

(Dietary Proteins[MeSH] OR dietary protein*[tiab] OR protein intake[tiab] OR protein supplement*[tiab] OR nutritional protein*[tiab] OR dietary protein*[tiab] OR Amino Acids, Essential[MeSH] OR amino acid* OR casein[tiab] OR whey*[tiab] OR nutritional requirements[MeSH] OR nutritional requirement*[tiab] OR milk protein* OR (protein*[tiab] AND supplement*[tiab])) AND (Aged[MeSH] OR aged[tiab] OR Middle Aged[MeSH] OR middle aged[tiab] OR elderly[tiab] OR old* people*[tiab] OR old* person*[tiab] OR old* adult*[tiab] OR old* population*[tiab] OR healthy older adults[tiab]) AND ((Systematic review [publication type] OR "Systematic Reviews as Topic"[Mesh] OR systematic review [tiab]) OR (meta-analysis [publication type] OR "Meta-Analysis as Topic"[Mesh] OR meta-analysis [tiab]) OR (individual participant data [tiab]))

No filters applied.

Search for recent individual randomized controlled trials

Search strategy used by the council’s committee to identify recent individual randomized controlled trials on the effect of increased protein intake on health outcomes in older adults. The literature search was conducted in PubMed and Scopus on 23 April 2020.

*PubMed*

(Dietary Proteins[MeSH] OR dietary protein*[tiab] OR protein intake[tiab] OR protein supplement*[tiab] OR nutritional protein*[tiab] OR dietary protein*[tiab] OR Amino Acids, Essential[MeSH] OR amino acid* OR casein[tiab] OR whey*[tiab] OR nutritional requirements[MeSH] OR nutritional requirement*[tiab] OR milk protein* OR (protein*[tiab] AND supplement*[tiab])) AND (Aged[MeSH] OR aged[tiab] OR Middle Aged[MeSH] OR middle aged[tiab] OR elderly[tiab] OR old* people[tiab] OR old* person*[tiab] OR old* adult*[tiab] OR old* population*[tiab] OR healthy older adults[tiab]) AND (clinical trial[publication type] OR "Clinical Trials as Topic"[Mesh] OR clinical trial[tiab])

Filters: limited to publication year 2018, 2019, 2020

*Scopus*

(TITLE-ABS-KEY(Dietary-Protein*) OR TITLE-ABS-KEY(protein-intake) OR TITLE-ABS-KEY(protein-supplement*) OR TITLE-ABS-KEY(protein-source*) OR TITLE-ABS-KEY(source-of-protein) OR TITLE-ABS-KEY(nutritional-protein*) OR TITLE-ABS-KEY(dietary-protein*) OR TITLE-ABS-KEY(Amino-Acids*) OR TITLE-ABS-KEY(casein) OR TITLE-ABS-KEY(whey*) OR TITLE-ABS-KEY(Milk proteins) OR TITLE-ABS-KEY(nutritional- requirement*) OR TITLE-ABS-KEY(Protein) AND TITLE-ABS-KEY(supplement)) AND (TITLE-ABS-KEY(aged) OR TITLE-ABS-KEY(elderly) OR TITLE-ABS-KEY(old*-people) OR TITLE-ABS-KEY(old*-person*) OR TITLE-ABS-KEY(old*-population*) OR TITLE-ABS-KEY(old*-adult*) OR TITLE-ABS-KEY(healthy-older-adults) OR TITLE-ABS-KEY(middle aged)) AND TITLE-ABS-KEY(clinical-trial)

Filters: limited to publication year 2018, 2019, 2020

Supplemental Methods 2

Inclusion and exclusion criteria for selection of studies

Inclusion criteria

1. Randomized controlled trials;
2. Minimal intervention duration of 4 weeks;
3. Sampling age of ≥50 and(/or) a mean age of ≥65;
4. Community-dwelling older adults or older adults living in a nursing home or care home;
5. Participants with a minimal (habitual) protein intake of 0.8 g/kg BW/d.

Exclusion criteria

1. Study population comprising people with a specific (chronic) disease or condition, such as cancer, diabetes, chronic obstructive pulmonary disease, heart failure, polymyalgia rheumatic or osteoporosis;
2. Hospitalised patients;
3. Immobilised patients;
4. Studies conducted directly prior or subsequent to surgery or hospitalisation (e.g. pre- or postoperative studies or studies after hospital discharge);
5. Exposure is individual amino acids (e.g. leucine), combinations of only two or three amino acids (e.g. branched-chain amino acids (BCAAs^[[1]](#footnote-1)^)), or dipeptides (e.g. beta-alanine or carnosine);
6. Exposure is not protein, amino acids or protein-(en)rich(ed) food products (e.g. creatine, dehydro-epiandrosteron (DHEA), beta-hydroxy-beta-methylbutyrate (HMB), and milk fat globule membrane (MFGM) are excluded);
7. Outcome is muscle protein synthesis;
8. No isocaloric control intervention;
9. The intervention group and control group differ (intentionally) in more ways than the amount of protein involved, i.e., studies with a hard^[[2]](#footnote-2)^ contrast were excluded;
10. The intervention is part of a weight-loss programme (e.g. energy-restricted diet).

Supplemental Methods 3

Sensitivity analyses

Factors that might attribute to heterogeneity in observed results

The committee evaluated, as a sensitivity analyses, whether certain factors might have been potential sources of the heterogeneity in observed results across studies. Therefore, it checked whether there were indications that the observed effects differed according to categories or level of the following factors: type of protein intervention, risk of bias and if the study was statistically powered for the given outcome measure or not. Details of this evaluation are described below.

*Type of protein intervention*

Participants can be exposed to protein in a various ways; for example, through a complete high-protein diet, a few protein-rich foods or an amino acid supplement. Due to varying food matrices, those types of interventions may differentially affect health outcomes. The committee, therefore, distinguished between three types of protein interventions:

1. Protein or amino acid supplements – These are generally provided in the form of a powder or pill, such as whey hydrolysates, whey isolates, whey concentrates or milk-protein concentrates, and dissolved in a (low-protein) drink;
2. One or a few protein-(en)riched foods – These include protein-enriched products, such as commercially-available protein-enriched milk, or ‘ordinary’ protein-rich foods, such as cow milk or soy milk. Consumption of these products is usually associated with consumption of more nutrients than protein alone;
3. High-protein diets – With this type of intervention, the entire dietary pattern is modified to achieve a pre-specified total protein intake (e.g. 0.8 g/kg BW/d) in the control group and an increased total protein intake (e.g. 1.2 g/kg BW/d) in the intervention group. With such a complete diet, not only the amount of protein but also the amount of other nutrients is generally modified (albeit unintentionally, as it is inevitable with food-based interventions).

*Risk of bias*

The (overall) risk of bias of each RCT was judged and scored as ‘low risk of bias’, ‘some concerns’ or ‘high risk of bias’. The committee evaluated whether there were indications that the observed effects differed according to the level of risk of bias, i.e. if the studies with a high risk of bias showed an effect more often (or less often) than the studies with ‘some concerns regarding the risk of bias’ or with a low risk of bias.

*Statistical power*

Insufficient statistical power may be one of the reasons for studies not showing an effect of increased protein intake on a given health outcome. If this is the case, this may result in an over-representation of studies with neutral findings (i.e. no effect). For this reason, as a sensitivity analysis, the committee restricted itself to the studies where the power analysis was based on that outcome measure. Unless indicated otherwise in the individual studies, the committee has made two assumptions in this regard. Firstly, the committee assumed that, within an RCT, the calculated sample size is the same for all specific outcome measures within the domain of that health outcome. For example, if the power analysis was based on handgrip strength (a specific outcome measure within the domain ‘muscle strength’), it is likely that the sample size derived in this way will also apply to other specific outcome measures within the domain ‘muscle strength’, such as knee extensor strength or leg press. Secondly, the committee assumed (based on expert judgement) that for demonstrating a statistically significant effect, lean body mass generally requires a smaller sample size than muscle strength, and that muscle strength requires a smaller sample size than physical performance. This amounts to the assumption that a study in which the power analysis was based on muscle strength will also have sufficient statistical power for lean body mass. Similarly, a study in which the power analysis was based on physical performance will also have sufficient statistical power for lean body mass and muscle strength. No assumptions have been made for the other outcomes. The committee used the available information on this topic, as derived from the studies. It did not perform its own power analyses (post-intervention) to determine whether or not the sample size was (in retrospect) sufficient for a particular outcome.

Indication of a dose-response relationship

The committee evaluated, as a sensitivity analysis, whether there was an indication of a dose-response relationship across RCTs, i.e. whether an effect was more often observed or whether a stronger effect was observed in studies in which the protein dose was higher. For this purpose, the committee checked, within each domain of habitual protein intake, whether the protein dose in the studies showing an effect was higher than the protein dose in the studies showing no effect.

Supplemental Table 1

Results from randomized controlled trials on the effect of increased protein intake on lean body mass in older adults, grouped according to whether or not the protein intervention was carried out in the context of (concomitant) physical exercise

| Study, country | Study population | Exposure (IG), [protein type^a^] | Comparison (CG) | Compliance | Total protein intake (g/kg BW/d) | Study duration | Risk of bias^b^ | Outcome | Analytic *n* IG/CG | Results | |
| --- | --- | --- | --- | --- | --- | --- | --- | --- | --- | --- | --- |
| Not in the context of (concomitant) physical exercise | | | | | | | | | | |  |
| Bhasin et al. 2018 (1), USA | Community-dwelling older men aged ≥65 y with moderate physical function limitations and with habitual protein intake ≤0.83 g/kg BW/d; mean age: 73 ± 6 y; mean BMI: 30.3 ± 4.9 kg/m^2^; race NR | Individualised diets providing 0.7 g protein/kg BW/d with additional discretionary foods (0.1 g protein/kg BW) and protein supplements (0.5 g/kg BW; 50:50 casein:whey mix) to achieve a total of 1.3 g/kg BW/d, [A,B] | Individualised diets providing 0.7 g protein/kg BW/d with additional discretionary foods (0.1 g protein/kg BW) and placebo supplements (0.5 g CHO/kg BW) to achieve a total of 0.8 g/kg BW/d | Foods (4-6 mo):  IG: 77.1 ± 13%, CG: 74.5 ± 23.2%  Supplements (4-6 mo):  IG: 91.2 ± 12.4%, CG: 92.6 ± 11.0% | Baseline:  IG: 0.72 ± 0.11, CG: 0.69 ± 0.15  Follow-up (1-3 mo):  IG: 1.18 ± 0.15, CG: 0.84 ± 0.07  Follow-up (4-6 mo): IG: 1.17 ± 0.13, CG: 0.81 ± 0.10 | 6 mo | Some concerns | Total LBM^§^ (kg; DXA) | 42/39  (mITT) | MD (95%-CI):  +0.31 (-0.46 to +1.08)  P=0.43  For relative total LBM (% of BW), MD (95%-CI): +0.81 (+0.05 to +1.58), P=0.04. Mainly a result of the significantly greater decrease in total fat mass (kg) in IG than in CG (P=0.02). | |
|  |  |  |  |  |  |  |  | Trunk LBM^§^ (kg; DXA) | 42/39  (mITT) | MD (95%-CI):  +0.24 (-0.17 to +0.66)  P=0.24 | |
|  |  |  |  |  |  |  |  | aLBM^§^ (kg; DXA) | 42/39  (mITT) | MD (95%-CI):  +0.04 (-0.48 to +0.55)  P=0.89 | |
| Dillon et al. 2009 (2), USA | Community-dwelling older women; without vascular disease, hypertension or cardiac abnormality; mean age: 68 ± 2 y; mean BMI NR; race NR | EAA. 7.5 g EAA, ingested twice a day in between meals (total: 15 g EAA/d), [A] | CHO placebo. Isocaloric amount of lactose, ingested twice a day in between meals | NR | Baseline:  NR  Follow-up: NR | 3 mo | High | Total LBM (kg; DXA) | 7/7 | Authors reported no results for time*group interaction (ANOVA), which suggests that protein has no effect | |
| Ispoglou et al. 2016 (3), UK | Community-dwelling older men and women aged 65-75 y; good health, without major chronic diseases (e.g. diabetes, vascular disease, hypertension); 44% female; mean age: 72 ± 3 y; mean BMI: 27 ± 4 y; race: NR | EAA. Standard EAA mixture with 20% leucine (IG1) and with 40% leucine (IG2), ingested at breakfast and dinner (total: 0.21 g/kg BW/d = ~11-21 g/d), [A] | CHO placebo. Isocaloric amount of lactose, ingested at breakfast and dinner | 74-83% | Baseline: 0.95-1.10  Follow-up: 1.02-1.08 (without supplementation) | 12 wk | High | Total LTM (kg; DXA) | 8/8/9  (PP) | Mean % change ± SD:  IG1: +0.2 ± 2.4  IG2: +1.1 ± 1.1  CG: +0.8 ± 1.3  Time*group interaction NS | |
| Kerstetter et al. 2015 (4), USA | Older men (aged >70 y) and women (aged >60 y) with BMI of 19-32 kg/m^2^ and protein intake of 0.6-1.0 g/kg BW; without major chronic diseases (e.g. diabetes, renal disease, inflammatory bowel disease) or cancer within past 18 months; 86% female; mean age: 70 ± 6 y; mean BMI: 26 ± 4 kg/m^2^; Caucasian | Whey protein. 45 g of whey protein isolate (~40 g of protein) + vitamin D (400 IU) + calcium (1200 mg), [A] | CHO placebo.  Isocaloric amount of maltodextrin + vitamin D (400 IU) + calcium (1200 mg) | NR | Baseline:  IG: 1.07 ± 0.03, CG: 1.06 ± 0.03  Follow-up:  IG: 1.30 ± 0.05, CG: 1.05 ± 0.04 | 18 mo | Some concerns | Total LBM (kg; DXA) | 105/102  (mITT) | LSMD (95%-CI): -0.52 (-1.08 to 0.04)  P=0.069  (No significant change in total fat mass (P>0.05)) | |
|  |  |  |  |  |  |  |  | Trunk LBM (kg; DXA) | 105/102  (mITT) | LSMD (95%-CI):  -0.33 (-0.660 to -0.003)  **P=0.048**  (trunk LBM decreased less in IG than in CG) | |
| Mitchell 2017 (5), New Zealand | Community-dwelling older men aged >70 y with BMI of 18-35 kg/m^2^; able to perform ADLs without mobility aids; without major chronic diseases (e.g. cancer, diabetes, thyroid diseases); mean age: 74 y (range: 70-81 y); mean BMI: 28 ± 4 kg/m^2^; race NR | High-protein diet (2*RDA = 1.6 g/kg BW/d), omnivorous, 28-31 E% fat, [C] | Low-protein diet (1*RDA = 0.8 g/kg BW/d), omnivorous, 28-31 E% fat; difference made up of CHO | IG: 97.5%, CG: 98.9% | Baseline:  IG: 1.1 ± 0.3, CG: 1.2 ± 0.4  Follow-up:  IG: 1.7 ± 0.1, CG: 0.9 ± 0.1 | 10 wk | High | Total LBM^§^ (kg; DXA) | 14/15  (PP) | Mean change ± SD:  IG: +1.49 ± 1.30 CG: -0.55 ± 1.49  **P for time*group interaction=0.001**  (No significant change in body weight (P=0.174), but greater decrease in total and % fat mass in IG than in CG (both P<0.01)) | |
|  |  |  |  |  |  |  |  | Trunk LBM^§^ (kg; DXA) | 14/15  (PP) | **P for time*group interaction<0.001** | |
|  |  |  |  |  |  |  |  | aLBM^§^ (kg; DXA) | 14/15  (PP) | **P for time*group interaction=0.022** | |
|  |  |  |  |  |  |  |  | Muscle CSA, thigh^§^ (mm2; QCT) | 14/15 (PP) | Mean change ± SD:  IG: -120 ± 292 CG: -539 ± 786 P for time*group interaction=0.112 | |
| Ottestad et al. 2017 (6), Norway | Community-dwelling older adults aged ≥70 y; relatively healthy (no diabetes, CVD, cancer, COPD, CKD); not malnourished; with reduced muscle strength or performance; 67% female; mean age: 77 ± 5 y; mean BMI: 27 ± 5 kg/m^2^; race NR | Protein-enriched milk. 400 ml drink containing 20 g protein (80:20 casein:whey mix, consumed twice a day (total: 40 g protein/d), [B] | CHO placebo. 400 ml drink containing an isocaloric amount of CHO, consumed twice a day | IG: 97.8 ± 3.8%, CG: 96.8 ± 5.7% | Baseline:  IG: 1.0 ± 0.3, CG: 1.0 ± 0.3  Follow-up:  IG: 1.4 ± 0.5, CG: 0.9 ± 0.4 | 12 wk | High | Total LBM (kg; DXA) | 17/19 (mITT) | Mean change (95%-CI):  IG: +0.4 (0.0 to +0.8)  CG: +0.4 (0.0 to +0.9)  P=0.85 | |
|  |  |  |  |  |  |  |  | Trunk LBM (kg; DXA) | 17/19  (mITT) | Mean change (95%-CI):  IG: +0.2 (0.0 to +0.5)  CG: 0.0 (-0.3 to +0.4)  P=0.33 | |
|  |  |  |  |  |  |  |  | aLBM (kg; DXA) | 17/19  (mITT) | Mean change (95%-CI):  IG: +0.1 (-0.1 to +0.4)  CG: +0.2 (0.0 to +0.4)  P=0.54 | |
| Park et al. 2018 (7), Korea | Community-dwelling (pre-)frail older adults aged 70-85 y at risk of malnutrition (MNA ≤23.5); no kidney or liver failure; able to walk; 65% female; mean age: 77 ± 4 y; mean BMI: 24 ± 3 kg/m^2^; Asian | Whey protein. Multiple 10-g packs of protein powder (9.3 g whey protein/pack), dissolved in 340 ml tea, were provided in addition to habitual protein intake up to 1.2 g/kg BW/d (IG1) and 1.5 g/kg BW/d (IG2), [A] | CHO placebo. Multiple 10-g packs of CHO powder (9.3 g maltodextrin per/pack), dissolved in 340 ml tea, were provided in addition to habitual protein intake up to 0.8 g/kg (CG) or 1.2 g/kg (IG1)  (CG were given only CHO powder, IGs were given a combination of protein and CHO powder.) | IG1: 98%, IG2: 96%, CG: 97% | Baseline:  IG1: 0.77 ± 0.24, IG2: 0.80 ± 0.21, CG: 0.84 ± 0.28  Follow-up:  IG1: 1.18 ± 0.23, IG2: 1.37 ± 0.26, CG: 0.90 ± 0.38 | 12 wk | Some concerns | aSMM^§^ (kg; DXA) | 40/40/40 (ITT) | **P for time*group interaction<0.05**  (aSMM increased more in IG2, but not IG1, than in CG)  (Result was similar for relative aSMM (% of BW): P for time*group interaction<0.05; % aSMM increased more in IG2, but not IG1, than in CG) | |
|  |  |  |  |  |  |  |  | aSMM relative to BW^§^ (%) | 40/40/40 (ITT) | **P for time*group interaction<0.05**  (aSMM/BW increased more in IG2, but not IG1, than in CG) | |
|  |  |  |  |  |  |  |  | aSMM relative to squared height^§^ (kg/m^2^) | 40/40/40 (ITT) | **P for time*group interaction<0.05**  (aSMM/height^2^ increased more in IG2, but not IG1, than in CG) | |
|  |  |  |  |  |  |  |  | aSMM relative to BMI^§^ | 40/40/40 (ITT) | **P for time*group interaction<0.05**  (aSMM/BMI increased more in IG2, but not IG1, than in CG) | |
| Wright et al. 2018 (8), USA | Older adults aged 50-80 y with overweight or obesity (BMI of 25-38 kg/m^2^); without diabetes; 45% female; mean age: 70 ± 5 y; mean BMI: 31 ± 3 kg/m^2^; race NR | High-protein diet: 1.4 g/kg BW/d (~27 E% protein, ~43 E% CHO, ~30 E% fat). Majority of additional protein (59% = ~30 g = ~0.35 g/kg BW/d) came from eggs, [C] | Normal-protein diet: 0.8 g/kg BW/d (~15 E% protein, ~55 E% CHO, ~30 E% fat)    (Normal-protein diet provided on average ~50 g/d less protein than high-protein diet.) | 91% (overall) | Baseline:  IG: 84 ± 15 g/d, CG: 79 ± 15 g/d (calculated by using mean BW: IG: 0.93 g/kg BW/d, CG: 0.88 g/kg BW/d  Follow-up:  NR | 12 wk | High | Total LBM (kg; DXA) | 12/10  (PP) | Mean change ± SD:  IG: -0.28 ± 0.97 CG: -1.29 ± 0.97  **P for time*group interaction=0.05**  (No significant change in total fat mass (P>0.05)) | |
|  |  |  |  |  |  |  |  | Trunk LBM (kg; DXA) | 12/10  (PP) | Mean change ± SD:  IG: +0.12 ± 0.63  CG: -0.68 ± 0.72  **P for time*group interaction=0.015** | |
|  |  |  |  |  |  |  |  | aLBM (kg; DXA) | 12/10  (PP) | Mean change ± SD:  IG: -0.39 ± 0.71 CG: -0.58 ± 0.66  Time*group interaction NS | |
|  |  |  |  |  |  |  |  | Muscle CSA, thigh (mm^2^; MRI) | 18 (total; PP) | Mean change ± SD (x 10^4^):  IG: +9.3 ± 114.0  CG: -29.2 ± 65.1 Time*group interaction NS | |
|  |  |  |  |  |  |  |  | Muscle volume, thigh (mm^3^; MRI) | 18 (total; PP) | Mean change ± SD (x 10^4^):  IG: -16.2 ± 60.5 CG: -7.4 ± 28.3  Time*group interaction NS | |
|  |  |  |  |  |  |  |  | Muscle CSA, calf (mm^2^; MRI) | 15 (total; PP) | Mean change ± SD (x 10^4^):  IG: -1.9 ± 4.8  CG: +2.0 ± 15.5  Time*group interaction NS | |
|  |  |  |  |  |  |  |  | Muscle volume, calf (mm^3^; MRI) | 15 (total; PP) | Mean change ± SD (x 10^4^):  IG: -0.5 ± 3.0 CG: +1.2 ± 6.9  Time*group interaction NS | |
| Zhu et al. 2015 (9), Australia  Same RCT as Hodgson et al. 2012 (10) and Zhu et al. 2011 (11) | Community-dwelling older women aged 70-80 with habitual protein intake <1.5 g/kg BW/d; without metabolic bone disease, osteoporotic fracture, diabetes, hepatic or renal insufficiency; mean age: 74 ± 3 y; mean BMI: 27 ± 4 kg/m^2^; Caucasian | Whey protein isolate. 250 ml skim milk-based high-protein supplement drink containing 30 g of whey protein + calcium, [A] | CHO placebo. 250 ml skim milk-based supplement drink containing 2.1 g of protein and isocaloric amount of maltodextrin + calcium | 87.1% in IG and 80.8% in CG (P=0.03) | Baseline:  IG: 1.2 ± 0.3, CG: 1.1 ± 0.3 Follow-up (1 y):  IG: 1.4 ± 0.4, CG: 1.1 ± 0.3  Follow-up (2 y): IG: 1.4 ± 0.4, CG: 1.1 ± 0.4 | 1 y and 2 y | Some concerns | Arm LBM^§^ (kg; DXA) | 1 y: 101/95  (mITT) | Mean change ± SEM:  IG: +0.02 ± 0.02 CG: +0.09 ± 0.02  Time*group interaction NS | |
|  |  |  |  |  |  |  |  |  | 2 y:  93/88  (mITT) | Mean change ± SEM:  IG: 0 ± 0.02 CG: +0.03 ± 0.02  Time*group interaction NS | |
|  |  |  |  |  |  |  |  | Leg LBM^§^ (kg; DXA) | 1 y: 101/95  (mITT) | Mean change ± SEM:  IG: +0.18 ± 0.05 CG: +0.18 ± 0.06  Time*group interaction NS | |
|  |  |  |  |  |  |  |  |  | 2 y:  93/88  (mITT) | Mean change ± SEM:  IG: 0 ± 0.06 CG: +0.03 ± 0.02  Time*group interaction NS | |
|  |  |  |  |  |  |  |  | aLBM^§^ (kg; DXA) | 1 y: 101/95  (mITT) | Mean change ± SEM:  IG: +0.20 ± 0.06 CG: +0.27 ± 0.07  Time*group interaction NS | |
|  |  |  |  |  |  |  |  |  | 2 y:  93/88  (mITT) | Mean change ± SEM:  IG: -0.03 ± 0.07 CG: +0.03 ± 0.08  Time*group interaction NS | |
|  |  |  |  |  |  |  |  | aLBM relative to squared height^§^ (kg/m^2^) | 1 y: 101/95  (mITT) | Mean change ± SEM:  IG: +0.09 ± 0.02 CG: +0.11 ± 0.02  Time*group interaction NS | |
|  |  |  |  |  |  |  |  |  | 2 y:  93/88  (mITT) | Mean change ± SEM:  IG: +0.02 ± 0.03 CG: +0.05 ± 0.03  Time*group interaction NS | |
|  |  |  |  |  |  |  |  | Muscle CSA, calf^§^ (cm^2^; QCT) | 1 y: 101/95  (mITT) | Mean change ± SEM:  IG: -0.01 ± 0.13 CG: +0.13 ± 0.15  Time*group interaction NS | |
|  |  |  |  |  |  |  |  |  | 2 y:  93/88  (mITT) | Mean change ± SEM:  IG: -0.71 ± 0.15 CG: -0.83 ± 0.17  Time*group interaction NS | |
| In the context of (concomitant) physical exercise | | | | | | | | | | |  |
| Arnarson et al. 2013 (12), Iceland  Same RCT as Ramel et al. 2013 (13) | Community-dwelling older men and women aged ≥65 y;  without major orthopaedic disease or musculoskeletal disorders; 58% female; mean age: 74 ± 6 y (range: 65-91 y); mean BMI: 29 ± 5 kg/m^2^ (82% ≥25 kg/m^2^); race NR | Whey protein. Drink containing 20 g of whey protein isolate (+ 20 g of CHO), consumed after WBR (so only on training days), [A]  WBR, 3 times/wk, 3 sets of 6-8 reps, 75-80% 1RM | CHO placebo. Drink containing 40 g of CHO, consumed after WBR (so only on training days)  WBR, 3 times/wk, 3 sets of 6-8 reps, 75-80% 1RM | NR | Baseline:  IG: 1.00 ± 0.26, CG: 0.92 ± 0.30  Follow-up:  IG: 1.06 ± 0.23, CG 0.89 ± 0.23 | 12 wk | High | Total LBM^§^ (kg; DXA) | 75/66 | Mean change ± SD:  IG: +0.7 ± 1.1 CG: +0.9 ± 1.5  P=0.365 | |
|  |  |  |  |  |  |  |  | aSMM^§^ (kg; DXA) | 75/66 | Mean change ± SD:  IG: +0.6 ± 1.2 CG: +0.5 ± 0.8  P=0.938 | |
| Campbell et al. 1995 (14), USA | Generally healthy older men and women aged 56-80 y; 33% female; mean age: 65 ± 3 y; (estimated) mean BMI: 26 kg/m^2^; race NR | High-protein diet (2*RDA = 1.6 g/kg BW/d). Lacto-ovo-vegetarian diet providing 0.6 g protein/kg BW + milk-based beverages providing 1.0 g protein/kg BW + multivitamin-multimineral supplement, [B]  WBR, 3 times/d, 2-3 sets of 8-12 reps, 80% 1RM | Low-protein diet (1*RDA = 0.8 g/kg BW/d). Lacto-ovo-vegetarian diet providing 0.6 g protein/kg BW + milk-based beverages providing 0.2 g protein/kg BW (non-protein intake was 55% CHO and 45% fat) + multivitamin-multimineral supplement  WBR, 3 times/d, 2-3 sets of 8-12 reps, 80% 1RM | NR | Baseline (after a 2-wk run-in period):  IG: 1.62 ± 0.02, CG: 0.80 ± 0.02  Follow-up:  Similar to baseline values | 12 wk | Some concerns | Fat-free mass (kg, hydrostatic weighing)^d^ | 6/6 (ITT) | Time*group interaction NS | |
|  |  |  |  |  |  |  |  | Muscle CSA, thigh (cm^2^; CT) | 6/6 (ITT) | Authors reported no results for time*group interaction (repeated measures ANOVA), which suggests that protein has no effect | |
| Chalé et al. 2013 (15), USA | Community-dwelling, mobility-limited, sedentary older adults aged 70-85 y with habitual protein intake ≥0.8 g/kg BW/d; 59% female; mean age: 78 ± 4 y; mean BMI: 27 ± 3 kg/m^2^; race: White (70%), Black (16%), Asian (4%), other or unknown (10%) | Whey protein concentrate: 20 g consumed after breakfast and 20 g consumed after evening meal each day (total: 40 g whey protein/d). On training days, one serving was consumed immediately after WBR, [A]  WBR, 3 times/wk, 10 reps, 80% 1RM | CHO placebo. Isocaloric amount consumed after breakfast and after evening meal each day. On training days, one serving was consumed immediately after WBR.  WBR, 3 times/wk, 10 reps, 80% 1RM | IG: 72.1 ± 29.3%, CG: 82.3 ± 21.9% | Baseline: IG: 0.97, CG: 0.98  Follow-up:  NR | 6 mo | Some concerns | Total LBM^§^ (kg; DXA) | 42/38  (ITT) | MD (95%-CI):  +0.26 (-0.43 to +0.95)  NS | |
|  |  |  |  |  |  |  |  | Muscle CSA, thigh^§^ (cm^2^; CT) | 42/38  (ITT) | MD (95%-CI):  +1 (-1 to +4)  NS | |
| Fernandes et al. 2018 (16), Brazil  Same RCT as Sugihara Junior et al. 2018 (17) | Older women aged ≥60 y; physically independent; free from cardiac or orthopaedic dysfunction; protein intake <1.2 g/kg BW; mean age: 68 ± 4 y; mean BMI: 26 ± 3 kg/m^2^; race NR | Whey protein. 35 g of hydrolysed whey protein containing 27.1 g of protein, dissolved in 200 ml sugar-free soft drink, ingested after WBR (so only on training days), [A]  WBR, 3 times/wk, 3 sets of 8-12RM | CHO placebo. 35 g of maltodextrin, dissolved in 200 ml sugar-free soft drink, ingested after WBR (so only on training days)  WBR, 3 times/wk, 3 sets of 8-12RM | NR | Baseline:  IG: 0.85 ± 0.1, CG: 0.81 ± 0.1 Follow-up:  IG: 1.4 ± 0.1, CG: 0.87 ± 0.1 | 12 wk | High | Total LST (kg; DXA) | 16/16 | Mean % change:  IG: +3.8  CG: +2.0  **P for time*group interaction<0.05** | |
| Mitchell et al. 2015 (18), Canada | Generally healthy older men; free of musculoskeletal or metabolic disorders; recreationally active; mean age: 74 ± 5 y; mean BMI: 27 ± 3 kg/m^2^; race NR | 500-ml of chocolate milk containing 14 g of protein, consumed <15 min after WBR on training days and with breakfast on non-training days, [B]  WBR, 3 times/wk, 3-4 sets, 75-85% 1RM | CHO placebo. 500-ml drink with isocaloric amount of CHO, consumed <15 min after WBR on training days and with breakfast on non-training days  WBR, 3 times/wk, 3-4 sets, 75-85% 1RM | NR | Baseline:  NR  Follow-up:  NR | 12 wk | High | Muscle fibre area (biopsy) | 16 (total) | Time*group interaction NS | |
| Nabuco et al. 2018 (19), Brazil  Same RCT as Nabuco et al. 2019a (20) and Nabuco et al. 2019b (21) | Older women aged ≥60, physically independent, free from cardiac or orthopaedic dysfunction; mean age: 67 ± 7 y; mean BMI: 25 ± 5 kg/m^2^; race NR | Whey protein. 35 g of hydrolysed whey protein supplement containing 27.1 g protein (+ 5.2 g CHO), mixed with non-caloric drink. IG1: protein before and placebo after WBR; IG2: placebo before and protein after WBR (so only on training days), [A]  WBR, 3 times/wk, 3 sets of 8-12 reps | CHO placebo containing 0.3 g protein and 33.3 g CHO, mixed with non-caloric drink; one before and one after WBR (so only on training days)  WBR, 3 times/wk, 3 sets of 8-12 reps | NR | Baseline:  IG1: 0.92 ± 0.20, IG2: 0.94 ± 0.36, CG: 0.95 ± 0.27  Follow-up:  IG1: 1.38 ± 0.26, IG2: 1.49 ± 0.46, CG: 1.0 ± 0.25 | 12 wk | Some concerns | Upper limb LST (kg; DXA) | 22/21/23  (mITT) | Mean % change ± SD:  IG1: +3.4 ± 3.0 IG2: +5.9 ± 4.3  CG: +4.1 ± 3.5  P for time*group interaction=0.156 | |
|  |  |  |  |  |  |  |  | Lower limb LST (kg; DXA) | 22/21/23  (mITT) | Mean % change ± SD:  IG1: +3.2 ± 2.9* IG2: +3.7 ± 2.2*  CG: +1.1 ± 2.2  *** P<0.05 compared to CG** | |
|  |  |  |  |  |  |  |  | SMM (kg; DXA^f^) | 22/21/23  (mITT) | Mean % change ± SD:  IG1: +3.4 ± 2.9* IG2: +4.2 ± 2.3*  CG: +2.0 ± 2.1  *** P<0.05 compared to CG** | |
| Nabuco et al. 2019a (20), Brazil  Same RCT as Nabuco et al. 2018 (19) and Nabuco et al. 2019b (21) | Older women aged ≥60 y, physically independent, free from cardiac or orthopaedic dysfunction; mean age: 67 ± 7 y; mean BMI: 25 ± 5 kg/m^2^; race NR | Whey protein. 35 g of hydrolysed whey protein supplement containing 27.1 g protein (+ 5.2 g CHO), mixed with non-caloric drink. IG1: protein before and placebo after WBR; IG2: placebo before and protein after WBR (so only on training days), [A]  WBR, 3 times/wk, 3 sets of 8-12 reps | CHO placebo containing 0.3 g protein and 33.3 g CHO, mixed with non-caloric drink; one before and one after WBR (so only on training days)  WBR, 3 times/wk, 3 sets of 8-12 reps | NR | Baseline:  IG1: 0.92 ± 0.20, IG2: 0.94 ± 0.36, CG: 0.95 ± 0.27  Follow-up:  IG1: 1.38 ± 0.26, IG2: 1.49 ± 0.46, CG: 1.0 ± 0.25 | 12 wk | Some concerns | aLST (kg; DXA) | 22/21/23  (mITT) | Mean % change:  IG1: +3.1  IG2: +2.6  CG: +2.7  P for time*group interaction=0.600 | |
| Nabuco et al. 2019b (21), Brazil  Same RCT as Nabuco et al. 2018 (19) and Nabuco et al. 2019a (20) | Older women aged ≥60 y, physically independent, free from cardiac or orthopaedic dysfunction; mean age: 67 ± 7 y; mean BMI: 25 ± 5 kg/m^2^; race NR | Whey protein. 35 g of hydrolysed whey protein supplement containing 27.1 g protein (+ 5.2 g CHO), mixed with non-caloric drink. IG1: protein before and placebo after WBR; IG2: placebo before and protein after WBR (so only on training days), [A]  WBR, 3 times/wk, 3 sets of 8-12 reps | CHO placebo containing 0.3 g protein and 33.3 g CHO, mixed with non-caloric drink; one before and one after WBR (so only on training days)  WBR, 3 times/wk, 3 sets of 8-12 reps | NR | Baseline:  IG1: 0.92 ± 0.20, IG2: 0.94 ± 0.36, CG: 0.95 ± 0.27  Follow-up:  IG1: 1.38 ± 0.26, IG2: 1.49 ± 0.46, CG: 1.0 ± 0.25 | 12 wk | Some concerns | Total LST (kg; DXA) | 22/21/23  (mITT) | Mean % change:  IG1: +2.7*  IG2: +3.7*  CG: +1.5  *** P<0.05 compared to CG** | |
| Nabuco et al. 2019c (22), Brazil | Older women aged ≥60 y with sarcopenic obesity (body fat mass ≥35% and ALST <15.02 kg); physically independent; free from cardiac or orthopaedic dysfunction; mean age: 69 ± 4 y; mean BMI: 27 ± 3 kg/m^2^; race NR | Whey protein. 35 g of hydrolysed whey protein supplement, mixed with non-caloric drink, ingested after WBR (so only on training days), [A]  WBR, 3 times/wk, 3 sets of 8-12 reps | CHO placebo. Isocaloric amount of maltodextrin mixed with non-caloric drink, ingested after WBR (so only on training days)  WBR, 3 times/wk, 3 sets of 8-12 reps | NR | Baseline  IG: 0.93 ± 0.36, CG: 0.97 ± 0.28  Follow-up:  IG: 1.0 ± 0.23, CG: 1.0 ± 0.19 (without supplementation) | 12 wk | Some concerns | Total LST (kg; DXA) | 13/13  (ITT) | Mean % change:  IG: +3.8  CG: +1.0  **P for time*group interaction<0.001** | |
|  |  |  |  |  |  |  |  | Lower LST (kg, DXA) | 13/13  (ITT) | Mean % change:  IG: +4.8  CG: +1.3  **P for time*group interaction<0.001** | |
|  |  |  |  |  |  |  |  | aLST (kg, DXA) | 13/13  (ITT) | Mean % change:  IG: +6.1  CG: +2.4  **P for time*group interaction<0.001** | |
| Sugihara Junior et al. 2018 (17), Brazil  Same RCT as Fernandes et al. 2018 (16) | Older women aged ≥60 y; physically independent; free from cardiac or orthopaedic dysfunction; protein intake <1.2 g/kg BW; mean age: 68 ± 4 y; mean BMI: 26 ± 3 kg/m^2^; race NR | Whey protein. 35 g of hydrolysed whey protein containing 27.1 g of protein, dissolved in 200 ml sugar-free soft drink, ingested after WBR (so only on training days), [A]  WBR, 3 times/wk, 3 sets of 8-12RM | CHO placebo. 35 g of maltodextrin, dissolved in 200 ml sugar-free soft drink, ingested after WBR (so only on training days)  WBR, 3 times/wk, 3 sets of 8-12RM | NR | Baseline:  IG: 0.85 ± 0.1, CG: 0.81 ± 0.1 Follow-up:  IG: 1.4 ± 0.1, CG: 0.87 ± 0.1 | 12 wk | High | Upper limb LST (kg; DXA) | 15/16 | Mean % change:  IG: +5.0  CG: +2.5  P for time*group interaction=0.68 | |
|  |  |  |  |  |  |  |  | Lower limb LST (kg; DXA) | 15/16 | Mean % change:  IG: +3.4  CG: +0.8  P for time*group interaction=0.11 | |
|  |  |  |  |  |  |  |  | SMM (kg; DXA^g^) | 15/16 | Mean % change:  IG: +4.8  CG: +2.3  **P for time*group interaction=0.02** | |
| Ten Haaf et al. 2019 (23), The Netherlands | Physically active older adults aged ≥65 y with habitual protein intake <1.0 g/kg BW; without type 2 diabetes, cancer, renal insufficiency (eGFR <30) or COPD; median age: 69 (range 67-73) y; mean BMI: 27 ± 3 kg/m^2^; Caucasian (except for 1 Asian participant) | Milk-protein concentrate. 250-ml protein drink containing 15.5 g protein (+ 1.1 g fat + 14.5 g lactose) consumed twice a day (total: 31 g protein/d), [A]  Training (walking) for the Nijmegen Four Days Marches | CHO placebo. 250-ml isocaloric drink containing 1.1 g protein (+ 5.2 g fat + 36 g CHO) consumed twice daily  Training (walking) for the Nijmegen Four Days Marches | IG: 96 ± 3%, CG: 95 ± 3% | Baseline:  IG: 0.86 ± 0.23, CG: 0.92 ± 0.24  Follow-up:  IG: 0.92 ± 0.27, CG: 0.97 ± 0.23 (without supplementation) | 12 wk | Some concerns | Total LBM^§^ (kg; DXA) | 58/56  (PP) | Mean change ± SD:  IG: +0.54 ± 1.13  CG: +0.31 ± 1.03  P for time*group interaction=0.27  For relative total LBM (% of BW), mean change ± SD:  IG: +0.93 ± 1.22, CG: +0.44 ± 1.4, P for time*group interaction=0.046. Mainly a result of the significantly greater decrease in total fat mass (kg) in IG than in CG (P=0.013). | |
| Thomson et al. 2016 (24), Australia | Older adults aged 50-79 y and BMI of 20-35 kg/m^2^ who are physically active (but not engaged in formal exercise); without major chronic diseases (e.g. diabetes, cancer, metabolic disease, cardiac abnormalities, musculoskeletal injury); 55% female; mean age: 62 ± 7 y; mean BMI: 28 ± 4 kg/m^2^; race NR | High-protein diet. Individualised diet providing 1.0 g/kg BW/d + ~27 g dairy-based protein drink each day (IG1); Individualised diet providing 1.0 g/kg BW/d + ~27 g soy protein drink each day (IG2), [B]  WBR, 3 times/wk, 1-3 sets of 8-20 reps, 8RM | CHO placebo. Individualised diet providing 1.0 g/kg BW/d + ~27 g CHO drink each day  WBR, 3 times/wk, 1-3 sets of 8-20 reps, 8RM | IG1: 97%, IG2: 98%, CG: 98% | Baseline:  IG1: 1.06 ± 0.10, IG2: 1.08 ± 0.09, CG: 1.02 ± 0.05  Follow-up:  IG1: 1.42 ± 0.14, IG2: 1.45 ± 0.14, CG: 1.08 ± 0.05 | 12 wk | High | Total LBM (kg) | 34/26/23  (PP^h^) | Mean change ± SD:  IG1: +1.0 ± 1.0  IG2: +1.4 ± 1.2  CG: +0.8 ± 1.1 Between-group difference NS (P≥0.1) | |

Abbreviations: ADP, air-displacement plethysmography; aLBM; appendicular lean body mass; ALST, appendicular lean soft tissue; aSMM, appendicular skeletal muscle mass; BMI, body mass index; BW, body weight; CSA, cross-sectional area; CI, confidence interval; CG, control group; CHO, carbohydrates; CKD, chronic kidney disease; COPD, chronic obstructive pulmonary disease; CVD, cardiovascular disease; DXA, dual-energy X-ray absorptiometry; E%, percentage of energy intake; EAA, essential amino acids; IG, intervention group; ITT, intention-to-treat analysis or modified intention-to-treat analysis (mITT; only those with missing outcome data were excluded from the analytic sample); IU, international units; LBM, lean body mass; LSMD, least square mean difference; LST, Lean soft tissue; LTM, lean tissue mass; MD, mean difference (i.e. difference in within-group change); mg, milligram; MNA, Mini Nutritional Assessment; mo, months; MRI, magnetic resonance imaging; *n*, number; NR, not reported; NS, not significant; PP, per-protocol analysis; QCT; quantitative computed tomography; RDA, recommended dietary allowance; reps, repetitions; RM, repetition maximum; RoB, risk of bias; SD, standard deviation; SEM, standard error of the mean; SMM, skeletal muscle mass; US, B-mode ultrasound; WBR, whole-body resistance training; wk, weeks; y, years.

Statistically significant effects are shown in bold.

^§^ Sufficient statistical power to detect an effect is to be expected, based on the sample size calculation.

^a^ ‘Protein type’ indicates the way in which a higher protein intake was achieved and is categorized into ‘pure’ protein or amino acids (or essential amino acids) (A), specific product with a high protein content (B) or high-protein diets (C).

^b^ Risk of bias was assessed using the RoB 2 Cochrane collaboration tool and scored as ‘low’ (L), ‘some concerns’ (SC) or ‘high’ (H).

^c^ Two subjects completed both diet protocols, with an interval of ≥4 months between each study. Data analyses were performed with sample sizes of 12 and 10, with and without the two subjects who completed both diet protocols, respectively. The results were the same for both; thus, all 12 subjects were considered individually for this report.

^d^ Estimated from body density and total body water using the three-compartment model of Siri (25).

^e^ Air-displacement plethysmography (ADP) is conducted using a Bod Pod.

^f^ (Intermuscular adipose tissue-free) skeletal muscle mass (SMM) was calculated with a predictive equation obtained from Kim et al. (26)

^g^ Skeletal muscle mass (SMM) was calculated with a predictive equation obtained from Kim et al. (27)

^h^ Intention-to-treat analysis demonstrated a similar pattern to the per-protocol analysis (data not shown).

Supplemental Table 2

Results from randomized controlled trials on the effect of increased protein intake on muscle strength in older adults, grouped according to whether or not the protein intervention was carried out in the context of (concomitant) physical exercise

| Study, country | Study population | Exposure (IG), [protein type^a^] | Comparison (CG) | Compliance | Total protein intake (g/kg BW/d) | Study duration | Risk of bias^b^ | Outcome | Analytic *n* IG/CG | Results | |
| --- | --- | --- | --- | --- | --- | --- | --- | --- | --- | --- | --- |
| Not in the context of (concomitant) physical exercise | | | | | | | | | | |  |
| Bhasin et al. 2018 (1), USA | Community-dwelling older men aged ≥65 y with moderate physical function limitations and with habitual protein intake ≤0.83 g/kg BW/d; mean age: 73 ± 6 y; mean BMI: 30.3 ± 4.9 kg/m^2^; race NR | Individualised diets providing 0.7 g protein/kg BW/d with additional discretionary foods (0.1 g protein/kg BW) and protein supplements (0.5 g/kg BW; 50:50 casein:whey mix) to achieve a total of 1.3 g/kg BW/d, [A,B] | Individualised diets providing 0.7 g protein/kg BW/d with additional discretionary foods (0.1 g protein/kg BW) and placebo supplements (0.5 g CHO/kg BW) to achieve a total of 0.8 g/kg BW/d | Foods (4-6 mo):  IG: 77.1 ± 13%, CG: 74.5 ± 23.2%  Supplements (4-6 mo):  IG: 91.2 ± 12.4%, CG: 92.6 ± 11.0% | Baseline:  IG: 0.72 ± 0.11, CG: 0.69 ± 0.15  Follow-up (1-3 mo):  IG: 1.18 ± 0.15, CG: 0.84 ± 0.07  Follow-up (4-6 mo): IG: 1.17 ± 0.13, CG: 0.81 ± 0.10 | 6 mo | Some concerns | Leg press strength, 1RM (N) | 31/32  (mITT) | MD (95%-CI):  +0.89 (-86.9 to +88.7)  P=0.98 | |
|  |  |  |  |  |  |  |  | Chest press strength, 1RM (N) | 31/34  (mITT) | MD (95%-CI):  -11.8 (-31.6 to +7.9)  P=0.24 | |
|  |  |  |  |  |  |  |  | Leg press peak power, 60% 1RM (W) | 29/32  (mITT) | MD (95%-CI):  +26.4 (-10.5 to +63.4)  P=0.16 | |
| Dillon et al. 2009 (2), USA | Community-dwelling older women; without vascular disease, hypertension or cardiac abnormality; mean age: 68 ± 2 y; mean BMI NR; race NR | EAA. 7.5 g EAA, ingested twice a day in between meals (total: 15 g EAA/d), [A] | CHO placebo. Isocaloric amount of lactose, ingested twice a day in between meals | NR | Baseline:  NR  Follow-up: NR | 3 mo | High | Bicep curl, 1RM | 7/7 | Authors reported no results for time*group interaction (ANOVA), which suggests that protein has no effect | |
|  |  |  |  |  |  |  |  | Triceps extension, 1RM | 7/7 | Authors reported no results for time*group interaction (ANOVA), which suggests that protein has no effect | |
|  |  |  |  |  |  |  |  | Leg extension, 1RM | 7/7 | Authors reported no results for time*group interaction (ANOVA), which suggests that protein has no effect | |
|  |  |  |  |  |  |  |  | Leg curl, 1RM | 7/7 | Authors reported no results for time*group interaction (ANOVA), which suggests that protein has no effect | |
| Ispoglou et al. 2016 (3), UK | Community-dwelling older men and women aged 65-75 y; good health, without major chronic diseases (e.g. diabetes, vascular disease, hypertension); 44% female; mean age: 72 ± 3 y; mean BMI: 27 ± 4 y; race: NR | EAA. Standard EAA mixture with 20% leucine (IG1) and with 40% leucine (IG2), ingested at breakfast and dinner (total: 0.21 g/kg BW/d = ~11-21 g/d), [A] | CHO placebo. Isocaloric amount of lactose, ingested at breakfast and dinner | 74-83% | Baseline: 0.95-1.10 g/kg BW/d  Follow-up: 1.02-1.08 g/kg BW/d (without supplementation) | 12 wk | High | Relative handgrip strength (N/kg BW) | 8/8/9  (PP) | Mean % change ± SD:  IG1: +11.5 ± 23.9  IG2: +4.8 ± 7.5  CG: -0.2 ± 8.9  P=0.300 | |
|  |  |  |  |  |  |  |  | 30-s arm-curl test (n) | 8/8/9  (PP) | Mean % change ± SD:  IG1: +15.0 ± 20.0  IG2: +8.3 ± 13.7  CG: +0.9 ± 11.2  P=0.193 | |
| Mitchell et al. 2017 (5), New Zealand | Community-dwelling older men aged >70 y with BMI of 18-35 kg/m^2^; able to perform ADLs without mobility aids; without major chronic diseases (e.g. cancer, diabetes, thyroid diseases); mean age: 74 y (range: 70-81 y); mean BMI: 28 ± 4 kg/m^2^; race NR | High-protein diet (2*RDA = 1.6 g/kg BW/d), omnivorous, 28-31 E% fat, [C] | Low-protein diet (1*RDA = 0.8 g/kg BW/d), omnivorous, 28-31 E% fat; difference made up of CHO | IG: 97.5%, CG: 98.9% | Baseline:  IG: 1.1 ± 0.3, CG: 1.2 ± 0.4  Follow-up:  IG: 1.7 ± 0.1, CG: 0.9 ± 0.1 | 10 wk | High | Handgrip strength (kg) | 14/15  (PP) | P for time*group interaction=0.167 | |
|  |  |  |  |  |  |  |  | Knee extension MVC (Nm) | 14/15  (PP) | Mean change ± SD:  IG: +7.5 ± 22.9  CG: -8.6 ± 24.2  P for time*group interaction=0.120 | |
|  |  |  |  |  |  |  |  | Knee extension peak power (W) | 14/15  (PP) | Mean change ± SD:  IG: +26.6 ± 47.7  CG: -11.7 ± 31.0  **P for time*group interaction=0.012** | |
| Ottestad et al. 2017 (6), Norway | Community-dwelling older adults aged ≥70 y; relatively healthy (no diabetes, CVD, cancer, COPD, CKD); not malnourished; with reduced muscle strength or performance; 67% female; mean age: 77 ± 5 y; mean BMI: 27 ± 5 kg/m^2^; race NR | Protein-enriched milk. 400 ml drink containing 20 g protein (80:20 casein:whey mix, consumed twice a day (total: 40 g protein/d), [B] | CHO placebo. 400 ml drink containing an isocaloric amount of CHO, consumed twice a day | IG: 97.8 ± 3.8%, CG: 96.8 ± 5.7% | Baseline:  IG: 1.0 ± 0.3, CG: 1.0 ± 0.3  Follow-up:  IG: 1.4 ± 0.5, CG: 0.9 ± 0.4 | 12 wk | High | Leg press strength, 1RM (kg) | 16/17  (mITT) | Mean change (95%-CI):  IG: +5.7 (-1.7 to +13.1)  CG: +6.2 (-2.2 to +14.6)  P=0.93 | |
|  |  |  |  |  |  |  |  | Chest press strength, 1RM (kg) | 17/18  (mITT) | Mean change (95%-CI):  IG: +1.3 (+0.1 to +2.5)  CG: +1.5 (0.0 to +3.0)  P=0.85 | |
|  |  |  |  |  |  |  |  | Handgrip strength, dominant side (kg) | 17/18  (mITT) | Mean change (95%-CI):  IG: +0.1 (-0.7 to +1.0)  CG: -0.5 (-1.5 to +0.5)  P=0.27 | |
|  |  |  |  |  |  |  |  | Handgrip strength, non-dominant side (kg) | 17/19  (mITT) | Mean change (95%-CI):  IG: +0.5 (-0.5 to +1.5)  CG: +0.5 (-0.4 to +1.4)  P=0.99 | |
| Park et al. 2018 (7), Korea | Community-dwelling (pre-)frail older adults aged 70-85 y at risk of malnutrition (MNA ≤23.5); no kidney or liver failure; able to walk; 65% female; mean age: 77 ± 4 y; mean BMI: 24 ± 3 kg/m^2^; Asian | Whey protein. Multiple 10-g packs of protein powder (9.3 g whey protein/pack), dissolved in 340 ml tea, were provided in addition to habitual protein intake up to 1.2 g/kg BW/d (IG1) and 1.5 g/kg BW/d (IG2), [A] | CHO placebo. Multiple 10-g packs of CHO powder (9.3 g maltodextrin per/pack), dissolved in 340 ml tea, were provided in addition to habitual protein intake up to 0.8 g/kg (CG) or 1.2 g/kg (IG1)  (CG were given only CHO powder, IGs were given a combination of protein and CHO powder.) | IG1: 98%, IG2: 96%, CG: 97% | Baseline:  IG1: 0.77 ± 0.24, IG2: 0.80 ± 0.21, CG: 0.84 ± 0.28  Follow-up:  IG1: 1.18 ± 0.23, IG2: 1.37 ± 0.26, CG: 0.90 ± 0.38 | 12 wk | Some concerns | Handgrip strength (kg) | 40/40/40 (ITT) | P for time*group interaction=0.553 | |
| Zhu et al. 2015 (9), Australia  Same RCT as Hodgson et al. 2012 (10) and Zhu et al. 2011 (11) | Community-dwelling older women aged 70-80 y with habitual protein intake <1.5 g/kg BW/d; without metabolic bone disease, osteoporotic fracture, diabetes, hepatic or renal insufficiency; mean age: 74 ± 3 y; mean BMI: 27 ± 4 kg/m^2^; Caucasian | Whey protein isolate. 250 ml skim milk-based high-protein supplement drink containing 30 g of whey protein + calcium, [A] | CHO placebo. 250 ml skim milk-based supplement drink containing 2.1 g of protein and isocaloric amount of maltodextrin + calcium | 87.1% in IG and 80.8% in CG (P=0.03) | Baseline:  IG: 1.2 ± 0.3, CG: 1.1 ± 0.3 Follow-up (1 y):  IG: 1.4 ± 0.4, CG: 1.1 ± 0.3  Follow-up (2 y): IG: 1.4 ± 0.4, CG: 1.1 ± 0.4 | 1 y and 2 y | Some concerns | Handgrip strength (kg) | 1 y: 99/94 (mITT) | Mean change ± SEM:  IG: -0.87 ± 0.40 CG: -1.11 ± 0.40  Time*group interaction NS | |
|  |  |  |  |  |  |  |  |  | 2 y:  93/88 (mITT) | Mean change ± SEM:  IG: -1.09 ± 0.41 CG: -1.53 ± 0.42  Time*group interaction NS | |
|  |  |  |  |  |  |  |  | Ankle dorsiflexion strength (kg) | 1 y: 99/94 (mITT) | Mean change ± SEM:  IG: +1.10 ± 0.38 CG: +1.44 ± 0.40  Time*group interaction NS | |
|  |  |  |  |  |  |  |  |  | 2 y:  93/88  (mITT) | Mean change ± SEM:  IG: +2.15 ± 0.48  CG: +2.71 ± 0.41  Time*group interaction NS | |
|  |  |  |  |  |  |  |  | Knee flexor strength (kg) | 1 y: 99/94  (mITT) | Mean change ± SEM:  IG: +1.81 ± 0.38  CG: +1.65 ± 0.41  Time*group interaction NS | |
|  |  |  |  |  |  |  |  |  | 2 y:  93/88  (mITT) | Mean change ± SEM:  IG: +3.18 ± 0.38  CG: +2.36 ± 0.49  Time*group interaction NS | |
|  |  |  |  |  |  |  |  | Knee extensor strength (kg) | 1 y: 99/94  (mITT) | Mean change ± SEM:  IG: +2.08 ± 0.54  CG: +2.83 ± 0.67  Time*group interaction NS | |
|  |  |  |  |  |  |  |  |  | 2 y:  93/88  (mITT) | Mean change ± SEM:  IG: +3.36 ± 0.68  CG: +3.17 ± 0.80  Time*group interaction NS | |
|  |  |  |  |  |  |  |  | Hip extensor strength (kg) | 1 y: 99/94  (mITT) | Mean change ± SEM:  IG: -0.62 ± 0.57  CG: +1.37 ± 0.66  Time*group interaction NS | |
|  |  |  |  |  |  |  |  |  | 2 y:  93/88  (mITT) | Mean change ± SEM:  IG: +0.12 ± 0.65 CG: +1.34 ± 0.64  Time*group interaction NS | |
|  |  |  |  |  |  |  |  | Hib abductor strength (kg) | 1 y: 99/94  (mITT) | Mean change ± SEM:  IG: +0.67 ± 0.41  CG: +0.85 ± 0.48  Time*group interaction NS | |
|  |  |  |  |  |  |  |  |  | 2 y:  93/88  (mITT) | Mean change ± SEM:  IG: +1.46 ± 0.41  CG: +1.28 ± 0.58  Time*group interaction NS | |
|  |  |  |  |  |  |  |  | Hip flexor strength (kg) | 1 y: 99/94  (mITT) | Mean change ± SEM:  IG: +1.51 ± 0.44  CG: +2.20 ± 0.49  Time*group interaction NS | |
|  |  |  |  |  |  |  |  |  | 2 y:  93/88  (mITT) | Mean change ± SEM:  IG: +3.55 ± 0.57  CG: +3.67 ± 0.55  Time*group interaction NS | |
|  |  |  |  |  |  |  |  | Hip adductor strength (kg) | 1 y: 99/94  (mITT) | Mean change ± SEM:  IG: -2.06 ± 0.53  CG: -1.59 ± 0.61  Time*group interaction NS | |
|  |  |  |  |  |  |  |  |  | 2 y:  93/88  (mITT) | Mean change ± SEM:  IG: -1.53 ± 0.53  CG: -2.05 ± 0.68  Time*group interaction NS | |
| In the context of (concomitant) physical exercise | | | | | | | | | | |  |
| Arnarson et al. 2013 (12), Iceland  Same RCT as Ramel et al. 2013 (13) | Community-dwelling older men and women aged ≥65 y;  without major orthopaedic disease or musculoskeletal disorders; 58% female; mean age: 74 ± 6 y (range: 65-91 y); mean BMI: 29 ± 5 kg/m^2^ (82% ≥25 kg/m^2^); race NR | Whey protein. Drink containing 20 g of whey protein isolate (+ 20 g of CHO), consumed after WBR (so only on training days), [A]  WBR, 3 times/wk, 3 sets of 6-8 reps, 75-80% 1RM | CHO placebo. Drink containing 40 g of CHO, consumed after WBR (so only on training days)  WBR, 3 times/wk, 3 sets of 6-8 reps, 75-80% 1RM | NR | Baseline:  IG: 1.00 ± 0.26, CG: 0.92 ± 0.30  Follow-up:  IG: 1.06 ± 0.23, CG 0.89 ± 0.23 | 12 wk | High | Quadriceps strength (N) | 75/66 | Mean change ± SD:  IG: +56.5 ± 59.4 CG: +53.8 ± 51.8  P=0.776 | |
| Chalé et al. 2013 (15), USA | Community-dwelling, mobility-limited, sedentary older adults aged 70-85 y with habitual protein intake ≥0.8 g/kg BW/d; 59% female; mean age: 78 ± 4 y; mean BMI: 27 ± 3 kg/m^2^; race: White (70%), Black (16%), Asian (4%), other or unknown (10%) | Whey protein concentrate: 20 g consumed after breakfast and 20 g consumed after evening meal each day (total: 40 g protein/d). On training days, one serving was consumed immediately after WBR, [A]  WBR, 3 times/wk, 10 reps, 80% 1RM | CHO placebo. Isocaloric amount consumed after breakfast and after evening meal each day. On training days, one serving was consumed immediately after WBR.  WBR, 3 times/wk, 10 reps, 80% 1RM | IG: 72.1 ± 29.3%, CG: 82.3 ± 21.9% | Baseline: IG: 0.97, CG: 0.98  Follow-up:  NR | 6 mo | Some concerns | Double leg press strength, 1RM^§^ (N) | 42/38  (ITT) | MD (95%-CI):  +58 (-87 to +202)  NS | |
|  |  |  |  |  |  |  |  | Knee extension, 1RM, right^§^ (N) | 42/38  (ITT) | MD (95%-CI):  +10 (-31 to +52)  NS | |
|  |  |  |  |  |  |  |  | Knee extension, 1RM, left^§^ (N) | 42/38  (ITT) | MD (95%-CI):  +37 (-37 to +111)  NS | |
|  |  |  |  |  |  |  |  | Double leg press peak power, 40% 1RM^§^ (W) | 42/38  (ITT) | MD (95%-CI):  +53 (-33 to +140)  NS | |
|  |  |  |  |  |  |  |  | Knee extension peak power, 40% 1RM, right^§^ (W) | 42/38  (ITT) | MD (95%-CI):  +15 (+1 to +29)  **P <0.05** | |
|  |  |  |  |  |  |  |  | Knee extension peak power, 40% 1RM, left^§^ (W) | 42/38  (ITT) | MD (95%-CI):  +15 (+1 to +29)  **P <0.05** | |
|  |  |  |  |  |  |  |  | Double leg press peak power, 70% 1RM^§^ (W) | 42/38  (ITT) | MD (95%-CI):  +61 (-61 to +137)  NS | |
|  |  |  |  |  |  |  |  | Knee extension peak power, 70% 1RM, right^§^ (W) | 42/38  (ITT) | MD (95%-CI):  +27 (+9 to +45)  **P <0.05** | |
|  |  |  |  |  |  |  |  | Knee extension peak power, 70% 1RM, left^§^ (W) | 42/38  (ITT) | MD (95%-CI):  +30 (+12 to +47)  **P <0.05** | |
| Mitchell et al. 2015 (18), Canada | Generally healthy older men; free of musculoskeletal or metabolic disorders; recreationally active; mean age: 74 ± 5 y; mean BMI: 27 ± 3 kg/m^2^; race NR | Milk protein. 500-ml of chocolate milk containing 14 g of protein, consumed <15 min after WBR on training days and with breakfast on non-training days, [B]  WBR, 3 times/wk, 3-4 sets, 75-85% 1RM | CHO placebo. 500-ml drink with isocaloric amount of CHO, consumed <15 min after WBR on training days and with breakfast on non-training days  WBR, 3 times/wk, 3-4 sets, 75-85% 1RM | NR | Baseline:  NR  Follow-up:  NR | 12 wk | High | Knee extension isometric MVC (Nm) | 16 (total) | Mean % change ± SD:  IG: +29.5 ± 17.8  CG: +25.6 ± 49.7  Time*group interaction NS | |
|  |  |  |  |  |  |  |  | Leg press, 1RM (kg) | 16 (total) | Mean % change ± SD:  IG: +52.1 ± 43.1  CG: +35.9 ± 47.6  Time*group interaction NS | |
|  |  |  |  |  |  |  |  | Leg extension, 1RM (kg) | 16 (total) | Mean % change ± SD:  IG: +52.1 ± 43.1  CG: +35.9 ± 47.6  Time*group interaction NS | |
|  |  |  |  |  |  |  |  | Chest press, 1RM (kg) | 16 (total) | Mean % change ± SD:  IG: +9.8 ± 6.9  CG: +4.9 ± 10.7  Time*group interaction NS | |
| Nabuco et al. 2018 (19), Brazil  Same RCT as Nabuco et al. 2019a (20) and Nabuco et al. 2019b (21) | Older women aged ≥60 y, physically independent, free from cardiac or orthopaedic dysfunction; mean age: 67 ± 7 y; mean BMI: 25 ± 5 kg/m^2^; race NR | Whey protein. 35 g of hydrolysed whey protein supplement containing 27.1 g protein (+ 5.2 g CHO), mixed with non-caloric drink. IG1: protein before and placebo after WBR; IG2: placebo before and protein after WBR (so only on training days), [A]  WBR, 3 times/wk, 3 sets of 8-12 reps | CHO placebo containing 0.3 g protein and 33.3 g CHO, mixed with non-caloric drink; one before and one after WBR (so only on training days)  WBR, 3 times/wk, 3 sets of 8-12 reps | NR | Baseline:  IG1: 0.92 ± 0.20, IG2: 0.94 ± 0.36, CG: 0.95 ± 0.27  Follow-up:  IG1: 1.38 ± 0.26, IG2: 1.49 ± 0.46, CG: 1.0 ± 0.25 | 12 wk | Some concerns | Chest press, 1RM (kg) | 22/21/23 (mITT) | Mean % change ± SD:  IG1: +5.6 ± 1.7* IG2: +5.9 ± 1.6*  CG: +4.5 ± 1.2  *** P<0.05 compared to CG** | |
|  |  |  |  |  |  |  |  | Knee extension, 1RM (kg) | 22/21/23  (mITT) | Mean % change ± SD:  IG1: +9.2 ± 2.5* IG2: +8.8 ± 2.2*  CG: +7.5 ± 1.0  *** P<0.05 compared to CG** | |
|  |  |  |  |  |  |  |  | Preacher curl, 1RM (kg) | 22/21/23  (mITT) | Mean % change ± SD:  IG1: +11.3 ± 5.7 IG2: +12.4 ± 6.6  CG: +10.5 ± 5.3  P for time*group interaction=0.376 | |
|  |  |  |  |  |  |  |  | Total strength^c^ (kg) | 22/21/23  (mITT) | Mean % change ± SD:  IG1: +8.1 ± 1.6* IG2: +8.3 ± 2.3*  CG: +7.0 ± 2.7  *** P<0.05 compared to CG** | |
| Nabuco et al. 2019c (22), Brazil | Older women aged ≥60 y with sarcopenic obesity (body fat mass ≥35% and ALST <15.02 kg); physically independent; free from cardiac or orthopaedic dysfunction; mean age: 69 ± 4 y; mean BMI: 27 ± 3 kg/m^2^; race NR | Whey protein. 35 g of hydrolysed whey protein supplement, mixed with non-caloric drink, ingested after WBR (so only on training days), [A]  WBR, 3 times/wk, 3 sets of 8-12 reps | CHO placebo. Isocaloric amount of maltodextrin mixed with non-caloric drink, ingested after WBR (so only on training days)  WBR, 3 times/wk, 3 sets of 8-12 reps | NR | Baseline  IG: 0.93 ± 0.36, CG: 0.97 ± 0.28  Follow-up:  IG: 1.0 ± 0.23, CG: 1.0 ± 0.19 (without supplementation) | 12 wk | Some concerns | Knee extension, 1RM (kg) | 13/13  (ITT) | Mean % change:  IG: +6.3  CG: +4.8  P for time*group interaction=0.347 | |
|  |  |  |  |  |  |  |  | Chest press, 1RM (kg) | 13/13  (ITT) | Mean % change:  IG: +4.7  CG: +4.5  P for time*group interaction=0.696 | |
|  |  |  |  |  |  |  |  | Preacher curl, 1RM (kg) | 13/13  (ITT) | Mean % change:  IG: +12.4  CG: +10.2  P for time*group interaction=0.247 | |
|  |  |  |  |  |  |  |  | Total strength^c^ (kg) | 13/13  (ITT) | Mean % change:  IG: +6.8  CG: +5.7  P for time*group interaction=0.248 | |
| Sugihara Junior et al. 2018 (17), Brazil  Same RCT as Fernandes et al. 2018 (16) | Older women aged ≥60 y; physically independent; free from cardiac or orthopaedic dysfunction; protein intake <1.2 g/kg BW; mean age: 68 ± 4 y; mean BMI: 26 ± 3 kg/m^2^; race NR | Whey protein. 35 g of hydrolysed whey protein containing 27.1 g of protein, dissolved in 200 ml sugar-free soft drink, ingested after WBR (so only on training days), [A]  WBR, 3 times/wk, 3 sets of 8-12RM | CHO placebo. 35 g of maltodextrin, dissolved in 200 ml sugar-free soft drink, ingested after WBR (so only on training days)  WBR, 3 times/wk, 3 sets of 8-12RM | NR | Baseline:  IG: 0.85 ± 0.1, CG: 0.81 ± 0.1 Follow-up:  IG: 1.4 ± 0.1, CG: 0.87 ± 0.1 | 12 wk | High | Chest press strength, 1RM (kg) | 15/16 | Mean % change:  IG: +6.3  CG: +2.7  **P for time*group interaction<0.01** | |
|  |  |  |  |  |  |  |  | Knee extension strength, 1RM (kg) | 15/16 | Mean % change:  IG: +8.5  CG: +4.4  **P for time*group interaction=0.01** | |
|  |  |  |  |  |  |  |  | Preacher curl strength, 1RM (kg) | 15/16 | Mean % change:  IG: +14.0  CG: +10.5  P for time*group interaction=0.07 | |
|  |  |  |  |  |  |  |  | Total strength, 1RM (kg) | 15/16 | Mean % change:  IG: +8.7  CG: +4.9  **P for time*group interaction<0.01** | |
|  |  |  |  |  |  |  |  | Lower limb muscle quality index^d^ | 15/16 | Mean % change:  IG: +4.4  CG: +4.5  P for time*group interaction=0.68 | |
|  |  |  |  |  |  |  |  | Upper limb muscle quality index^e^ | 15/16 | Mean % change:  IG: +11.5  CG: +7.4  P for time*group interaction=0.16 | |
|  |  |  |  |  |  |  |  | Total muscle quality index^f^ | 15/16 | Mean % change:  IG: +2.9  CG: +1.5  P for time*group interaction=0.58 | |
| Ten Haaf et al. 2019 (23), The Netherlands | Physically active older adults aged ≥65 y with habitual protein intake <1.0 g/kg BW; without type 2 diabetes, cancer, renal insufficiency (eGFR <30) or COPD; median age: 69 (range 67-73) y; mean BMI: 27 ± 3 kg/m^2^; Caucasian (except for 1 Asian participant) | Milk-protein concentrate. 250-ml protein drink containing 15.5 g protein (+ 1.1 g fat + 14.5 g lactose) consumed twice a day (total: 31 g protein/d), [A]  Training (walking) for the Nijmegen Four Days Marches | CHO placebo. 250-ml isocaloric drink containing 1.1 g protein (+ 5.2 g fat + 36 g CHO) consumed twice daily  Training (walking) for the Nijmegen Four Days Marches | IG: 96 ± 3%, CG: 95 ± 3% | Baseline:  IG: 0.86 ± 0.23, CG: 0.92 ± 0.24  Follow-up:  IG: 0.92 ± 0.27, CG: 0.97 ± 0.23 (without supplementation) | 12 wk | Some concerns | Handgrip strength^§^ (kg) | 58/56  (PP) | Mean change ± SD:  IG: 0 ± 4  CG: +1 ± 4  P for time*group interaction=0.24 | |
|  |  |  |  |  |  |  |  | Quadriceps MVC^§^ (N) | 56 (total; PP) | Mean change ± SD:  IG: +7.2 ± 71.6  CG: -8.7 ± 63.1  P for time*group interaction=0.38 | |
|  |  |  |  |  |  |  |  | Maximal rate of force rise, quadriceps^§^ (%/ms) | 44 (total; PP) | Mean change ± SD:  IG: -0.06 ±0.09  CG: -0.03 ± 0.11  P for time*group interaction=0.38 | |
|  |  |  |  |  |  |  |  | Early relaxation time, quadriceps^§^ (ms) | 33 (total; PP) | Mean change ± SD:  IG: -0.27 ± 3.05  CG: +0.29 ± 2.7  P for time*group interaction=0.58 | |
|  |  |  |  |  |  |  |  | Half relaxation time, quadriceps^§^ (ms) | 22 (total; PP) | Mean change ± SD:  IG: +1.1 ± 8.5  CG: +1.5 ± 4.5  P for time*group interaction=0.87 | |
|  |  |  |  |  |  |  |  | Fatigue^§^ (%) | 30 (total; PP) | Mean change ± SD:  IG: -0.6 ± 8.5  CG: +1.1 ± 7.4 P for time*group interaction=0.57 | |
| Thomson et al. 2016 (24), Australia | Older adults aged 50-79 y and BMI of 20-35 kg/m^2^ who are physically active (but not engaged in formal exercise); without major chronic diseases (e.g. diabetes, cancer, metabolic disease, cardiac abnormalities, musculoskeletal injury); 55% female; mean age: 62 ± 7 y; mean BMI: 28 ± 4 kg/m^2^; race NR | High-protein diet. Individualised diet providing 1.0 g/kg BW/d + ~27 g dairy-based protein drink each day (IG1); Individualised diet providing 1.0 g/kg BW/d + ~27 g soy protein drink each day (IG2), [B]  WBR, 3 times/wk, 1-3 sets of 8-20 reps, 8RM | CHO placebo. Individualised diet providing 1.0 g/kg BW/d + ~27 g CHO drink each day  WBR, 3 times/wk, 1-3 sets of 8-20 reps, 8RM | IG1: 97%, IG2: 98%, CG: 98% | Baseline:  IG1: 1.06 ± 0.10, IG2: 1.08 ± 0.09, CG: 1.02 ± 0.05  Follow-up:  IG1: 1.42 ± 0.14, IG2: 1.45 ± 0.14, CG: 1.08 ± 0.05 | 12 wk | High | Knee extensor strength (Nm) | 34/26/23 (PP^g^) | Mean change ± SD:  IG1: +25.5 ± 22.1  IG2: +18.4 ± 18.6  CG: +30.5 ± 24.8 Between-group difference NS; P=0.08 for change in IG2 compared to CG | |
|  |  |  |  |  |  |  |  | Handgrip strength (kg) | 34/26/23 (PP^g^) | Mean change ± SD:  IG1: +1.0 ± 3.1  IG2: +1.6 ± 3.1  CG: +2.0 ± 3.9 Between-group difference NS | |
|  |  |  |  |  |  |  |  | Leg press, 8RM (kg) | 34/26/23 (PP^g^) | Mean (%) change ± SD:  IG1: +65.2 ± 30.3 (+136.8 ± 88.2%)  IG2: +47.4 ± 34.1 (+64.8 ± 35.2%)  CG: +66.3 ± 25.4 (+135.0 ± 62.0%) **Significant difference between IG2 and CG**, but not between IG1 and CG | |
|  |  |  |  |  |  |  |  | Chest press, 8RM (kg) | 34/26/23 (PP^g^) | Mean change ± SD:  IG1: +17.4 ± 6.7  IG2: +17.4 ± 11.1  CG: +13.3 ± 4.5 Between-group difference NS | |
|  |  |  |  |  |  |  |  | Knee extension strength, 8RM (kg) | 34/26/23 (PP^g^) | Mean change ± SD:  IG1: +29.4 ± 14.1  IG2: +23.0 ± 11.7  CG: +25.6 ± 10.5 Between-group difference NS | |
|  |  |  |  |  |  |  |  | Lat pull down, 8RM (kg) | 34/26/23 (PP^g^) | Mean (%) change ± SD:  IG1: +10.1 ± 5.3 (+24.6 ± 12.2%)  IG2: +11.3 ± 6.4 (+28.2 ± 11.7%)  CG: +12.5 ± 5.6 (+35.1 ± 17.0%) Between-group difference in absolute change NS; **significant difference in % change between IG1 and CG**, but not between IG2 and CG | |
|  |  |  |  |  |  |  |  | Leg curl, 8RM (kg) | 34/26/23 (PP^g^) | Mean change ± SD:  IG1: +13.9 ± 9.0  IG2: +12.0 ± 6.3  CG: +11.3 ± 5.5 Between-group difference NS (for both absolute and % change) | |
|  |  |  |  |  |  |  |  | Total 8RM (kg) | 34/26/23 (PP^g^) | Mean (%) change ± SD:  IG1: +131.3 ± 54.2 (+92.1 ± 40.8%)  IG2: +102.1 ± 50.7 (+63.0 ± 23.8%)  CG: +126.1 ± 41.3 (+92.3 ± 35.4%)  **Significant difference between IG2 and CG**, but not between IG1 and CG | |

Abbreviations: BMI, body mass index; BW, body weight; CI, confidence interval; CG, control group; CHO, carbohydrates; CKD, chronic kidney disease; COPD, chronic obstructive pulmonary disease; CVD, cardiovascular disease; E%, percentage of energy intake; EAA, essential amino acids; eGFR, estimated glomerular filtration rate; IG, intervention group; ITT, intention-to-treat analysis or modified intention-to-treat analysis (mITT: only those with missing outcome data were excluded from the analytic sample); MD, mean difference (i.e. difference in within-group change); MNA, Mini Nutritional Assessment; mo, months; ms, millisecond; MVC, maximal voluntary contraction; *n*, number; N, Newton; Nm, Newton-metre; NR, not reported; NS, not significant; PP, per-protocol analysis; reps, repetitions; RM, repetition maximum; SD, standard deviation; SEM, standard error of the mean; WBR, whole-body resistance training; wk, weeks; y, years.

Statistically significant effects are shown in bold.

^§^ Sufficient statistical power to detect an effect is to be expected, based on the sample size calculation.

^a^ ‘Protein type’ indicates the way in which a higher protein intake was achieved and is categorized into ‘pure’ protein or amino acids (or essential amino acids) (A), specific product with a high protein content (B) or high-protein diets (C).

^b^ Risk of bias was assessed using the RoB 2 Cochrane collaboration tool and scored as ‘low’ (L), ‘some concerns’ (SC) or ‘high’ (H).

^c^ Total strength was calculated as the sum of chest press, knee extension and preacher curl strength (kg).

^d^ Lower limb muscle quality index was calculated as knee extension strength divided by lower limb lean soft tissue.

^e^ Upper limb muscle quality index was calculated as preacher curl strength divided by upper limb lean soft tissue.

^f^ Total muscle quality index was calculated as total strength divided by skeletal muscle mass.

^g^ Intention-to-treat analysis demonstrated a similar pattern to the per-protocol analysis (data not shown).

Supplemental Table 3

Results from randomized controlled trials on the effect of increased protein intake on physical performance in older adults, grouped according to whether or not the protein intervention was carried out in the context of (concomitant) physical exercise

| Study reference, country | Study population | Exposure (IG), [protein type^a^] | Comparison (CG) | Compliance | Total protein intake (g/kg BW/d) | Study duration | Risk of bias^b^ | Outcome | Analytic *n* IG/CG | Results | |
| --- | --- | --- | --- | --- | --- | --- | --- | --- | --- | --- | --- |
| Not in the context of (concomitant) physical exercise | | | | | | | | | | |  |
| Bhasin et al. 2018 (1) USA | Community-dwelling older men aged ≥65 y with moderate physical function limitations and with habitual protein intake ≤0.83 g/kg BW/d; mean age: 73 ± 6 y; mean BMI: 30.3 ± 4.9 kg/m^2^; race NR | Individualised diets providing 0.7 g protein/kg BW/d with additional discretionary foods (0.1 g protein/kg BW) and protein supplements (0.5 g/kg BW; 50:50 casein:whey mix) to achieve a total of 1.3 g/kg BW/d, [A,B] | Individualised diets providing 0.7 g protein/kg BW/d with additional discretionary foods (0.1 g protein/kg BW) and placebo supplements (0.5 g CHO/kg BW) to achieve a total of 0.8 g/kg BW/d | Foods (4-6 mo):  IG: 77.1 ± 13%, CG: 74.5 ± 23.2%  Supplements (4-6 mo):  IG: 91.2 ± 12.4%, CG: 92.6 ± 11.0% | Baseline:  IG: 0.72 ± 0.11, CG: 0.69 ± 0.15  Follow-up (1-3 mo):  IG: 1.18 ± 0.15, CG: 0.84 ± 0.07  Follow-up (4-6 mo): IG: 1.17 ± 0.13, CG: 0.81 ± 0.10 | 6 mo | Some concerns | Gait speed, 6-min at fast pace (m) | 40/37  (mITT) | MD (95%-CI):  -0.89 (-32.1 to +30.4)  P=0.96 | |
|  |  |  |  |  |  |  |  | Gait speed, 50-m at fast pace, loaded (m/s) | 34/32  (mITT) | MD (95%-CI):  -0.01 (-0.10 to +0.07)  P=0.81 | |
|  |  |  |  |  |  |  |  | Stair climb power, 12 steps, unloaded at fast pace (W) | 36/33  (mITT) | MD (95%-CI):  -28.3 (-59.8 to +3.2)  P=0.08 | |
|  |  |  |  |  |  |  |  | Stair climb power, 12 steps, loaded at fast pace (W) | 33/33  (mITT) | MD (95%-CI):  -11.5 (-46.5 to +23.5)  P=0.52 | |
|  |  |  |  |  |  |  |  | Perceived physical function (SF-36) | 42/40 (mITT) | MD (95%-CI):  -1.98 (-9.11 to +5.15)  P=0.58 | |
| Ispoglou et al. 2016 (3), UK | Community-dwelling older men and women aged 65-75 y; good health, without major chronic diseases (e.g. diabetes, vascular disease, hypertension); 44% female; mean age: 72 ± 3 y; mean BMI: 27 ± 4 y; race: NR | EAA. Standard EAA mixture with 20% leucine (IG1) and with 40% leucine (IG2), ingested at breakfast and dinner (total: 0.21 g/kg BW/d = ~11-21 g/d), [A] | CHO placebo. Isocaloric amount of lactose, ingested at breakfast and dinner | 74-83% | Baseline: 0.95-1.10 g/kg BW/d  Follow-up: 1.02-1.08 g/kg BW/d (without supplementation) | 12 wk | High | Gait speed, 6-min (m) | 8/8/9  (PP) | Mean % change ± SD:  IG1: +8.8 ± 10.0  IG2: +5.8 ± 6.6  CG: +1.4 ± 4.5  P=0.132 | |
|  |  |  |  |  |  |  |  | 30-s chair-stand test (n) | 8/8/9  (PP) | Mean % change ± SD:  IG1: +11.0 ± 11.5  IG2: +13.2 ± 16.0  CG: +4.7 ± 15.7  P=0.470 | |
| Mitchell et al. 2017 (5), New Zealand | Community-dwelling older men aged >70 y with BMI of 18-35 kg/m^2^; able to perform ADLs without mobility aids; without major chronic diseases (e.g. cancer, diabetes, thyroid diseases); mean age: 74 y (range: 70-81 y); mean BMI: 28 ± 4 kg/m^2^; race NR | High-protein diet (2*RDA = 1.6 g/kg BW/d), omnivorous, 28-31 E% fat, [C] | Low-protein diet (1*RDA = 0.8 g/kg BW/d), omnivorous, 28-31 E% fat; difference made up of CHO | IG: 97.5%, CG: 98.9% | Baseline:  IG: 1.1 ± 0.3, CG: 1.2 ± 0.4  Follow-up:  IG: 1.7 ± 0.1, CG: 0.9 ± 0.1 | 10 wk | High | SPPB (score) | 14/15  (PP) | P for time*group interaction=0.185 | |
|  |  |  |  |  |  |  |  | TUG (s) | 14/15  (PP) | P for time*group interaction=0.313 | |
| Ottestad et al. 2017 (6), Norway | Community-dwelling older adults aged ≥70 y; relatively healthy (no diabetes, CVD, cancer, COPD, CKD); not malnourished; with reduced muscle strength or performance; 67% female; mean age: 77 ± 5 y; mean BMI: 27 ± 5 kg/m^2^; race NR | Protein-enriched milk. 400 ml drink containing 20 g protein (80:20 casein:whey mix, consumed twice a day (total: 40 g protein/d), [B] | CHO placebo. 400 ml drink containing an isocaloric amount of CHO, consumed twice a day | IG: 97.8 ± 3.8%, CG: 96.8 ± 5.7% | Baseline:  IG: 1.0 ± 0.3, CG: 1.0 ± 0.3  Follow-up:  IG: 1.4 ± 0.5, CG: 0.9 ± 0.4 | 12 wk | High | Chair rise time, 5x (s) | 16/17  (mITT) | Mean change (95%-CI):  IG: -0.2 (-1.2 to +0.7)  CG: -0.3 (-1.2 to +5.4)  P=0.83 | |
|  |  |  |  |  |  |  |  | Stair climb time, 20 steps, unloaded (s) | 16/15  (mITT) | Mean change (95%-CI):  IG: -0.4 (-0.8 to -0.1)  CG: 0.0 (-0.7 to +0.7)  P=0.22 | |
|  |  |  |  |  |  |  |  | Stair climb time, 20 steps, 10-kg loaded (s) | 16/15  (mITT) | Mean change (95%-CI):  IG: -0.2 (-0.7 to +0.3)  CG: -0.2 (-1.0 to +0.6)  P=0.94 | |
| Park et al. 2018 (7), Korea | Community-dwelling (pre-)frail older adults aged 70-85 y at risk of malnutrition (MNA ≤23.5); no kidney or liver failure; able to walk; 65% female; mean age: 77 ± 4 y; mean BMI: 24 ± 3 kg/m^2^; Asian | Whey protein. Multiple 10-g packs of protein powder (9.3 g whey protein/pack), dissolved in 340 ml tea, were provided in addition to habitual protein intake up to 1.2 g/kg BW/d (IG1) and 1.5 g/kg BW/d (IG2), [A] | CHO placebo. Multiple 10-g packs of CHO powder (9.3 g maltodextrin per/pack), dissolved in 340 ml tea, were provided in addition to habitual protein intake up to 0.8 (CG) or 1.2 (IG1) g/kg BW/d  (CG were given only CHO powder, IGs were given a combination of protein and CHO powder.) | IG1: 98%, IG2: 96%, CG: 97% | Baseline:  IG1: 0.77 ± 0.24, IG2: 0.80 ± 0.21, CG: 0.84 ± 0.28  Follow-up:  IG1: 1.18 ± 0.23, IG2: 1.37 ± 0.26, CG: 0.90 ± 0.38 | 12 wk | Some concerns | SPPB (score) | 40/40/40  (ITT) | P for time*group interaction=0.365 | |
|  |  |  |  |  |  |  |  | Gait speed, 4-m (m/s) | 40/40/40  (ITT) | **P for time*group interaction=0.007**  (faster gait speed at 12 wk in IG2, but not IG1, than in CG) | |
|  |  |  |  |  |  |  |  | Standing balance | 40/40/40  (ITT) | P for time*group interaction=0.319 | |
|  |  |  |  |  |  |  |  | Chair rise time, 5x (s) | 40/40/40  (ITT) | P for time*group interaction=0.881 | |
|  |  |  |  |  |  |  |  | TUG (s) | 40/40/40  (ITT) | P for time*group interaction=0.207 | |
| Zhu et al. 2015 (9), Australia  Same RCT as Hodgson et al. 2012 (10) and Zhu et al. 2011 (11) | Community-dwelling older women aged 70-80 y with habitual protein intake <1.5 g/kg BW/d; without metabolic bone disease, osteoporotic fracture, diabetes, hepatic or renal insufficiency; mean age: 74 ± 3 y; mean BMI: 27 ± 4 kg/m^2^; Caucasian | Whey protein isolate. 250 ml skim milk-based high-protein supplement drink containing 30 g of whey protein + calcium, [A] | CHO placebo. 250 ml skim milk-based supplement drink containing 2.1 g of protein and isocaloric amount of maltodextrin + calcium | 87.1% in IG and 80.8% in CG (P=0.03) | Baseline:  IG: 1.2 ± 0.3, CG: 1.1 ± 0.3 Follow-up (1 y):  IG: 1.4 ± 0.4, CG: 1.1 ± 0.3  Follow-up (2 y): IG: 1.4 ± 0.4, CG: 1.1 ± 0.4 | 1 y and 2 y | Some concerns | TUG (s) | 1 y: 99/94  (mITT)  2 y:  93/88  (mITT) | Mean change ± SEM:  IG: -0.14 ± 0.13  CG: -0.17 ± 0.15  Time*group interaction NS  Mean change ± SEM:  IG: -0.46 ± 0.12 CG: -0.55 ± 0.12  Time*group interaction NS | |
| In the context of (concomitant) physical exercise | | | | | | | | | | |  |
| Arnarson et al. 2013 (12), Iceland  Same RCT as Ramel et al. 2013 (13) | Community-dwelling older men and women aged ≥65 y;  without major orthopaedic disease or musculoskeletal disorders; 58% female; mean age: 74 ± 6 y (range: 65-91 y); mean BMI: 29 ± 5 kg/m^2^ (82% ≥25 kg/m^2^); race NR | Whey protein. Drink containing 20 g of whey protein isolate (+ 20 g of CHO), consumed after WBR (so only on training days), [A]  WBR, 3 times/wk, 3 sets of 6-8 reps, 75-80% 1RM | CHO placebo. Drink containing 40 g of CHO, consumed after WBR (so only on training days)  WBR, 3 times/wk, 3 sets of 6-8 reps, 75-80% 1RM | NR | Baseline:  IG: 1.00 ± 0.26, CG: 0.92 ± 0.30  Follow-up:  IG: 1.06 ± 0.23, CG 0.89 ± 0.23 | 12 wk | High | Gait speed, 6-min (m) | 75/66 | Mean change ± SD:  IG: +35.1 ± 38.0 CG: +39.9 ± 75.9  P=0.726 | |
|  |  |  |  |  |  |  |  | TUG (s) | 75/66 | Mean change ± SD:  IG: -0.6 ± 1.7 CG: -0.5 ± 0.8  P=0.151 | |
|  |  |  |  |  |  |  |  |  |  |  |  |
|  |  |  |  |  |  |  |  |  |  |  |  |
|  |  |  |  |  |  |  |  |  |  |  |  |
|  |  |  |  |  |  |  |  |  |  |  |  |
| Chalé et al. 2013 (15), USA | Community-dwelling, mobility-limited, sedentary older adults aged 70-85 y with habitual protein intake ≥0.8 g/kg BW/d; 59% female; mean age: 78 ± 4 y; mean BMI: 27 ± 3 kg/m^2^; race: White (70%), Black (16%), Asian (4%), other or unknown (10%) | Whey protein concentrate: 20 g consumed after breakfast and 20 g consumed after evening meal each day (total: 40 g protein/d). On training days, one serving was consumed immediately after WBR, [A]  WBR, 3 times/wk, 10 reps, 80% 1RM | CHO placebo. Isocaloric amount consumed after breakfast and after evening meal each day. On training days, one serving was consumed immediately after WBR.  WBR, 3 times/wk, 10 reps, 80% 1RM | IG: 72.1 ± 29.3%, CG: 82.3 ± 21.9% | Baseline: IG: 0.97, CG: 0.98  Follow-up:  NR | 6 mo | Some concerns | Gait speed, 400-m^§^ (m/s) | 42/38  (ITT) | MD (95%-CI):  +0.08 (-0.02 to +0.19)  NS | |
|  |  |  |  |  |  |  |  | Stair climb time^§^ (s) | 42/38  (ITT) | MD (95%-CI):  +0.3 (-1.1 to +1.8)  NS | |
|  |  |  |  |  |  |  |  | Chair rise time, 10x^§^ (s) | 42/38  (ITT) | MD (95%-CI):  -1.9 (-5.2 to +1.4)  NS | |
|  |  |  |  |  |  |  |  | SPPB^§^ (score) | 42/38  (ITT) | MD (95%-CI):  +0.21 (-0.41 to +0.83)  NS | |
| Nabuco et al. 2018 (19), Brazil  Same RCT as Nabuco et al. 2019a (20) and Nabuco et al. 2019b (21) | Older women aged ≥60 y, physically independent, free from cardiac or orthopaedic dysfunction; mean age: 67 ± 7 y; mean BMI: 25 ± 5 kg/m^2^; race NR | Whey protein. 35 g of hydrolysed whey protein supplement containing 27.1 g protein (+ 5.2 g CHO), mixed with non-caloric drink. IG1: whey protein before and placebo after WBR; IG2: placebo before and whey protein after WBR (so only on training days), [A]  WBR, 3 times/wk, 3 sets of 8-12 reps | CHO placebo containing 0.3 g protein and 33.3 g CHO, mixed with non-caloric drink; one before and one after WBR (so only on training days)  WBR, 3 times/wk, 3 sets of 8-12 reps | NR | Baseline:  IG1: 0.92 ± 0.20, IG2: 0.94 ± 0.36, CG: 0.95 ± 0.27  Follow-up:  IG1: 1.38 ± 0.26, IG2: 1.49 ± 0.46, CG: 1.0 ± 0.25 | 12 wk | Some concerns | Gait speed, 10-m at fast pace (s) | 22/21/23  (mITT) | Mean % change ± SD:  IG1: -10.8 ± 11.3* IG2: -11.8 ± 8.6*  CG: -4.3 ± 8.4  *** P<0.05 compared to CG** | |
|  |  |  |  |  |  |  |  | Chair rise time, 5x (s) | 22/21/23  (mITT) | Mean % change ± SD:  IG1: -10.0 ± 12.4 IG2: -10.1 ± 5.4  CG: -5.7 ± 7.6  P for time*group interaction=0.176 | |
| Nabuco et al. 2019c (22), Brazil | Older women aged ≥60 y with sarcopenic obesity (body fat mass ≥35% and ALST <15.02 kg); physically independent; free from cardiac or orthopaedic dysfunction; mean age: 69 ± 4 y; mean BMI: 27 ± 3 kg/m^2^; race NR | Whey protein. 35 g of hydrolysed whey protein supplement, mixed with non-caloric drink, ingested after WBR (so only on training days), [A]  WBR, 3 times/wk, 3 sets of 8-12 reps | CHO placebo. Isocaloric amount of maltodextrin mixed with non-caloric drink, ingested after WBR (so only on training days)  WBR, 3 times/wk, 3 sets of 8-12 reps | NR | Baseline  IG: 0.93 ± 0.36, CG: 0.97 ± 0.28  Follow-up:  IG: 1.0 ± 0.23, CG: 1.0 ± 0.19 (without supplementation) | 12 wk | Some concerns | Gait speed, 10-m (s) | 13/13  (ITT) | Mean % change:  IG: -6.7  CG: -7.6  P for time*group interaction=0.792 | |
|  |  |  |  |  |  |  |  | Chair rise time, 5x (s) | 13/13  (ITT) | Mean % change:  IG: -11.5  CG: -10.1  P for time*group interaction=0.694 | |
| Ten Haaf et al. 2019 (23), The Netherlands | Physically active older adults aged ≥65 y with habitual protein intake <1.0 g/kg BW; without type 2 diabetes, cancer, renal insufficiency (eGFR <30) or COPD; median age: 69 (range 67-73) y; mean BMI: 27 ± 3 kg/m^2^; Caucasian (except for 1 Asian participant) | Milk-protein concentrate. 250-ml protein drink containing 15.5 g protein (+ 1.1 g fat + 14.5 g lactose) consumed twice a day (total: 31 g protein/d), [A]  Training (walking) for the Nijmegen Four Days Marches | CHO placebo. 250-ml isocaloric drink containing 1.1 g protein (+ 5.2 g fat + 36 g CHO) consumed twice daily  Training (walking) for the Nijmegen Four Days Marches | IG: 96 ± 3%, CG: 95 ± 3% | Baseline:  IG: 0.86 ± 0.23, CG: 0.92 ± 0.24  Follow-up:  IG: 0.92 ± 0.27, CG: 0.97 ± 0.23 (without supplementation) | 12 wk | Some concerns | SPPB^§^ (score) | 58/56  (PP) | Median change (IQR):  IG: 0 (0 to +1)  CG: 0 (0 to 0)  P for time*group interaction=0.73 | |
|  |  |  |  |  |  |  |  | Standing balance^§^ (score) | 58/56  (PP) | Median change (IQR):  IG: 0 (0 to 0) CG: 0 (0 to 0)  P for time*group interaction=1.00 | |
|  |  |  |  |  |  |  |  | Gait speed, 4-m at usual pace^§^ (s) | 58/56  (PP) | Mean change ± SD:  IG: -0.2 ± 0.5  CG: -0.2 ± 0.4 P for time*group interaction=0.95 | |
|  |  |  |  |  |  |  |  | Chair rise time^§^, 5x (s) | 111 (total; PP) | Mean change ± SD:  IG: -0.8 ± 2.2  CG: -0.7 ± 1.9  P for time*group interaction=0.86 | |
|  |  |  |  |  |  |  |  | TUG^§^ (s) | 58/56  (PP) | Mean change ± SD:  IG: -0.4 ± 0.9  CG: -0.5 ± 0.6  P for time*group interaction=0.50 | |
| Thomson et al. 2016 (24), Australia | Older adults aged 50-79 y and BMI of 20-35 kg/m^2^ who are physically active (but not engaged in formal exercise); without major chronic diseases (e.g. diabetes, cancer, metabolic disease, cardiac abnormalities, musculoskeletal injury); 55% female; mean age: 62 ± 7 y; mean BMI: 28 ± 4 kg/m^2^; race NR | High-protein diet. Individualised diet providing 1.0 g/kg BW/d + ~27 g dairy-based protein drink each day (IG1); Individualised diet providing 1.0 g/kg BW/d + ~27 g soy protein drink each day (IG2), [B]  WBR, 3 times/wk, 1-3 sets of 8-20 reps, 8RM | CHO placebo. Individualised diet providing 1.0 g/kg BW/d + ~27 g CHO drink each day  WBR, 3 times/wk, 1-3 sets of 8-20 reps, 8RM | IG1: 97%, IG2: 98%, CG: 98% | Baseline:  IG1: 1.06 ± 0.10, IG2: 1.08 ± 0.09, CG: 1.02 ± 0.05  Follow-up:  IG1: 1.42 ± 0.14, IG2: 1.45 ± 0.14, CG: 1.08 ± 0.05 | 12 wk | High | Gait speed, 6-min (m) | 34/26/23 (PP^c^) | Mean change ± SD:  IG1: +36.5 ± 35.9  IG2: +25.1 ± 35.5  CG: +19.2 ± 54.1 Between-group difference NS | |

Abbreviations: ADL, activities of daily living; BMI, body mass index; BW, body weight; CI, confidence interval; CG, control group; CHO, carbohydrates; CKD, chronic kidney disease; COPD, chronic obstructive pulmonary disease; CVD, cardiovascular disease; E%, percentage of energy intake; EAA, essential amino acids; eGFR, estimated glomerular filtration rate; IG, intervention group; ITT, intention-to-treat analysis or modified intention-to-treat analysis (mITT; only those with missing outcome data were excluded from the analytic sample); m, metres; MD, mean difference (i.e. difference in within-group change); MNA, Mini Nutritional Assessment; mo, months; *n*, number; NR, not reported; NS, not significant; PEDro, Physiotherapy Evidence Database scale; PP, per-protocol analysis; reps, repetitions; RM, repetition maximum; s, seconds; SD, standard deviation; SEM, standard error of the mean; SF-36, 36-item Short Form Healthy Survey, physical functioning domain; SPPB, short physical performance battery; TUG, timed up-and-go; WBR, whole-body resistance training; wk, weeks; y, years.

Statistically significant effects are shown in bold.

^a^ ‘Protein type’ indicates the way in which a higher protein intake was achieved and is categorized into ‘pure’ protein or amino acids (or essential amino acids) (A), specific product with a high protein content (B), or high-protein diets (C).

^b^ Risk of bias was assessed using the RoB 2 Cochrane collaboration tool and scored as ‘low’ (L), ‘some concerns’ (SC), or ‘high’ (H).

^c^ Intention-to-treat analysis demonstrated a similar pattern to the per-protocol analysis (data not shown)

Supplemental Table 4

Results from randomized controlled trials on the effect of increased protein intake on bone health in older adults, grouped according to whether or not the protein intervention was carried out in the context of (concomitant) physical exercise

| Study, country | Study population | Exposure (IG), [protein type^a^] | Comparison (CG) | Compliance | Total protein intake (g/kg BW/d) | Study duration | Risk of bias^b^ | Outcome | Analytic *n* IG/CG | Results | |  |  |
| --- | --- | --- | --- | --- | --- | --- | --- | --- | --- | --- | --- | --- | --- |
| Not in the context of (concomitant) physical exercise | | | | | | | | | | | | |  |
| Ispoglou et al. 2016 (3), UK | Community-dwelling older men and women aged 65-75 y; good health, without major chronic diseases (e.g. diabetes, vascular disease, hypertension); 44% female; mean age: 72 ± 3 y; mean BMI: 27 ± 4 y; race: NR | EAA. Standard EAA mixture with 20% leucine (IG1) and with 40% leucine (IG2), ingested at breakfast and dinner (total: 0.21 g/kg BW/d = ~11-21 g/d), [A] | CHO placebo. Isocaloric amount of lactose, ingested at breakfast and dinner | 74-83% | Baseline: 0.95-1.10 g/kg BW/d  Follow-up: 1.02-1.08 g/kg BW/d (without supplementation) | 12 wk | High | Total BMC (kg; DXA) | 8/8/9  (PP) | | Mean % change ± SD:  IG1: +0.1 ± 1.4  IG2: +0.0 ± 1.0  CG: +0.4 ± 1.0  NS | | |
|  |  |  |  |  |  |  |  | Total BMD (g/cm^2^; DXA) | 8/8/9  (PP) | | Mean % change ± SD:  IG1: +0.2 ± 1.2  IG2: +0.3 ± 1.6  CG: -0.4 ± 1.1  NS | | |
| Kerstetter et al. 2015 (4), USA | Older men (aged >70 y) and women (aged >60 y) with BMI of 19-32 kg/m^2^ and protein intake of 0.6-1.0 g/kg BW; without major chronic diseases (e.g. diabetes, renal disease, inflammatory bowel disease) or cancer within past 18 months; 86% female; mean age: 70 ± 6 y; mean BMI: 26 ± 4 kg/m^2^; Caucasian | Whey protein. 45 g of whey protein isolate (~40 g of protein) + vitamin D (400 IU) + calcium (1200 mg), [A] | CHO placebo.  Isocaloric amount of maltodextrin + vitamin D (400 IU) + calcium (1200 mg) | NR | Baseline:  IG: 1.07 ± 0.03, CG: 1.06 ± 0.03  Follow-up:  IG: 1.30 ± 0.05, CG: 1.05 ± 0.04 | 9 mo and 18 mo | Some concerns | BMD lumbar spine^§^ (g/cm^2^; DXA) | 9 mo: 105/102  (mITT) | LSMD (95%-CI):  -0.001 (-0.012 to +0.010)  NS | |  |  |
|  |  |  |  |  |  |  |  |  | 18 mo: 105/102  (mITT) | LSMD (95%-CI):  +0.002 (-0.011 to +0.014)  NS | |  |  |
|  |  |  |  |  |  |  |  | BMD total hip^§^ (g/cm^2^; DXA) | 9 mo: 106/102  (mITT) | LSMD (95%-CI):  -0.001 (-0.007 to +0.005)  NS | |  |  |
|  |  |  |  |  |  |  |  |  | 18 mo: 106/102  (mITT) | LSMD (95%-CI):  +0.001 (-0.007 to +0.009)  NS | |  |  |
|  |  |  |  |  |  |  |  | BMD femoral neck^§^ (g/cm^2^; DXA) | 9 mo: 106/102  (mITT) | LSMD (95%-CI):  +0.004 (-0.004 to +0.012)  NS | |  |  |
|  |  |  |  |  |  |  |  |  | 18 mo: 106/102  (mITT) | LSMD (95%-CI):  +0.006 (-0.004 to +0.016)  NS | |  |  |
|  |  |  |  |  |  |  |  | BMD lumbar spine^§^ (mg/cm^3^; QCT) | 18 mo: 45/44  (mITT) | LSMD (95%-CI):  +4.151 (-0.169 to +8.470)  NS | |  |  |
|  |  |  |  |  |  |  |  | BMD femoral neck, cortical^§^ (mg/cm^3^; QCT) | 18 mo: 45/44  (mITT) | LSMD (95%-CI):  -0.863 (-50.756 to +49.029)  NS | |  |  |
|  |  |  |  |  |  |  |  | BMD femoral neck, trabecular^§^ (mg/cm^3^; QCT) | 18 mo: 45/44  (mITT) | LSMD (95%-CI):  -0.953 (-5.054 to +3.147)  NS | |  |  |
|  |  |  |  |  |  |  |  | BMD femoral total, cortical^§^ (mg/cm^3^; QCT) | 18 mo: 45/44  (mITT) | LSMD (95%-CI):  +1.926 (-29.754 to +33.606)  NS | |  |  |
|  |  |  |  |  |  |  |  | BMD femoral total, trabecular^§^ (mg/cm^3^; QCT) | 18 mo: 45/44  (mITT) | LSMD (95%-CI):  -0.421 (-3.900 to +3.058)  NS | |  |  |
|  |  |  |  |  |  |  |  | Serum P1NP (nmol/L) | 9 mo: 61/60  (mITT) | **P=0.0007**  (increase in IG compared to CG) | |  |  |
|  |  |  |  |  |  |  |  |  | 18 mo: 61/60  (mITT) | P=0.3952 | |  |  |
|  |  |  |  |  |  |  |  | Serum CTX (ng/L) | 9 mo: 61/60  (mITT) | **P=0.0206** (increase in IG compared to CG) | |  |  |
|  |  |  |  |  |  |  |  |  | 18 mo: 61/60  (mITT) | **P=0.0414**  (increase in IG compared to CG) | |  |  |
|  |  |  |  |  |  |  |  | Serum OC (nmol/L) | 9 mo: 61/60  (mITT) | P=0.3332 | |  |  |
|  |  |  |  |  |  |  |  |  | 18 mo: 61/60  (mITT) | P=0.7747 | |  |  |
| Zhu et al. 2011 (11), Australia  Same RCT as Hodgson et al. 2012 (10) and Zhu et al. 2015 (9) | Community-dwelling older women aged 70-80 y with habitual protein intake <1.5 g/kg BW/d; without metabolic bone disease, osteoporotic fracture, diabetes, hepatic or renal insufficiency; mean age: 74 ± 3 y; mean BMI: 27 ± 4 kg/m^2^; Caucasian | Whey protein isolate. 250 ml skim milk-based high-protein supplement drink containing 30 g of whey protein + calcium, [A] | CHO placebo. 250 ml skim milk-based supplement drink containing 2.1 g of protein and isocaloric amount of maltodextrin + calcium | 87.1% in IG and 80.8% in CG (P=0.03) | Baseline:  IG: 1.2 ± 0.3, CG: 1.1 ± 0.3 Follow-up: IG: 1.4 ± 0.4, CG: 1.1 ± 0.4 | 1 y and 2 y | Some concerns | Total hip aBMD^§^ (mg/cm^2^; DXA) | 1 y:  101/91 (mITT) | Time*group interaction NS | |  |  |
|  |  |  |  |  |  |  |  |  | 2 y:  95/88  (mITT) | Time*group interaction NS | |  |  |
|  |  |  |  |  |  |  |  | Femoral neck aBMD^§^ (mg/cm^2^; DXA) | 1 y:  101/91  (mITT) | Time*group interaction NS | |  |  |
|  |  |  |  |  |  |  |  |  | 2 y:  95/88  (mITT) | Time*group interaction NS | |  |  |
|  |  |  |  |  |  |  |  | Total hip volumetric BMD^§^ (mg/cm^3^; QCT) | 67/66 (2 y; mITT) | Mean change ± SE:  IG: -3.63 ± 1.10  CG: -3.82 ± 1.43  Time*group interaction NS | |  |  |
|  |  |  |  |  |  |  |  | Femoral neck vBMD^§^ (mg/cm^2^; QCT) | 67/66 (2 y; mITT) | Mean change ± SE:  IG: -2.39 ± 1.25  CG: -0.24 ± 1.19  Time*group interaction NS | |  |  |
|  |  |  |  |  |  |  |  | Femoral neck bone CSA^§^ (cm^2^; QCT)^c^ | 67/66 (2 y; mITT) | Mean change ± SE:  IG: -0.04 ± 0.06  CG: -0.03 ± 0.06  Time*group interaction NS | |  |  |
|  |  |  |  |  |  |  |  | Femoral neck buckling ratio^§^ (QCT)^d^ | 67/66 (2 y; mITT) | Mean change ± SE:  IG: +0.04 ± 0.09  CG: +0.07 ± 0.09  Time*group interaction NS | |  |  |
|  |  |  |  |  |  |  |  | Femoral neck polar CSMI^§^ (cm^4^; QCT)^e^ | 67/66 (2 y; mITT) | Mean change ± SE:  IG: -0.06 ± 0.07  CG: -0.06 ± 0.08  Time*group interaction NS | |  |  |
| In the context of (concomitant) physical exercise | | | | | | | | | | | | |  |
| Fernandes et al. 2018 (16), Brazil  Same RCT as Sugihara Junior et al. 2018 (17) | Older women aged ≥60 y; physically independent; free from cardiac or orthopaedic dysfunction; protein intake <1.2 g/kg BW; mean age: 68 ± 4 y; mean BMI: 26 ± 3 kg/m^2^; race NR | Whey protein. 35 g of hydrolysed whey protein containing 27.1 g of protein, dissolved in 200 ml sugar-free soft drink, ingested after WBR (so only on training days), [A]  WBR, 3 times/wk, 3 sets of 8-12RM | CHO placebo. 35 g of maltodextrin, dissolved in 200 ml sugar-free soft drink, ingested after WBR (so only on training days)  WBR, 3 times/wk, 3 sets of 8-12RM | NR | Baseline:  IG: 0.85 ± 0.1, CG: 0.81 ± 0.1 Follow-up:  IG: 1.4 ± 0.1, CG: 0.87 ± 0.1 | 12 wk | High | Total BMC (kg; DXA) | 16/16 | | Mean % change:  IG: +1.3  CG: +0.8  P for time*group interaction=0.76 | | |

Abbreviations: aBMD, areal BMD; BMC, bone mineral content; BMD, bone mineral density; BMI, body mass index; BW, body weight; CI, confidence interval; CG, control group; CHO, carbohydrates; CSA, cross-sectional area; CSMI, cross-sectional moment of inertia; CTX, C-terminal telopeptide of type 1 collagen; DXA, dual-energy X-ray absorptiometry; EAA, essential amino acids; IG, intervention group; ITT, intention-to-treat analysis or modified intention-to-treat analysis (mITT; only those with missing outcome data were excluded from the analytic sample); IU, international units; LBM, lean body mass; LSMD, least square mean difference; MD, mean difference (i.e. difference in within-group change); mg, milligram; mo, months; *n*, number; NR, not reported; NS, not significant; OC, osteocalcin; P1NP, N-terminal propeptides of type 1 procollagen; PP, per-protocol analysis; QCT, quantitative computed tomography; RM, repetition maximum; SD, standard deviation; SEM, standard error of the mean; vBMD, volumetric BMD; WBR, whole-body resistance training; wk, weeks; y, years.

Statistically significant effects are shown in bold.

^a^ ‘Protein type’ indicates the way in which a higher protein intake was achieved and is categorized into ‘pure’ protein or amino acids (or essential amino acids) (A), specific product with a high protein content (B) or high-protein diets (C).

^b^ Risk of bias was assessed using the RoB 2 Cochrane collaboration tool and scored as ‘low’ (L), ‘some concerns’ (SC) or ‘high’ (H).

^c^ Neck bone cross-sectional area (CSA) relates to strength in compression.

^d^ Buckling ratio relates to strength in buckling.

^e^ Polar cross-sectional moment of inertia (CSMI) relates to strength in torsion.

Supplemental Table 5

Results from randomized controlled trials on the effect of increased protein intake on blood pressure in older adults, grouped according to whether or not the protein intervention was carried out in the context of (concomitant) physical exercise

| Study, country | Study population | Exposure (IG), [protein type^a^] | Comparison (CG) | Compliance | Total protein intake (g/kg BW/d) | Study duration | Risk of bias^b^ | Outcome | Analytic *n* IG/CG | Results | | |
| --- | --- | --- | --- | --- | --- | --- | --- | --- | --- | --- | --- | --- |
| Not in the context of (concomitant) physical exercise | | | | | | | | | | | | |
| Hodgson et al. 2012 (10), Australia  Same RCT as Zhu et al. 2011 (11) and Zhu et al. 2015 (9) | Community-dwelling older women aged 70-80 y with habitual protein intake <1.5 g/kg BW/d; without metabolic bone disease, osteoporotic fracture, diabetes, hepatic or renal insufficiency; mean age: 74 ± 3 y; mean BMI: 27 ± 4 kg/m^2^; Caucasian | Whey protein isolate. 250 ml skim milk-based high-protein supplement drink containing 30 g of whey protein + calcium, [A] | CHO placebo. 250 ml skim milk-based supplement drink containing 2.1 g of protein and isocaloric amount of maltodextrin + calcium | Mean ± SD (1y, *n*=196) IG: 78 ± 29%, CG: 72 ± 31%  Mean ± SD (2y, *n*=181) IG: 88 ± 25%, CG: 81 ± 25% | Baseline:  IG: 1.2 ± 0.3, CG: 1.1 ± 0.3 Follow-up (2 y): IG: 1.4 ± 0.4, CG: 1.1 ± 0.4 | 1 y and 2 y | Some concerns | Systolic BP^§^ (mm Hg) | 1 y: 109/110 (mITT^c^) | MD (95%-CI):  -2.3 (-5.3 to +0.7)  P=0.14 | | |
|  |  |  |  |  |  |  |  |  | 2 y: 109/110 (mITT^c^) | MD (95%-CI):  +1.6 (-1.5 to +4.7)  P=0.30 | | |
|  |  |  |  |  |  |  |  | Diastolic BP^§^ (mm Hg) | 1 y: 109/110  (mITT^c^) | MD (95%-CI):  -1.5 (-3.6 to +0.6)  P=0.15 | | |
|  |  |  |  |  |  |  |  |  | 2 y: 109/110  (mITT^c^) | MD (95%-CI):  +0.3 (-1.9 to +2.4)  P=0.82 | | |
| Wright et al. 2018 (8), USA | Older adults aged 50-80 y with overweight or obesity (BMI of 25-38 kg/m^2^); without diabetes; 45% female; mean age: 70 ± 5 y; mean BMI: 31 ± 3 kg/m^2^; race NR | High-protein diet: 1.4 g/kg BW/d (~27 E% protein, ~43 E% CHO, ~30 E% fat). Majority of additional protein (59% = ~30 g = ~0.35 g/kg BW/d) came from eggs, [C] | Normal-protein diet: 0.8 g/kg BW/d (~15 E% protein, ~55 E% CHO, ~30 E% fat)    (Normal-protein diet provided on average ~50 g/d less protein than high-protein diet.) | 91% (overall) | Baseline:  IG: 84 ± 15 g/d, CG: 79 ± 15 g/d (calculated by using mean BW: IG: 0.93 g/kg BW/d, CG: 0.88 g/kg BW/d  Follow-up:  NR | 12 wk | High | Systolic BP (mm Hg) | 12/10  (PP) | | Mean change ± SD:  IG: -7 ± 13 CG: -2 ± 12  Time*group interaction NS |  |
|  |  |  |  |  |  |  |  | Diastolic BP (mm Hg) | 12/10  (PP) | | Mean change ± SD:  IG: -5 ± 5 CG: -2 ± 6  Time*group interaction NS |  |
| In the context of (concomitant) physical exercise | | | | | | | | | | | | |
| Nabuco et al. 2019a (20), Brazil  Same RCT as Nabuco et al. 2018 (19) and Nabuco et al. 2019b (21) | Older women aged ≥60 y, physically independent, free from cardiac or orthopaedic dysfunction; mean age: 67 ± 7 y; mean BMI: 25 ± 5 kg/m^2^; race NR | Whey protein. 35 g of hydrolysed whey protein supplement containing 27.1 g protein (+ 5.2 g CHO), mixed with non-caloric drink. IG1: protein before and placebo after WBR; IG2: placebo before and protein after WBR (so only on training days), [A]  WBR, 3 times/wk, 3 sets of 8-12 reps | CHO placebo containing 0.3 g protein and 33.3 g CHO, mixed with non-caloric drink; one before and one after WBR (so only on training days)  WBR, 3 times/wk, 3 sets of 8-12 reps | NR | Baseline:  IG1: 0.92 ± 0.20, IG2: 0.94 ± 0.36, CG: 0.95 ± 0.27  Follow-up:  IG1: 1.38 ± 0.26, IG2: 1.49 ± 0.46, CG: 1.0 ± 0.25 | 12 wk | Some concerns | Systolic BP (mm Hg) | 22/21/23  (mITT) | | Mean % change:  IG1: -2.1  IG2: -0.1  CG: -3.8  P for time*group interaction=0.304 |  |
|  |  |  |  |  |  |  |  | Diastolic BP (mm Hg) | 22/21/23  (mITT) | | Mean % change:  IG1: -1.1  IG2: +0.5  CG: -3.7  P for time*group interaction=0.178 |  |
| Nabuco et al. 2019c (22), Brazil | Older women aged ≥60 y with sarcopenic obesity (body fat mass ≥35% and ALST <15.02 kg); physically independent; free from cardiac or orthopaedic dysfunction; mean age: 69 ± 4 y; mean BMI: 27 ± 3 kg/m^2^; race NR | Whey protein. 35 g of hydrolysed whey protein supplement, mixed with non-caloric drink, ingested after WBR (so only on training days), [A]  WBR, 3 times/wk, 3 sets of 8-12 reps | CHO placebo. Isocaloric amount of maltodextrin mixed with non-caloric drink, ingested after WBR (so only on training days)  WBR, 3 times/wk, 3 sets of 8-12 reps | NR | Baseline  IG: 0.93 ± 0.36, CG: 0.97 ± 0.28  Follow-up:  IG: 1.0 ± 0.23, CG: 1.0 ± 0.19 (without supplementation) | 12 wk | Some concerns | Systolic BP (mm Hg) | 13/13  (ITT) | | Mean % change:  IG: -0.5  CG: +1.5  P for time*group interaction=0.451 |  |
|  |  |  |  |  |  |  |  | Diastolic BP (mm Hg) | 13/13  (ITT) | | Mean % change:  IG: -1.9  CG: -3.0  P for time*group interaction=0.702 |  |

Abbreviations: BMI, body mass index; BW, body weight; CI, confidence interval; CG, control group; CHO, carbohydrates; E%, percentage of energy intake; EAA, essential amino acids; IG, intervention group; ITT, intention-to-treat analysis or modified intention-to-treat analysis (mITT; only those with missing outcome data were excluded from the analytic sample); MD, mean difference (i.e. difference in within-group change); *n*, number; NR, not reported; NS, not significant; PP, per-protocol analysis; reps, repetitions; SD, standard deviation; WBR, whole-body resistance training; wk, weeks; y, years.

Statistically significant effects are shown in bold.

^a^ ‘Protein type’ indicates the way in which a higher protein intake was achieved and is categorized into ‘pure’ protein or amino acids (or essential amino acids) (A), specific product with a high protein content (B) or high-protein diets (C).

^b^ Risk of bias was assessed using the RoB 2 Cochrane collaboration tool and scored as ‘low’ (L), ‘some concerns’ (SC) or ‘high’ (H).

^c^ Per-protocol analysis demonstrated similar results to intention-to-treat analysis (data available).

Supplemental Table 6

Results from randomized controlled trials on the effect of increased protein intake on serum glucose and insulin in older adults, grouped according to whether or not the protein intervention was carried out in the context of (concomitant) physical exercise

| Study, country | Study population | Exposure (IG), [protein type^a^] | Comparison (CG) | Compliance | Total protein intake (g/kg BW/d) | Study duration | Risk of bias^b^ | Outcome | Analytic *n* IG/CG | Results | |  |  |
| --- | --- | --- | --- | --- | --- | --- | --- | --- | --- | --- | --- | --- | --- |
| Not in the context of (concomitant) physical exercise | | | | | | | | | | | | |  |
| Ottestad et al. 2017 (6), Norway | Community-dwelling older adults aged ≥70 y; relatively healthy (no diabetes, CVD, cancer, COPD, CKD); not malnourished; with reduced muscle strength or performance; 67% female; mean age: 77 ± 5 y; mean BMI: 27 ± 5 kg/m^2^; race NR | Protein-enriched milk. 400 ml drink containing 20 g protein (80:20 casein:whey mix, consumed twice a day (total: 40 g protein/d), [B] | CHO placebo. 400 ml drink containing an isocaloric amount of CHO, consumed twice a day | IG: 97.8 ± 3.8%, CG: 96.8 ± 5.7% | Baseline:  IG: 1.0 ± 0.3, CG: 1.0 ± 0.3  Follow-up:  IG: 1.4 ± 0.5, CG: 0.9 ± 0.4 | 12 wk | High | Fasting blood glucose (mmol/L) | 17/18  (mITT) | | Mean change (95%-CI):  IG: -0.1 (-0.3 to +0.2)  CG: +0.1 (-0.1 to +0.3)  P=0.36 | | |
| Park et al. 2018 (7), Korea | Community-dwelling (pre-)frail older adults aged 70-85 y at risk of malnutrition (MNA ≤23.5); no kidney or liver failure; able to walk; 65% female; mean age: 77 ± 4 y; mean BMI: 24 ± 3 kg/m^2^; Asian | Whey protein. Multiple 10-g packs of protein powder (9.3 g whey protein/pack), dissolved in 340 ml tea, were provided in addition to habitual protein intake up to 1.2 (IG1) or 1.5 (IG2) g/kg BW/d, [A] | CHO placebo. Multiple 10-g packs of CHO powder (9.3 g maltodextrin/ pack), dissolved in 340 ml tea, were provided in addition to habitual protein intake up to 0.8 (CG) or 1.2 (IG1) g/kg BW/d. (CG were given only CHO powder, IGs were given a combination of protein and CHO powder.) | IG1: 98%, IG2: 96%, CG: 97% | Baseline:  IG1: 0.77 ± 0.24, IG2: 0.80 ± 0.21, CG: 0.84 ± 0.28  Follow-up:  IG1: 1.18 ± 0.23, IG2: 1.37 ± 0.26, CG: 0.90 ± 0.38 | 12 wk | Some concerns | Fasting blood glucose (mmol/L) | 40/40/40  (ITT) | | P for time*group interaction=0.315 | | |
| Wright et al. 2018 (8), USA | Older adults aged 50-80 y with overweight or obesity (BMI of 25-38 kg/m^2^); without diabetes; 45% female; mean age: 70 ± 5 y; mean BMI: 31 ± 3 kg/m^2^; race NR | High-protein diet: 1.4 g/kg BW/d (~27 E% protein, ~43 E% CHO, ~30 E% fat). Majority of additional protein (59% = ~30 g = ~0.35 g/kg BW/d) came from eggs, [C] | Normal-protein diet: 0.8 g/kg BW/d (~15 E% protein, ~55 E% CHO, ~30 E% fat)    (Normal-protein diet provided on average ~50 g/d less protein than high-protein diet.) | 91% (overall) | Baseline:  IG: 84 ± 15 g/d, CG: 79 ± 15 g/d (calculated by using mean BW: IG: 0.93 g/kg BW/d, CG: 0.88 g/kg BW/d  Follow-up:  NR | 12 wk | High | Fasting blood glucose (mmol/L) | 12/10  (PP) | | Mean change ± SD:  IG: -0.1 ± 0.4 CG: 0.0 ± 0.6  Time*group interaction NS | | |
|  |  |  |  |  |  |  |  | Fasting insulin (mmol/L) | 12/10  (PP) | | Mean change ± SD:  IG: -35.4 ± 34.7 CG: -8.3 ± 38.9  Time*group interaction NS | | |
|  |  |  |  |  |  |  |  | HOMA-IR (score) | 12/10  (PP) | | Mean change ± SD:  IG: -1.37 ± 1.51 CG: -0.35 ± 1.59  Time*group interaction NS | | |
| In the context of (concomitant) physical exercise | | | | | | | | | | | | |  |
| Fernandes et al. 2018 (16), Brazil  Same RCT as Sugihara Junior et al. 2018 (17) | Older women aged ≥60 y; physically independent; free from cardiac or orthopaedic dysfunction; protein intake <1.2 g/kg BW; mean age: 68 ± 4 y; mean BMI: 26 ± 3 kg/m^2^; race NR | Whey protein. 35 g of hydrolysed whey protein containing 27.1 g of protein, dissolved in 200 ml sugar-free soft drink, ingested after WBR (so only on training days), [A]  WBR, 3 times/wk, 3 sets of 8-12RM | CHO placebo. 35 g of maltodextrin, dissolved in 200 ml sugar-free soft drink, ingested after WBR (so only on training days)  WBR, 3 times/wk, 3 sets of 8-12RM | NR | Baseline:  IG: 0.85 ± 0.1, CG: 0.81 ± 0.1 Follow-up:  IG: 1.4 ± 0.1, CG: 0.87 ± 0.1 | 12 wk | High | Fasting blood glucose (mg/dL) | 16/16 | | Mean % change:  IG: -3.3  CG: +4.0  P for time*group interaction=0.42 | | |
| Nabuco et al. 2019a (20), Brazil  Same RCT as Nabuco et al. 2018 (19) and Nabuco et al. 2019b (21) | Older women aged ≥60 y, physically independent, free from cardiac or orthopaedic dysfunction; mean age: 67 ± 7 y; mean BMI: 25 ± 5 kg/m^2^; race NR | Whey protein. 35 g of hydrolysed whey protein supplement containing 27.1 g protein (+ 5.2 g CHO), mixed with non-caloric drink. IG1: protein before and placebo after WBR; IG2: placebo before and protein after WBR (so only on training days), [A]  WBR, 3 times/wk, 3 sets of 8-12 reps | CHO placebo containing 0.3 g protein and 33.3 g CHO, mixed with non-caloric drink; one before and one after WBR (so only on training days)  WBR, 3 times/wk, 3 sets of 8-12 reps | NR | Baseline:  IG1: 0.92 ± 0.20, IG2: 0.94 ± 0.36, CG: 0.95 ± 0.27  Follow-up:  IG1: 1.38 ± 0.26, IG2: 1.49 ± 0.46, CG: 1.0 ± 0.25 | 12 wk | Some concerns | Fasting blood glucose (mg/dL) | 22/21/23  (mITT) | | Mean % change:  IG1: -5.0  IG2: -0.2  CG: -0.2  P for time*group interaction=0.319 | | |
|  |  |  |  |  |  |  |  | Fasting insulin (µU/mL) | 22/21/23  (mITT) | | Mean % change:  IG1: -4.1  IG2: +7.3  CG: +0.5  P for time*group interaction=0.125 | | |
|  |  |  |  |  |  |  |  | HOMA-IR (score) | 22/21/23  (mITT) | | Mean % change:  IG1: -11.6  IG2: -18.8  CG: -8.1  P for time*group interaction=0.372 | | |
| Nabuco et al. 2019c (22), Brazil | Older women aged ≥60 y with sarcopenic obesity (body fat mass ≥35% and ALST <15.02 kg); physically independent; free from cardiac or orthopaedic dysfunction; mean age: 69 ± 4 y; mean BMI: 27 ± 3 kg/m^2^; race NR | Whey protein. 35 g of hydrolysed whey protein supplement, mixed with non-caloric drink, ingested after WBR (so only on training days), [A]  WBR, 3 times/wk, 3 sets of 8-12 reps | CHO placebo. Isocaloric amount of maltodextrin mixed with non-caloric drink, ingested after WBR (so only on training days)  WBR, 3 times/wk, 3 sets of 8-12 reps | NR | Baseline  IG: 0.93 ± 0.36, CG: 0.97 ± 0.28  Follow-up:  IG: 1.0 ± 0.23, CG: 1.0 ± 0.19 (without supplementation) | 12 wk | Some concerns | Fasting blood glucose (mg/dL) | 13/13  (ITT) | | Mean % change:  IG: -4.1  CG: -1.0  P for time*group interaction=0.251 | | |
|  |  |  |  |  |  |  |  | Fasting insulin (µU/mL) | 13/13  (ITT) | | Mean % change:  IG: -4.9  CG: -1.9  P for time*group interaction=0.774 | | |
|  |  |  |  |  |  |  |  | HOMA-IR (score) | 13/13  (ITT) | | Mean % change:  IG: -7.5  CG: -0.2  P for time*group interaction=0.511 | | |

Abbreviations: BMI, body mass index; BW, body weight; CG, control group; CHO, carbohydrates; CKD, chronic kidney disease; COPD, chronic obstructive pulmonary disease; CVD, cardiovascular disease; E%, percentage of energy intake; EAA, essential amino acids; HOMA-IR, homeostatic model assessment of insulin resistance; IG, intervention group; ITT, intention-to-treat analysis or modified intention-to-treat analysis (mITT; only those with missing outcome data were excluded from the analytic sample); MNA, Mini Nutritional Assessment; *n*, number; NR, not reported; NS, not significant; PP, per-protocol analysis; reps, repetitions; RM, repetition maximum; SD, standard deviation; WBR, whole-body resistance training; wk, weeks.

Statistically significant effects are shown in bold.

^a^ ‘Protein type’ indicates the way in which a higher protein intake was achieved and is categorized into ‘pure’ protein or amino acids (or essential amino acids) (A), specific product with a high protein content (B) or high-protein diets (C).

^b^ Risk of bias was assessed using the RoB 2 Cochrane collaboration tool and scored as ‘low’ (L), ‘some concerns’ (SC) or ‘high’ (H).

Supplemental Table 7

Results from randomized controlled trials on the effect of increased protein intake on serum lipids in older adults, grouped according to whether or not the protein intervention was carried out in the context of (concomitant) physical exercise

| Study reference, country | Study population | Exposure (IG), [protein type^a^] | Comparison (CG) | Compliance | Total protein intake (g/kg BW/d) | Study duration | Risk of bias^b^ | Outcome | Analytic *n* IG/CG | Results | |
| --- | --- | --- | --- | --- | --- | --- | --- | --- | --- | --- | --- |
| Not in the context of (concomitant) physical exercise | | | | | | | | | | |  |
| Bhasin et al. 2018 (1), USA | Community-dwelling older men aged ≥65 y with moderate physical function limitations and with habitual protein intake ≤0.83 g/kg BW/d; mean age: 73 ± 6 y; mean BMI: 30.3 ± 4.9 kg/m^2^; race NR | Individualised diets providing 0.7 g protein/kg BW/d with additional discretionary foods (0.1 g protein/kg BW) and protein supplements (0.5 g/kg BW; 50:50 casein:whey mix) to achieve a total of 1.3 g/kg BW/d, [A,B] | Individualised diets providing 0.7 g protein/kg BW/d with additional discretionary foods (0.1 g protein/kg BW) and placebo supplements (0.5 g CHO/kg BW) to achieve a total of 0.8 g/kg BW/d | Foods (4-6 mo):  IG: 77.1 ± 13%, CG: 74.5 ± 23.2%  Supplements (4-6 mo):  IG: 91.2 ± 12.4%, CG: 92.6 ± 11.0% | Baseline:  IG: 0.72 ± 0.11, CG: 0.69 ± 0.15  Follow-up (1-3 mo):  IG: 1.18 ± 0.15, CG: 0.84 ± 0.07  Follow-up (4-6 mo): IG: 1.17 ± 0.13, CG: 0.81 ± 0.10 | 6 mo | Some concerns | Total cholesterol (mg/dL) | 40-46/  38-46^c^  (mITT) | MD (95%-CI):  -4.45 (-13.10 to +4.19)  P=0.308 | |
|  |  |  |  |  |  |  |  | LDL cholesterol (mg/dL) | 40-46/  38-46^c^  (mITT) | MD (95%-CI):  -2.71 (-10.03 to +4.61)  P=0.463 | |
|  |  |  |  |  |  |  |  | HDL cholesterol (mg/dL) | 40-46/  38-46^c^  (mITT) | MD (95%-CI):  +1.95 (-0.67 to +4.56)  P=0.142 | |
|  |  |  |  |  |  |  |  | Triglycerides (mg/dL) | 40-46/  38-46^c^  (mITT) | MD (95%-CI):  -19.62 (-39.71 to +0.47)  P=0.055 | |
| Ottestad et al. 2017 (6), Norway | Community-dwelling older adults aged ≥70 y; relatively healthy (no diabetes, CVD, cancer, COPD, CKD); not malnourished; with reduced muscle strength or performance; 67% female; mean age: 77 ± 5 y; mean BMI: 27 ± 5 kg/m^2^; race NR | Protein-enriched milk. 400 ml drink containing 20 g protein (80:20 casein:whey mix, consumed twice a day (total: 40 g protein/d), [B] | CHO placebo. 400 ml drink containing an isocaloric amount of CHO, consumed twice a day | IG: 97.8 ± 3.8%, CG: 96.8 ± 5.7% | Baseline:  IG: 1.0 ± 0.3, CG: 1.0 ± 0.3  Follow-up:  IG: 1.4 ± 0.5, CG: 0.9 ± 0.4 | 12 wk | High | Total cholesterol (mmol/L) | 16/18  (mITT) | Mean change (95%-CI):  IG: -0.5 (-0.8 to -0.2)  CG: -0.1 (-0.4 to +0.2)  P=0.06 | |
|  |  |  |  |  |  |  |  | LDL cholesterol (mmol/L) | 16/18  (mITT) | Mean change (95%-CI):  IG: -0.3 (-0.5 to -0.1)  CG: -0.1 (-0.3 to +0.1)  P=0.25 | |
|  |  |  |  |  |  |  |  | HDL cholesterol (mmol/L) | 17/18  (mITT) | Mean change (95%-CI):  IG: 0.0 (-0.1 to +0.1)  CG: 0.0 (-0.0 to +0.1)  P=0.41 | |
|  |  |  |  |  |  |  |  | Triglycerides (mmol/L) | 16/18  (mITT) | Mean change (95%-CI):  IG: -0.1 (-0.3 to 0.0)  CG: +0.1 (-0.2 to +0.2)  **P=0.05** | |
| Park et al. 2018 (7), Korea | Community-dwelling (pre-)frail older adults aged 70-85 y at risk of malnutrition (MNA ≤23.5); no kidney or liver failure; able to walk; 65% female; mean age: 77 ± 4 y; mean BMI: 24 ± 3 kg/m^2^; Asian | Whey protein. Multiple 10-g packs of protein powder (9.3 g whey protein/pack), dissolved in 340 ml tea, were provided in addition to habitual protein intake up to 1.2 (IG1) or 1.5 (IG2) g/kg BW/d, [A] | CHO placebo. Multiple 10-g packs of CHO powder (9.3 g maltodextrin per/pack), dissolved in 340 ml tea, were provided in addition to habitual protein intake up to 0.8 (CG) or 1.2 (IG1) g/kg BW/d  (CG were given only CHO powder, IGs were given a combination of protein and CHO powder.) | IG1: 98%, IG2: 96%, CG: 97% | Baseline:  IG1: 0.77 ± 0.24, IG2: 0.80 ± 0.21, CG: 0.84 ± 0.28  Follow-up:  IG1: 1.18 ± 0.23, IG2: 1.37 ± 0.26, CG: 0.90 ± 0.38 | 12 wk | Some concerns | Total cholesterol (mmol/L) | 40/40/40  (ITT) | P for time*group interaction=0.478 | |
|  |  |  |  |  |  |  |  | LDL cholesterol (mmol/L) | 40/40/40  (ITT) | P for time*group interaction=0.887 | |
|  |  |  |  |  |  |  |  | HDL cholesterol (mmol/L) | 40/40/40  (ITT) | P for time*group interaction=0.363 | |
|  |  |  |  |  |  |  |  | Triglycerides (mmol/L) | 40/40/40  (ITT) | P for time*group interaction=0.837 | |
| Wright et al. 2018 (8), USA | Older adults aged 50-80 y with overweight or obesity (BMI of 25-38 kg/m^2^); without diabetes; 45% female; mean age: 70 ± 5 y; mean BMI: 31 ± 3 kg/m^2^; race NR | High-protein diet: 1.4 g/kg BW/d (~27 E% protein, ~43 E% CHO, ~30 E% fat). Majority of additional protein (59% = ~30 g = ~0.35 g/kg BW/d) came from eggs, [C] | Normal-protein diet: 0.8 g/kg BW/d (~15 E% protein, ~55 E% CHO, ~30 E% fat)    (Normal-protein diet provided on average ~50 g/d less protein than high-protein diet.) | 91% (overall) | Baseline:  IG: 84 ± 15 g/d, CG: 79 ± 15 g/d (calculated by using mean BW: IG: 0.93 g/kg BW/d, CG: 0.88 g/kg BW/d  Follow-up:  NR | 12 wk | High | Total cholesterol (mmol/L) | 12/10  (PP) | Mean change ± SD:  IG: -0.1 ± 0.6 CG: -0.2 ± 0.4  Time*group interaction NS | |
|  |  |  |  |  |  |  |  | LDL cholesterol (mmol/L) | 12/10  (PP) | Mean change ± SD:  IG: +0.1 ± 0.4 CG: -0.3 ± 0.3  **Time*group interaction<0.05** | |
|  |  |  |  |  |  |  |  | HDL cholesterol (mmol/L) | 12/10  (PP) | Mean change ± SD:  IG: -0.1 ± 0.2 CG: -0.1 ± 0.2  Time*group interaction NS | |
|  |  |  |  |  |  |  |  | Triglycerides (mmol/L) | 12/10  (PP) | Mean change ± SD:  IG: -0.3 ± 0.6 CG: +0.1 ± 0.4  Time*group interaction NS | |
|  |  |  |  |  |  |  |  | Total/HDL cholesterol ratio | 12/10  (PP) | Mean change ± SD:  IG: +0.14 ± 0.36 CG: -0.21 ± 0.56  Time*group interaction NS | |
| In the context of (concomitant) physical exercise | | | | | | | | | | |  |
| Fernandes et al. 2018 (16), Brazil  Same RCT as Sugihara Junior et al. 2018 (17) | Older women aged ≥60 y; physically independent; free from cardiac or orthopaedic dysfunction; protein intake <1.2 g/kg BW; mean age: 68 ± 4 y; mean BMI: 26 ± 3 kg/m^2^; race NR | Whey protein. 35 g of hydrolysed whey protein containing 27.1 g of protein, dissolved in 200 ml sugar-free soft drink, ingested after WBR (so only on training days), [A]  WBR, 3 times/wk, 3 sets of 8-12RM | CHO placebo. 35 g of maltodextrin, dissolved in 200 ml sugar-free soft drink, ingested after WBR (so only on training days)  WBR, 3 times/wk, 3 sets of 8-12RM | NR | Baseline:  IG: 0.85 ± 0.1, CG: 0.81 ± 0.1 Follow-up:  IG: 1.4 ± 0.1, CG: 0.87 ± 0.1 | 12 wk | High | Total cholesterol (mg/dL) | 16/16 | Mean % change:  IG: -2.8  CG: +0.5  P for time*group interaction=0.33 | |
|  |  |  |  |  |  |  |  | LDL cholesterol (mg/dL)^c^ | 16/16 | Mean % change:  IG: -6.8  CG: +0.9  P for time*group interaction=0.14 | |
|  |  |  |  |  |  |  |  | HDL cholesterol (mg/dL) | 16/16 | Mean % change:  IG: +6.7  CG: +6.3  P for time*group interaction=0.78 | |
|  |  |  |  |  |  |  |  | Triglycerides (mg/dL) | 16/16 | Mean % change:  IG: -2.0  CG: -1.2  P for time*group interaction=0.93 | |
|  |  |  |  |  |  |  |  | Total/HDL cholesterol ratio | 16/16 | Mean % change:  IG: -11.8  CG: -7.3  **P for time*group interaction=0.04** | |
|  |  |  |  |  |  |  |  | LDL/HDL cholesterol ratio^c^ | 16/16 | Mean % change:  IG: -11.5  CG: -6.9  P for time*group interaction=0.42 | |
| Nabuco et al. 2019a (20), Brazil  Same RCT as Nabuco et al. 2018 (19) and Nabuco et al. 2019b (21) | Older women aged ≥60 y, physically independent, free from cardiac or orthopaedic dysfunction; mean age: 67 ± 7 y; mean BMI: 25 ± 5 kg/m^2^; race NR | Whey protein. 35 g of hydrolysed whey protein supplement containing 27.1 g protein (+ 5.2 g CHO), mixed with non-caloric drink. IG1: protein before and placebo after WBR; IG2: placebo before and protein after WBR (so only on training days), [A]  WBR, 3 times/wk, 3 sets of 8-12 reps | CHO placebo containing 0.3 g protein and 33.3 g CHO, mixed with non-caloric drink; one before and one after WBR (so only on training days)  WBR, 3 times/wk, 3 sets of 8-12 reps | NR | Baseline:  IG1: 0.92 ± 0.20, IG2: 0.94 ± 0.36, CG: 0.95 ± 0.27  Follow-up:  IG1: 1.38 ± 0.26, IG2: 1.49 ± 0.46, CG: 1.0 ± 0.25 | 12 wk | Some concerns | Total cholesterol (mg/dL) | 22/21/23  (mITT) | Mean % change:  IG1: +4.9  IG2: +4.3  CG: +2.0  P for time*group interaction=0.357 | |
|  |  |  |  |  |  |  |  | LDL cholesterol (mg/dL)^d^ | 22/21/23  (mITT) | Mean % change:  IG1: -3.3  IG2: +1.0  CG: +0.3  P for time*group interaction=0.683 | |
|  |  |  |  |  |  |  |  | HDL cholesterol (mg/dL) | 22/21/23  (mITT) | Mean % change:  IG1: -0.5  IG2: +3.3  CG: +3.7  P for time*group interaction=0.129 | |
|  |  |  |  |  |  |  |  | Triglycerides (mg/dL) | 22/21/23  (mITT) | Mean % change:  IG1: +0.9  IG2: -3.5  CG: -6.3  P for time*group interaction=0.348 | |
|  |  |  |  |  |  |  |  | Total/HDL cholesterol ratio | 22/21/23  (mITT) | Mean % change:  IG1: +5.2  IG2: 0  CG: -3.7  P for time*group interaction=0.081 | |
|  |  |  |  |  |  |  |  | LDL/HDL cholesterol ratio^d^ | 22/21/23  (mITT) | Mean % change:  IG1: -1.9  IG2: -4.5  CG: -2.9  P for time*group interaction=0.925 | |
| Nabuco et al. 2019c (22), Brazil | Older women aged ≥60 y with sarcopenic obesity (body fat mass ≥35% and ALST <15.02 kg); physically independent; free from cardiac or orthopaedic dysfunction; mean age: 69 ± 4 y; mean BMI: 27 ± 3 kg/m^2^; race NR | Whey protein. 35 g of hydrolysed whey protein supplement, mixed with non-caloric drink, ingested after WBR (so only on training days), [A]  WBR, 3 times/wk, 3 sets of 8-12 reps | CHO placebo. Isocaloric amount of maltodextrin mixed with non-caloric drink, ingested after WBR (so only on training days)  WBR, 3 times/wk, 3 sets of 8-12 reps | NR | Baseline  IG: 0.93 ± 0.36, CG: 0.97 ± 0.28  Follow-up:  IG: 1.0 ± 0.23, CG: 1.0 ± 0.19 (without supplementation) | 12 wk | Some concerns | Total cholesterol (mg/dL) | 13/13  (ITT) | Mean % change:  IG: -4.7  CG: -3.8  P for time*group interaction=0.847 | |
|  |  |  |  |  |  |  |  | LDL cholesterol (mg/dL)^d^ | 13/13  (ITT) | Mean % change:  IG: -7.8  CG: -3.0  P for time*group interaction=0.542 | |
|  |  |  |  |  |  |  |  | HDL cholesterol (mg/dL) | 13/13  (ITT) | Mean % change:  IG: +6.9  CG: +5.1  P for time*group interaction=0.689 | |
|  |  |  |  |  |  |  |  | Triglycerides (mg/dL) | 13/13  (ITT) | Mean % change:  IG: -12.0  CG: -8.7  P for time*group interaction=0.782 | |

Abbreviations: BMI, body mass index; BW, body weight; CI, confidence interval; CG, control group; CHO, carbohydrates; CKD, chronic kidney disease; COPD, chronic obstructive pulmonary disease; CVD, cardiovascular disease; E%, percentage of energy intake; EAA, essential amino acids; HDL, high-density lipoprotein; IG, intervention group; ITT, intention-to-treat analysis or modified intention-to-treat analysis (mITT; only those with missing outcome data were excluded from the analytic sample); LDL, low-density lipoprotein; MD, mean difference (i.e. difference in within-group change); MNA, Mini Nutritional Assessment; mo, months; *n*, number; NR, not reported; NS, not significant; PEDro, Physiotherapy Evidence Database scale; PP, per-protocol analysis; reps, repetitions; RM, repetition maximum; s, seconds; SD, standard deviation; SEM, standard error of the mean; SPPB, short physical performance battery; WBR, whole-body resistance training; wk, weeks.

Statistically significant effects are shown in bold.

^a^ ‘Protein type’ indicates the way in which a higher protein intake was achieved and is categorized into ‘pure’ protein or amino acids (or essential amino acids) (A), specific product with a high protein content (B) or high-protein diets (C).

^b^ Risk of bias was assessed using the RoB 2 Cochrane collaboration tool and scored as ‘low’ (L), ‘some concerns’ (SC) or ‘high’ (H).

^c^ The exact number of participants included in the analyses is not reported. The number must be between the number of participants who were randomized and the number of participants who completed the study.

^d^ LDL cholesterol was determined using the Friedewald equation: LDL cholesterol = total cholesterol - (HDL cholesterol + triglycerides / 5).

Supplemental Table 8

Results from randomized controlled trials on the effect of increased protein intake on kidney function in older adults, grouped according to whether or not the protein intervention was carried out in the context of (concomitant) physical exercise

| Study, country | Study population | Exposure (IG), [protein type^a^] | Comparison (CG) | Compliance | Total protein intake (g/kg BW/d) | Study duration | Risk of bias^b^ | Outcome | Analytic *n* IG/CG | Results |
| --- | --- | --- | --- | --- | --- | --- | --- | --- | --- | --- |
| Not in the context of (concomitant) physical exercise | | | | | | | | | | |
| Bhasin et al. 2018 (1), USA | Community-dwelling older men aged ≥65 y with moderate physical function limitations and with habitual protein intake ≤0.83 g/kg BW/d; mean age: 73 ± 6 y; mean BMI: 30.3 ± 4.9 kg/m^2^; race NR | Individualised diets providing 0.7 g protein/kg BW/d with additional discretionary foods (0.1 g protein/kg BW) and protein supplements (0.5 g/kg BW; 50:50 casein:whey mix) to achieve a total of 1.3 g/kg BW/d, [A,B] | Individualised diets providing 0.7 g protein/kg BW/d with additional discretionary foods (0.1 g protein/kg BW) and placebo supplements (0.5 g CHO/kg BW) to achieve a total of 0.8 g/kg BW/d | Foods (4-6 mo):  IG: 77.1 ± 13%, CG: 74.5 ± 23.2%  Supplements (4-6 mo):  IG: 91.2 ± 12.4%, CG: 92.6 ± 11.0% | Baseline:  IG: 0.72 ± 0.11, CG: 0.69 ± 0.15  Follow-up (1-3 mo):  IG: 1.18 ± 0.15, CG: 0.84 ± 0.07  Follow-up (4-6 mo): IG: 1.17 ± 0.13, CG: 0.81 ± 0.10 | 6 mo | Some concerns | Serum creatinine (mg/dL) | 40-46/  38-46^c^  (mITT) | MD (95%-CI):  -0.01 (-0.05 to +0.03)  P=0.540 |
| Kerstetter et al. 2015 (4), USA | Older men (aged >70 y) and women (aged >60 y) with BMI of 19-32 kg/m^2^ and protein intake of 0.6-1.0 g/kg BW; without major chronic diseases (e.g. diabetes, renal disease, inflammatory bowel disease) or cancer within past 18 months; 86% female; mean age: 70 ± 6 y; mean BMI: 26 ± 4 kg/m^2^; Caucasian | Whey protein. 45 g of whey protein isolate (~40 g of protein) + vitamin D (400 IU) + calcium (1200 mg), [A] | CHO placebo.  Isocaloric amount of maltodextrin + vitamin D (400 IU) + calcium (1200 mg) | NR | Baseline:  IG: 1.07 ± 0.03, CG: 1.06 ± 0.03  Follow-up:  IG: 1.30 ± 0.05, CG: 1.05 ± 0.04 | 9 mo and 18 mo | Some concerns | eGFR (mL/ min/1.73 m^2^) | 9 mo: 61/60  (mITT) | **P=0.006**  (increase in IG compared to CG) |
|  |  |  |  |  |  |  |  |  | 18 mo: 61/60  (mITT) | P=0.3394 |
| Ottestad et al. 2017 (6), Norway | Community-dwelling older adults aged ≥70 y; relatively healthy (no diabetes, CVD, cancer, COPD, CKD); not malnourished; with reduced muscle strength or performance; 67% female; mean age: 77 ± 5 y; mean BMI: 27 ± 5 kg/m^2^; race NR | Protein-enriched milk. 400 ml drink containing 20 g protein (80:20 casein:whey mix, consumed twice a day (total: 40 g protein/d), [B] | CHO placebo. 400 ml drink containing an isocaloric amount of CHO, consumed twice a day | IG: 97.8 ± 3.8%, CG: 96.8 ± 5.7% | Baseline:  IG: 1.0 ± 0.3, CG: 1.0 ± 0.3  Follow-up:  IG: 1.4 ± 0.5, CG: 0.9 ± 0.4 | 12 wk | High | Serum creatinine (µmol//L) | 17/18  (mITT) | Mean change (95%-CI):  IG: -0.1 (-2.8 to +2.7)  CG: +6.0 (+1.0 to +11.0)  **P=0.04** |
|  |  |  |  |  |  |  |  | eGFR (mL/ min/1.73 m^2^) | 17/18  (mITT) | Mean change (95%-CI):  IG: +0.29 (-3.1 to +2.5)  CG: -4.4 (-8.6 to -0.3)  P=0.09 |
| Park et al. 2018 (7), Korea | Community-dwelling (pre-)frail older adults aged 70-85 y at risk of malnutrition (MNA ≤23.5); no kidney or liver failure; able to walk; 65% female; mean age: 77 ± 4 y; mean BMI: 24 ± 3 kg/m^2^; Asian | Whey protein. Multiple 10-g packs of protein powder (9.3 g whey protein/pack), dissolved in 340 ml tea, were provided in addition to habitual protein intake up to 1.2 (IG1) or 1.5 (IG2) g/kg BW/d, [A] | CHO placebo. Multiple 10-g packs of CHO powder (9.3 g maltodextrin per/pack), dissolved in 340 ml tea, were provided in addition to habitual protein intake up to 0.8 (CG) or 1.2 (IG1) g/kg BW/d  (CG were given only CHO powder, IGs were given a combination of protein and CHO powder.) | IG1: 98%, IG2: 96%, CG: 97% | Baseline:  IG1: 0.77 ± 0.24, IG2: 0.80 ± 0.21, CG: 0.84 ± 0.28  Follow-up:  IG1: 1.18 ± 0.23, IG2: 1.37 ± 0.26, CG: 0.90 ± 0.38 | 12 wk | Some concerns | Serum creatinine (µmol//L) | 40/40/40  (ITT) | P for time*group interaction=0.265 |
|  |  |  |  |  |  |  |  | eGFR (mL/ min/1.73 m^2^ | 40/40/40  (ITT) | P for time*group interaction=0.277 |
| In the context of (concomitant) physical exercise | | | | | | | | | | |
| Ramel et al. 2013 (13), Iceland  Same RCT as Arnarson et al. 2013 (12) | Community-dwelling older men and women aged ≥65 y;  without major orthopaedic disease or musculoskeletal disorders; 58% female; mean age: 74 ± 6 y (range: 65-91 y); mean BMI: 29 ± 5 kg/m^2^ (82% ≥25 kg/m^2^); race NR | Whey or milk protein. Drink containing 20 g of whey protein isolate (+ 20 g of CHO; IG1); drink containing 20 g of milk protein isolate (+ 20 g of CHO; IG2), consumed after WBR (so on training days only), [A]  WBR, 3 times/wk, 3 sets of 6-8 reps, 75-80% 1RM | CHO placebo. Drink containing 40 g of CHO, consumed after WBR (so on training days only)  WBR, 3 times/wk, 3 sets of 6-8 reps, 75-80% 1RM | NR | Baseline:  IG1: 1.00 ± 0.26, IG2: NR; CG: 0.92 ± 0.30  Follow-up:  IG1: 1.06 ± 0.23, IG2: NR, CG 0.89 ± 0.23 | 12 wk | High | eGFR (mL/ min/1.73 m^2^) | 237 (total) | β (95%-CI) for IG1 vs. IG2:  -0.948 (-6.121 to +4.224) P=0.718  β (95%-CI) for CG vs. IG2:  -1.770 (-6.772 to +3.233) P=0.486 |
| Ten Haaf et al. 2019 (23), The Netherlands | Physically active older adults aged ≥65 y with habitual protein intake <1.0 g/kg BW; without type 2 diabetes, cancer, renal insufficiency (eGFR <30) or COPD; median age: 69 (range 67-73) y; mean BMI: 27 ± 3 kg/m^2^; Caucasian (except for 1 Asian participant) | Milk-protein concentrate. 250-ml protein drink containing 15.5 g protein (+ 1.1 g fat + 14.5 g lactose) consumed twice a day (total: 31 g protein/d), [A]  Training (walking) for the Nijmegen Four Days Marches | CHO placebo. 250-ml isocaloric drink containing 1.1 g protein (+ 5.2 g fat + 36 g CHO) consumed twice daily  Training (walking) for the Nijmegen Four Days Marches | IG: 96 ± 3%, CG: 95 ± 3% | Baseline:  IG: 0.86 ± 0.23, CG: 0.92 ± 0.24  Follow-up:  IG: 0.92 ± 0.27, CG: 0.97 ± 0.23 (without supplementation) | 12 wk | Some concerns | Serum creatinine (µmol//L) | 114  (total, PP) | Mean change ± SD:  IG: +3.9 ± 11.0  CG: +4.9 ± 6.8  P for time*group interaction=0.56 |
|  |  |  |  |  |  |  |  | eGFR (mL/ min/1.73 m^2^) | 109  (total, PP) | Mean change ± SD:  IG: -1.9 ± 9.8  CG: -4.1 ± 7.0  P for time*group interaction=0.19 |
|  |  |  |  |  |  |  |  | Albumin/creatinine ratio (mg/mmol; urine) | 111  (total, PP) | Mean change ± SD:  IG: -0.5 ± 3.3  CG: -0.4 ± 4.1  P for time*group interaction=0.86 |

Abbreviations: BMI, body mass index; BW, body weight; CI, confidence interval; CG, control group; CHO, carbohydrates; CKD, chronic kidney disease; COPD, chronic obstructive pulmonary disease; CVD, cardiovascular disease; EAA, essential amino acids; eGFR, estimated glomerular filtration rate; IG, intervention group; ITT, intention-to-treat analysis or modified intention-to-treat analysis (mITT; only those with missing outcome data were excluded from the analytic sample); IU, international units; MD, mean difference (i.e. difference in within-group change); mg, milligram; MNA, Mini Nutritional Assessment; mo, months; *n*, number; NR, not reported; NS, not significant; PP, per-protocol analysis; reps, repetitions; RM, repetition maximum; wk, weeks.

Statistically significant effects are shown in bold.

^a^ ‘Protein type’ indicates the way in which a higher protein intake was achieved and is categorized into ‘pure’ protein or amino acids (or essential amino acids) (A), specific product with a high protein content (B) or high-protein diets (C).

^b^ Risk of bias was assessed using the RoB 2 Cochrane collaboration tool and scored as ‘low’ (L), ‘some concerns’ (SC) or ‘high’ (H).

^c^ The exact number of participants included in the analyses is not reported. The number must be between the number of participants who were randomized and the number of participants who completed the study.

Supplemental Table 9

Results from randomized controlled trials on the effect of increased protein intake on cognition in older adults, grouped according to whether or not the protein intervention was carried out in the context of (concomitant) physical exercise

| Study, country | Study population | Exposure (IG), [protein type^a^] | Comparison (CG) | Compliance | Total protein intake (g/kg BW/d) | Study duration | Risk of bias^b^ | Outcome | Analytic *n* IG/CG | Results | |  |  |
| --- | --- | --- | --- | --- | --- | --- | --- | --- | --- | --- | --- | --- | --- |
| Not in the context of (concomitant) physical exercise | | | | | | | | | | | | |  |
| Park et al. 2018 (7), Korea | Community-dwelling (pre-)frail older adults aged 70-85 y at risk of malnutrition (MNA ≤23.5); no kidney or liver failure; able to walk; 65% female; mean age: 77 ± 4 y; mean BMI: 24 ± 3 kg/m^2^; Asian | Whey protein. Multiple 10-g packs of protein powder (9.3 g whey protein/pack), dissolved in 340 ml tea, were provided in addition to habitual protein intake up to 1.2 (IG1) or 1.5 (IG2) g/kg BW/d, [A] | CHO placebo. Multiple 10-g packs of CHO powder (9.3 g maltodextrin/ pack), dissolved in 340 ml tea, were provided in addition to habitual protein intake up to 0.8 (CG) or 1.2 (IG1) g/kg BW/d. (CG were given only CHO powder, IGs were given a combination of protein and CHO powder.) | IG1: 98%, IG2: 96%, CG: 97% | Baseline:  IG1: 0.77 ± 0.24, IG2: 0.80 ± 0.21, CG: 0.84 ± 0.28  Follow-up:  IG1: 1.18 ± 0.23, IG2: 1.37 ± 0.26, CG: 0.90 ± 0.38 | 12 wk | Some concerns | MMSE (score) | 40/40/40  (ITT) | | P for time*group interaction=0.702 | | |

Abbreviations: BW, body weight; CG, control group; CHO, carbohydrates; EAA, essential amino acids IG, intervention group; ITT, intention-to-treat analysis; MMSE, Mini-Mental State Examination; MNA, Mini Nutritional Assessment; *n*, number; NR, not reported; NS, not significant; wk, weeks.

Statistically significant effects are shown in bold.

^a^ ‘Protein type’ indicates the way in which a higher protein intake was achieved and is categorized into ‘pure’ protein or amino acids (or essential amino acids) (A), specific product with a high protein content (B) or high-protein diets (C).

^b^ Risk of bias was assessed using the RoB 2 Cochrane collaboration tool and scored as ‘low’ (L), ‘some concerns’ (SC) or ‘high’ (H).

Supplemental Table 10

Risk of bias assessment of included studies

| Study | Risk of bias domain^a^ | | | | | Overall RoB^b^ | Comments |  | Funding and author’s conflicts of interest^c^ |
| --- | --- | --- | --- | --- | --- | --- | --- | --- | --- |
|  | 1 | 2^d^ | 3 | 4 | 5 |  |  |  |  |
| Arnarson et al. 2013 (12) | + | + | x | + | + | x | Considerable proportion of missing outcome data (12%) and missingness in the outcome may depend on its true value. |  | Funding provided by: Icelandic Technology Development Fund, Research Fund of the University of Iceland, Landspitali University Hospital Research Fund, and Helga Jonsdottir and Sigurlidi Kristjansson Geriatric Research Fund. Authors declared no conflict of interest. |
| Bhasin et al. 2018 (1) | + | + | - | + | + | - | Missingness in the outcome may depend on its true value. |  | Funding provided by: NIA (NIH grant), National Center for Advancing Translational Sciences (Boston University Clinical Translational Science Institute grant; NIH Award), Boston Nutrition Obesity Research Center, and Harvard University and affiliated health care centres. Dietary supplements provided by: Abbott Laboratories, Bariatrix Nutrition and the National Dairy Council. Testosterone provided by: Endo Pharmaceuticals. Funding sources had no role in study design, study conduct, manuscript preparation and publication. Some authors declared conflicts of interest related to food companies (e.g. receiving grants from Abbott Pharmaceuticals, acting as a consultant for Novartis or AbbVie, receiving fees from Novo Nordisk, receiving research support from The Beef Checkoff Program). |
| Campbell et al. 1995 (1) | - | + | + | + | + | - | Unclear if allocation sequence was concealed. |  | Funding provided by: the US Department of Agricultural Research Service and NIH grant. Personal support by Kraft General Foods Predoctoral Fellowship (American Institute of Nutrition). Other authors’ conflicts of interest NR. |
| Chalé et al. 2013 (15) | + | - | + | - | + | - | Achieved protein intake is not reported for the entire analytic sample while ITT analyses were performed. For some outcomes, results of completer analyses and ITT analyses differed. Furthermore, it is unclear who assessed the outcomes and if the outcome assessors were blinded. It is unlikely that they were unblinded, but if they were, the outcome (e.g. muscle strength, performance) might have been influenced by the assessor’s knowledge of the intervention received. |  | Funding provided by: Boston Claude D. Pepper Older Americans Independence Center (NIH/NIA), the Boston Nutrition/Obesity Research Center, a postdoctoral training grant and the US Department of Agriculture. Personal funding by Dairy Research Institute. Other authors’ conflicts of interest NR. |
| Dillon et al. 2009 (2) | - | x | x | + | x | x | Unclear if allocation sequence was concealed. Also, no information was provided on the number of participants that were recruited, how many were randomized and how many were lost to follow-up (and thus unclear if outcome data were missing and/or if the correct analyses were performed). Moreover, only the time effect was reported and not the time*group interaction (while ANOVA was applied). |  | Funding provided by: Boston Claude D. Pepper Older Americans Independence Center (NIH/NIA), United States Public Health Service and General Clinical Research Center. Authors declared no conflict of interest. |
| Fernandes et al. 2018 (16) | - | - | x | + | x | x | No information was provided on allocation sequence. Furthermore, it is unclear if participants were lost to follow-up, and for what reason, and it is unclear what type of analysis (PP or (m)ITT or other) was applied. Also, possibility of selective reporting. |  | Funding provided by: Coordination for the Improvement of Higher Education Personnel, the Brazilian Ministry of Education and the Brazilian National Council for Scientific and Technological Development. Dietary supplements provided by: Arla Foods Ingredients and New Millen. Authors declared no conflict of interest. |
| Hodgson et al. 2012 (10) | + | + | - | + | + | - | Considerable proportion of missing outcome data (11%), but no strong evidence provided to show that the result was not biased by missing outcome data. |  | Funding provided by: Australian National Health Medical Research Council and University of Western Australia Research Grants Scheme. Dietary supplements provided by: Fonterra Brands Limited. Funding sources had no role in study design, study execution and manuscript preparation. Authors declared no conflict of interest. |
| Ispoglou et al. 2016 (3) | - | - | x | - | + | x | Loss to follow-up (for reasons including dietary non-compliance) was 18%, but no strong evidence provided to show that results were not biased by missing outcome data (and it is unclear whether different groups had the same drop-out rate and the same reason for drop-out); unclear if allocation sequence was concealed; unclear who assessed the outcomes and if the outcome assessors were blinded. If unblinded, some outcomes (e.g. muscle strength, performance) might have been influenced by the assessor’s knowledge of the intervention received. |  | Funding provided by: institutional grant. Authors declared no conflict of interest. |
| Kerstetter et a. 2015 (4) | - | + | - | + | + | - | Unclear if allocation sequence was concealed. No strong evidence provided to show that result was not biased by missing outcome data. |  | Funding provided by: NIH National Institute of Arthritis and Musculoskeletal and Skin Diseases, Dairy Research Institute and the Yale Bone Center. Authors declared no conflict of interest. |
| Mitchell et al. 2015 (18) | - | - | x | - | + | x | This was a brief communication. As a result, reporting was very limited and therefore risk of bias was difficult to judge. No information was provided on allocation sequence and baseline characteristics were not reported. Also, unclear how many participants were randomized, how many withdrew and how many were analysed, and information was lacking on the reasons for any drop-outs and/or missing outcome data. Furthermore, unclear who assessed the outcomes and if the outcome assessors were blinded^c^. If unblinded, the outcome (e.g. muscle strength) might have been influenced by knowledge of the intervention received. |  | Funding provided by: Dairy Farmers of Canada. Authors’ conflict of interest NR. |
| Mitchell et al. 2017 (5) | x | + | + | - | + | x | Allocation sequence probably was not concealed. Furthermore, assessment of the outcome (muscle strength, physical performance) might have been influenced by the outcome, since outcome assessors were probably not blinded^c^. |  | Funding provided by: New Zealand Ministry of Business, Innovation and Employment International Relationships and the European Union, and AgResearch Limited. Two authors are employees of AgResearch Limited. Other authors declared no conflict of interest. |
| Nabuco et al. 2018 (19) | - | + | + | - | + | - | Unclear if allocation sequence was concealed. Also, unclear who assessed the outcomes and if the outcome assessors were blinded^c^. It is unlikely that they were unblinded, but if they were, the outcome (e.g. muscle strength, performance) might have been influenced by knowledge of the intervention received. |  | Funding provided by: Coordination for the Improvement of Higher Education Personnel, National Council for Scientific and Technological Development, and Ministry of Education. Dietary supplements provided by: Arla Foods Ingredients Group and New Milen. Authors declared no conflict of interest. |
| Nabuco et al. 2019a (20) | - | + | + | + | - | - | In a previous paper from the same RCT (Nabuco 2018 (19)) other lean body mass parameters were addressed (upper limb LST, lower limb LST, skeletal muscle mass). In two of these a statistically significant effect was observed. It is unclear why appendicular LST was part of this separate paper (probably because the authors expected a significant effect on this outcome as well). |  | Funding provided by: Coordination for the Improvement of Higher Education Personnel, National Council for Scientific and Technological Development, and Ministry of Education. Dietary supplements provided by: Arla Foods Ingredients Group and New Milen. Authors declared no conflict of interest. |
| Nabuco et al. 2019b (21) | - | + | + | + | - | - | Unclear if allocation sequence was concealed. Furthermore, in two other papers (Nabuco 2018 (19); Nabuco 2019a (20)) other lean body mass parameters were addressed (upper limb LST, lower limb LST and skeletal muscle mass, and appendicular LST), for two of which a statistically significant effect was observed. It seems as if the authors expected a significant effect on total LST (as, indeed, there was) and, therefore, included the outcome in this next paper. |  | Funding provided by: Coordination for the Improvement of Higher Education Personnel, National Council for Scientific and Technological Development, and Ministry of Education. Dietary supplements provided by: Arla Foods Ingredients Group and New Milen. Authors declared no conflict of interest. |
| Nabuco et al. 2019c (22) | - | + | + | - | + | - | Unclear if allocation sequence was concealed. Also, unclear who assessed the outcomes and if the outcome assessors were blinded^c^. It is unlikely that they were unblinded but, if they were, the outcome (e.g. muscle strength, performance) might have been influenced by assessor’s knowledge of the intervention received. |  | Funding provided by: Coordination for the Improvement of Higher Education Personnel, National Council for Scientific and Technological Development, and Ministry of Education. Dietary supplements provided by: Arla Foods Ingredients Group and New Milen. Authors declared no conflict of interest. |
| Ottestad et al. 2017 (6) | + | + | x | + | + | x | The attrition rate was high (20%) and the missing outcome data might depend on its true value. |  | Funding provided by: Research Council of Norway. Dietary supplements provided by: TINE SA. Some authors declared conflicts of interest related to food or pharmacy companies (e.g. receiving grants or personal fees from Amgen, Mills DA, TINE DA or Olympic Seafood); none of which are related to the contents of this study. Other authors declared no conflict of interest. |
| Park et al. 2018 (7) | + | + | - | - | + | - | No strong evidence provided to show that results were not biased by missing outcome data (18%). Also, unclear if bias might feature in the outcome measurement (because it is not known who performed the outcome measurements). |  | Funding provided by: Korea Health Industry Development Institute (funded by the Ministry of Health & Welfare). Authors declared no conflict of interest. |
| Ramel et al. 2013 (13) | + | + | x | + | + | x | Missingness in the outcome may depend on the true value. |  | Funding provided by: Icelandic Technology Development Fund, Research Fund of the University of Iceland, Landspitali University Hospital Research Fund, and Helga Jonsdottir and Sigurlidi Kristjansson Geriatric Research Fund. Authors’ conflict of interest NR. |
| Sugihara Junior et al. 2018 (17) | - | - | x | + | + | x | No information was provided on allocation sequence. Also, unclear if participants were lost to follow-up (or that outcome data were missing; missing data could depend on its true value) and for what reasons; unclear what type of analysis (PP or (m)ITT or other) was applied. |  | Funding provided by: Coordination for the Improvement of Higher Education Personnel, the Brazilian Ministry of Education and the Brazilian National Council for Scientific and Technological Development. Dietary supplements provided by: Arla Foods Ingredients, New Millen and the Planeta Saúde Arapongas (supermarket). Authors declared no conflict of interest. |
| Ten Haaf et al. 2019 (23) | + | + | + | - | + | - | Unclear who performed the outcome assessments and if the outcome assessors were blinded. If unblinded, the outcome (e.g. muscle strength, physical performance) might have been influenced by the assessor’s knowledge of the intervention received. |  | Funding provided by: ‘Topconsortia for Knowledge and Innovation (TKIs)’ from the Ministry of Economic Affairs. One author is affiliated with FrieslandCampina. Authors declared no conflict of interest. |
| Thomson et al. 2016 (24) | + | - | x | + | + | x | Attrition rate was high (36%) and the missing outcome data might depend on its true value. Also, reasons for attrition were not balanced across the groups, which raises some concerns regarding performance bias. |  | Funding provided by: Dairy Health and Nutrition Consortium (including food companies). The sponsor assisted with diet design but had no involvement in any other aspects of the study design, study execution, data analysis or manuscript preparation. Authors declared no conflict of interest. |
| Wright et al. 2018 (8) | - | - | - | + | + | x | Unclear if allocation sequence was concealed. Also, PP analyses have been performed while 12% (n=3 of 26) were lost to follow-up because of dietary non-compliance and compliance was not reported separately for each group. Furthermore, no strong evidence provided to show that results were not biased by missing outcome data (because of dietary non-compliance). |  | Funding provided by: Egg Nutrition Center-American Egg Board and Purdue Ingestive Behavior Research Center. Funding sources had no role in study design, study execution, manuscript preparation or publication. Authors declared no conflict of interest. |
| Zhu et al. 2011 (11) | + | + | - | + | + | - | Considerable proportion of missing outcome data (11%), but no strong evidence provided to show that the result was not biased by missing outcome data. |  | Funding provided by: Australian National Health Medical Research Council and University of Western Australia Research Grants Scheme. Funding sources had no role in study design, study execution or manuscript preparation. Dietary supplements provided by: Fonterra Brands Limited. Authors declared no conflict of interest. |
| Zhu et al. 2015 (9) | + | + | - | + | + | - | Considerable proportion of missing outcome data (11%), but no strong evidence provided to show that the result was not biased by missing outcome data. |  | Funding provided by: Australian National Health Medical Research Council and University of Western Australia Research Grants Scheme. Funding sources had no role in study design, study execution or manuscript preparation. Dietary supplements provided by: Fonterra Brands Limited. Authors declared no conflict of interest. |

Abbreviations: ITT, intention to treat; LST, lean soft tissue, mITT, modified ITT; NIA, National Institute on Aging; NIH, National Institutes of Health; NR, not reported; PP, per-protocol; RCT, randomized controlled trial.

^a^ Domains addressed: 1, bias arising from the randomisation process; 2, bias due to deviations from the intended interventions; 3, bias due to missing outcome data; 4, bias in measurement of the outcome; 5, bias in selection of the reported result. Judgements include: + low risk of bias; - some concerns; x high risk of bias.(28) The RoB 2 guidance document (29) was used to ensure the correct interpretation and judgment of each domain.
^b^ The following rules were applied to reach an overall risk of bias judgement, based on:(28) the overall judgment is ‘low risk of bias’ (+) if the study is judged to be at low risk of bias for all domains; the overall judgment is ‘some concerns’ (-) if the study is judged to raise some concerns in at least one domain (but no more than two domains), but not to be at high risk of bias for any domain; the overall judgment is ‘high risk of bias’ (x) if the study is judged to be at high risk of bias in at least one domain for this result or if the study is judged to raise some concerns regarding multiple (at least three) domains.

^c^ Funding and authors’ conflict of interest are not part of the RoB 2 Cochrane collaboration tool, however these are presented here as they might be an additional concern in relation to risk of bias.

^d^ For the second domain, the committee slightly deviated from the original RoB 2 criteria. The committee did not necessarily prefer intention-to-treat analyses over per-protocol analyses (or vice versa), the choice that should be made according to the RoB 2 Cochrane collaboration tool. The committee was interested in the difference in achieved protein intake (from the background (habitual) diet plus the intervention) between the intervention- and control group at follow-up, considered as the *protein dose* in this document. This protein dose incorporates any non-compliance to the dietary intervention. In addition to the criteria described in the RoB 2 Cochrane collaboration tool, the committee assessed whether or not achieved protein intake was measured/reported and what the level of compliance was. If achieved protein intake was not available, compliance was not reported and no per-protocol analyses was performed, this would lead to some concerns regarding the risk of bias. If measured actual protein intake at follow-up was reported and the committee noted no other concerns, the risk of bias was judged as low.

Supplemental Table 11

Overview of outcome measures used for power analysis in the included studies

| Study | Outcome on which power analysis was based |
| --- | --- |
| Arnarson et al. 2013 (12), Ramel et al. 2013 (13) | Lean body mass |
| Bhasin et al. 2018 (1) | Lean body mass |
| Campbell et al. 1995 (14) | Not reported |
| Chalé et al. 2013 (15) | Lean body mass, muscle strength, physical performance |
| Dillon et al. 2009 (2) | Not reported |
| Fernandes et al. 2018 (16), Sugihara-Junior et al. 2018 (17) | Not reported |
| Ispoglou et al. 2016 (3) | Not reported |
| Kerstetter et al. 2015 (4) | Bone health (BMD) |
| Mitchell et al. 2015 (18) | Not reported |
| Mitchell et al. 2017 (5) | Lean body mass |
| Nabuco et al. 2018 (19), Nabuco et al. 2019a (20), Nabuco et al. 2019b (21) | Unclear |
| Nabuco et al. 2019c (22) | Unclear |
| Ottestad et al. 2017 (6) | Lean body mass |
| Park et al. 2018 (7) | Lean body mass |
| Ten Haaf et al. 2019 (23) | Muscle strength, physical performance |
| Thomson et al. 2016 (24) | Muscle strength |
| Wright et al. 2018 (8) | Unclear |
| Zhu et al. 2011 (11), Hodgson et al. 2012 (10), Zhu et al. 2015 (9) | Lean body mass, bone health |

Supplemental Table 12

Overview of the results of randomized controlled trials on the effect of increased protein intake on body weight in older adults, grouped according to whether or not the protein intervention was carried out in the context of (concomitant) physical exercise

| Study | Total protein intake (g/kg BW/d) during intervention^a^ | Protein type^b^ | Risk of Bias^c^ | Outcome | Result^d^ | | | | Comments |
| --- | --- | --- | --- | --- | --- | --- | --- | --- | --- |
|  |  |  |  |  | + | NS | - | ? |  |
| Not in the context of (concomitant) physical exercise | | | | | | | | | |
| Bhasin et al. 2018 (1) | IG: 1.17 ± 0.13; CG: 0.81 ± 0.10 | A,B | SC | Body weight |  | ✔ |  |  |  |
| Mitchell et al. 2017 (5) | IG: 1.7 ± 0.1; CG: 0.9 ± 0.1 | C | H | Body weight |  | ✔ |  |  |  |
| Ottestad et al. 2017 (6) | IG: 1.4 ± 0.5; CG: 0.9 ± 0.4 | B | H | Body weight |  | ✔ |  |  |  |
| Zhu et al. 2015 (9)  Same RCT as (10, 11) | IG: 1.4 ± 0.4; CG: 1.1 ± 0.4 | A | SC | Body weight |  | ✔ |  |  |  |
| *Subtotal (comparisons)* |  |  |  |  | *0* | *4* | *0* | *0* | *No effect observed for any of the 4 comparisons (4 studies)* |
| *Subtotal (studies)^e^* |  |  |  |  | *0* | *4* | *0* | *0* |  |
| In the context of (concomitant) physical exercise | | | | | | | | | |
| Chalé et al. 2013 (15) | NR | A | SC | Body weight |  | ✔ |  |  |  |
| Ten Haaf et al. 2019 (23) | IG: 0.92 ± 0.27 (without protein supplementation of 31g/d); CG: 0.97 ± 0.23 | A | SC | Body weight |  | ✔* |  |  | * P=0.07 (body weight tended to decrease more in IG than in CG. Lean body mass did not change; see Table 2) |
| Thomson et al. 2016 (24) | IG1: 1.42 ± 0.14; IG2: 1.45 ± 0.14; CG: 1.08 ± 0.05 | B | H | Body weight |  | ✔ |  |  |  |
| *Subtotal (comparisons)* |  |  |  |  | *0* | *3* | *0* | *0* | *No effect observed for any of the 3 comparisons (3 studies)* |
| *Subtotal (studies)^e^* |  |  |  |  | *0* | *3* | *0* | *0* |  |
| **Total (comparisons)** |  |  |  |  | **0** | **7** | **0** | **0** | **No effect observed for any of the 7 comparisons (7 studies)** |
| **Total (studies)^e^** |  |  |  |  | **0** | **7** | **0** | **0** |  |

Abbreviations: BW, body weight; CG, control group; H, high risk of bias; IG, intervention group; L, low risk of bias; NR, not reported; NS, not significant; SC, some concerns (regarding risk of bias).

^a^ Total protein intake during follow-up. If protein intake was assessed at multiple time points, the intake assessed at the final time point was considered.

^b^ ‘Protein type’ indicates the way in which a higher protein intake was achieved and is categorized into ‘pure’ protein or amino acids (or essential amino acids) (A), specific food(s) with a high protein content (B) or high-protein diets (C).

^c^ Risk of bias assessment: risk of bias was assessed using the RoB 2 Cochrane collaboration tool and scored as ‘low’ (L), ‘some concerns’ (SC) or ‘high’ (H).

^d^ The results of the studies are indicated as follows: +, statistically significant beneficial effect (P<0.05); -, statistically significant unfavourable effect (P<0.05); NS, no statistically significant effect (P≥0.05); ?, result unclear. In cases where results were reported for multiple time points, only the result for the final time point is reported.

^e^ Some studies assessed multiple specific outcomes (i.e. multiple contrasts) for the health outcome ‘lean body mass’, so one study can show both a significant and a non-significant effect.

Supplemental Table 13

Overview of the results of randomized controlled trials on the effect of increased protein intake in the context of concomitant physical exercise on muscle strength in older adults, categorized according to habitual protein intake and ordered by protein dose

| Study | Analytic *n* IG/CG | Total protein intake (g/kg BW/d) during intervention^a^ | Protein dose^b^ (g/kg BW/d) | Protein type^c^ | With/ without physical exercise | Risk of bias^d^ | Outcome | Result^e^ | | | |  |
| --- | --- | --- | --- | --- | --- | --- | --- | --- | --- | --- | --- | --- |
|  |  |  |  |  |  |  |  | + | NS | - | ? | Comments |
| **Habitual protein intake (reference): ≥0.8 to <0.9 kg BW/d** | | | | | | | | | | | | |
| Arnarson et al. 2013 (12)  Same RCT as (13) | 75/66 | IG: 1.06 ± 0.23; CG 0.89 ± 0.23 | 0.17 | A | Ex | H | Quadriceps strength |  | ✔ |  |  |  |
| Sugihara Junior et al. 2018 (17)  Same RCT as (16) | 15/16 | IG: 1.4 ± 0.1; CG: 0.87 ± 0.1 | 0.53 | A | Ex | H | Chest press strength | ✔ |  |  |  |  |
|  |  |  |  |  |  |  | Knee extension strength | ✔ |  |  |  |  |
|  |  |  |  |  |  |  | Preacher curl strength |  | ✔* |  |  | * P=0.07 (strength tended to increase more in IG than in CG) |
|  |  |  |  |  |  |  | Total strength^f^ | ✔ |  |  |  |  |
|  |  |  |  |  |  |  | Lower limb muscle quality index^g^ |  | ✔ |  |  |  |
|  |  |  |  |  |  |  | Upper limb muscle quality index^h^ |  | ✔ |  |  |  |
|  |  |  |  |  |  |  | Total muscle quality index^i^ |  | ✔ |  |  |  |
| *Subtotal (contrasts)* |  |  |  |  |  |  |  | *3* | *5* | *0* | *0* | *Beneficial effect observed for 3 of 8 contrasts (1 of 2 studies)* |
| *Subtotal (studies)^j^* |  |  |  |  |  |  |  | *1* | *2* | *0* | *0* |  |
| **Habitual protein intake (reference): ≥0.9 to <1.0 kg BW/d** | | | | | | | | | | | | |
| Ten Haaf et al. 2019 (23) | 58/56 for handgrip strength; 22-56^†^ (total) for other outcome measures | IG: 0.92 ± 0.27 (without protein supplementation of 31 g/d); CG: 0.97 ± 0.23 | 0.36^k^ | A | Ex | SC | Handgrip strength^§^ |  | ✔ |  |  |  |
|  |  |  |  |  |  |  | Quadriceps MVC^§^ |  | ✔ |  |  |  |
|  |  |  |  |  |  |  | Maximal rate of force rise, quadriceps^§^ |  | ✔ |  |  |  |
|  |  |  |  |  |  |  | Early relaxation time, quadriceps^§^ |  | ✔ |  |  |  |
|  |  |  |  |  |  |  | Half relaxation time, quadriceps^§^ |  | ✔ |  |  |  |
|  |  |  |  |  |  |  | Fatigue^§^ |  | ✔ |  |  |  |
| Chalé et al. 2013 (15) | 42/38 | NR | 0.38^l^ | A | Ex | SC | Double leg press strength, 1RM^§^ |  | ✔ |  |  |  |
|  |  |  |  |  |  |  | Knee extension, 1RM, right^§^ |  | ✔ |  |  |  |
|  |  |  |  |  |  |  | Knee extension, 1RM, left^§^ |  | ✔ |  |  |  |
|  |  |  |  |  |  |  | Double leg press peak power, 40% 1RM^§^ |  | ✔ |  |  |  |
|  |  |  |  |  |  |  | Knee extension peak power, 40% 1RM, right^§^ | ✔ |  |  |  |  |
|  |  |  |  |  |  |  | Knee extension peak power, 40% 1RM, left^§^ | ✔ |  |  |  |  |
|  |  |  |  |  |  |  | Double leg press peak power, 70% 1RM^§^ |  | ✔ |  |  |  |
|  |  |  |  |  |  |  | Knee extension peak power, 70% 1RM, right^§^ | ✔ |  |  |  |  |
|  |  |  |  |  |  |  | Knee extension peak power, 70% 1RM, left^§^ | ✔ |  |  |  |  |
| *Subtotal (contrasts)* |  |  |  |  |  |  |  | *4* | *11* | *0* | *0* | *Beneficial effect observed for 4 of 15 contrasts (1 of 2 studies)* |
| *Subtotal (studies)^j^* |  |  |  |  |  |  |  | *1* | *2* | *0* | *0* |  |
| **Total habitual protein intake (reference): ≥1.0 to <1.1 kg BW/d** | | | | | | | | | | | | |
| Nabuco et al. 2019c (22) | 13/13 | IG: 1.0 ± 0.23 (without ~35 g whey protein supplementation on 3 d/wk); CG: 1.0 ± 0.19 | 0.24^k^ | A | Ex | SC | Knee extension |  | ✔ |  |  |  |
|  |  |  |  |  |  |  | Chest press |  | ✔ |  |  |  |
|  |  |  |  |  |  |  | Preacher curl |  | ✔ |  |  |  |
|  |  |  |  |  |  |  | Total strength^f^ |  | ✔ |  |  |  |
| Thomson et al. 2016 (24) | 34/23 | **IG1**: 1.42 ± 0.14; CG: 1.08 ± 0.05 | 0.34 | B | Ex | H | Knee extensor strength^§^ |  | ✔ |  |  |  |
|  |  |  |  |  |  |  | Handgrip strength^§^ |  | ✔ |  |  |  |
|  |  |  |  |  |  |  | Leg press^§^ |  | ✔ |  |  |  |
|  |  |  |  |  |  |  | Chest press^§^ |  | ✔ |  |  |  |
|  |  |  |  |  |  |  | Knee extension strength^§^ |  | ✔ |  |  |  |
|  |  |  |  |  |  |  | Lat pull down^§^ |  |  |  | ✔* | * Smaller % (but not absolute) increase in IG1 than in CG |
|  |  |  |  |  |  |  | Leg curl^§^ |  | ✔ |  |  |  |
|  |  |  |  |  |  |  | Total 8RM^§^ |  | ✔ |  |  |  |
|  | 26/23 | **IG2**: 1.45 ± 0.14; CG: 1.08 ± 0.05 | 0.37 |  |  |  | Knee extensor strength^§^ |  | ✔ |  |  | * P=0.08 (strength tended to increase less in IG2 than in CG) |
|  |  |  |  |  |  |  | Handgrip strength^§^ |  | ✔ |  |  |  |
|  |  |  |  |  |  |  | Leg press^§^ |  |  | ✔ |  |  |
|  |  |  |  |  |  |  | Chest press^§^ |  | ✔ |  |  |  |
|  |  |  |  |  |  |  | Knee extension strength^§^ |  | ✔ |  |  |  |
|  |  |  |  |  |  |  | Lat pull down^§^ |  | ✔ |  |  |  |
|  |  |  |  |  |  |  | Leg curl^§^ |  | ✔ |  |  |  |
|  |  |  |  |  |  |  | Total 8RM^§^ |  |  | ✔ |  |  |
| Nabuco et al. 2018 (19)  Same RCT as (20, 21) | 22/23 | **IG1**: 1.38 ± 0.26; CG: 1.0 ± 0.25 | 0.38 | A | Ex | SC | Chest press | ✔ |  |  |  |  |
|  |  |  |  |  |  |  | Knee extension | ✔ |  |  |  |  |
|  |  |  |  |  |  |  | Preacher curl |  | ✔ |  |  |  |
|  |  |  |  |  |  |  | Total strength^f^ | ✔ |  |  |  |  |
|  | 21/23 | **IG2**: 1.49 ± 0.46; CG: 1.0 ± 0.25 | 0.49 |  |  |  | Chest press | ✔ |  |  |  |  |
|  |  |  |  |  |  |  | Knee extension | ✔ |  |  |  |  |
|  |  |  |  |  |  |  | Preacher curl |  | ✔ |  |  |  |
|  |  |  |  |  |  |  | Total strength^f^ | ✔ |  |  |  |  |
| *Subtotal (contrasts)* |  |  |  |  |  |  |  | *6* | *19* | *2* | *1* | *Beneficial effect observed for 6 of 28 contrasts (1 of 3 studies)*  *Unfavourable effect observed for 2 of 28 contrasts (1 of 3 studies)* |
| *Subtotal (studies)^j^* |  |  |  |  |  |  |  | *1* | *3* | *1* | *1* |  |
| **Total habitual protein intake (reference): Unclear** | | | | | | | | | | | | |
| Mitchell et al. 2015 (18) | 16 (total) | NR | NR (15 g/d) | B | Ex | H | Knee extension isometric MVC |  | ✔ |  |  |  |
|  |  |  |  |  |  |  | Leg press |  | ✔ |  |  |  |
|  |  |  |  |  |  |  | Leg extension |  | ✔ |  |  |  |
|  |  |  |  |  |  |  | Chest press |  | ✔ |  |  |  |
| *Subtotal (contrasts)* |  |  |  |  |  |  |  | *0* | *4* | *0* | *0* | *No effect observed for any of 4 contrasts (1 study)* |
| *Subtotal (studies)^j^* |  |  |  |  |  |  |  | *0* | *1* | *0* | *0* |  |
| **Total (contrasts)** |  |  |  |  |  |  |  | **13** | **39** | **2** | **1** | **Beneficial effect observed for 13 of 55 contrasts (3 of 8 studies)**  **Unfavourable effect observed for 2 of 55 contrasts (1 of 15 studies)** |
| **Total (studies)^j^** |  |  |  |  |  |  |  | **3** | **8** | **1** | **1** |  |

Abbreviations: BW, body weight; CG, control group; Ex, with concomitant exercise intervention; H, high risk of bias; IG, intervention group; L, low risk of bias; MVC, maximal voluntary contraction; NoEx, without concomitant exercise intervention; NR, not reported; RM, repetition maximum; SC, some concerns (regarding risk of bias).

^†^ Depending on specific outcome measure.

^§^ Sufficient statistical power to detect an effect is to be expected, based on the sample size calculation.

^a^ Total protein intake during follow-up. If protein intake was assessed at multiple time points, the intake assessed at the final time point was considered.

^b^ ‘Protein type’ indicates the way in which a higher protein intake was achieved and is categorized into ‘pure’ protein or amino acids (or essential amino acids) (A), specific product with a high protein content (B) or high-protein diets (C).

^c^ ‘Protein dose’ indicates the difference in achieved total protein intake between the intervention and control groups during follow-up (which is not necessarily equal to supplemented/prescribed amount of protein).

^d^ Risk of bias assessment: risk of bias was assessed using the RoB 2 Cochrane collaboration tool and scored as ‘low’ (L), ‘some concerns’ (SC) or ‘high’ (H).

^e^ The results of the studies are indicated as follows: +, statistically significant beneficial effect (P<0.05); -, statistically significant unfavourable effect (P<0.05); NS, no statistically significant effect (P≥0.05); ?, result unclear. In cases where results were reported for multiple time points, only the result for the final time point is reported.

^f^ Total strength was calculated as the sum of chest press, knee extension and preacher curl strength (kg).

^g^ Lower limb muscle quality index was calculated as knee extension strength divided by lower limb lean soft tissue.

^h^ Upper limb muscle quality index was calculated as preacher curl strength divided by upper limb lean soft tissue.

^i^ Total muscle quality index was calculated as total strength divided by skeletal muscle mass.

^j^ Some studies assessed multiple specific outcomes (i.e. multiple contrasts) for the health outcome ‘muscle strength’, so one study can show both a significant and a non-significant effect.

^k^ Protein intake in g/kg BW/d was calculated by using protein intake in g/d, mean body weight (and compliance, if available).

^l^ (Achieved) protein dose was estimated using prescribed protein dose, compliance rate (72%), and mean body weight.

Supplemental Results 1

Subgroup analyses according to the presence of (concomitant) physical exercise or not

The committee evaluated the effect of increased protein intake on each health outcome according to whether or not the protein intervention took place in the context of a (concomitant) physical exercise intervention. The results are describe below for the outcomes of lean body mass, muscle strength, physical performance and serum lipids. For the other outcomes no subdivision could be made because too few studies were available, or a subdivision was not made (considered unnecessary) as (nearly) all studies for a given health outcome showed the same result (no effect).

**Lean body mass**

Four of the 9 studies (44%; 10 of 35 contrasts (29%)) on the effect of increased protein intake alone showed a beneficial effect on lean body mass, as did 3 of the 9 studies (33%; 11 of 27 contrasts (41%)) on the effect of increased protein intake in the context of physical exercise.

**Muscle strength**

One of 7 studies (14%; 1 of 28 contrasts (4%)) on the effect of increased protein intake alone showed a beneficial effect on muscle strength, as did 3 of the 8 studies (38%; 13 of 55 contrasts (24%)) on the effect of increased protein intake in the context of physical exercise. The committee did subdivide the 8 RCTs in which protein intake is combined with physical exercise according to the domain of habitual protein intake in Supplemental Table 13 and Supplemental Results 1.

**Physical performance**

One of the 6 studies (17%; 1 of 25 contrasts (4%)) on the effect of increased protein intake alone showed a beneficial effect on physical performance, which was similar to the number of studies on the effect of increased protein in the context of physical exercise (1 of 6 studies (17%); 2 of 19 contrasts (11%)).

**Serum lipids**

One of the 4 studies (25%; 1 of 21 contrasts (5%)) on the effect of increased protein intake alone showed a beneficial effect on serum lipids, as did 1 of the 3 studies (33%; 1 of 22 contrasts (5%)) on the effect of increased protein intake in the context of physical exercise.

An unfavourable effect on serum lipids was found in one of the 4 studies (25%; 1 of 21 contrasts (5%)) on the effect of increased protein intake alone, but not in any of the 3 studies on the effect of increased protein intake in the context of physical exercise.

Supplemental Results 2

Subgroup analyses according to domain of habitual protein intake

The committee evaluated the effect of increased protein intake on each health outcome according to domain of habitual protein intake. The results are describe below for the outcomes of lean body mass, muscle strength, physical performance and serum lipids. For the other outcomes no subdivision could be made because too few studies were available, or a subdivision was not made (considered unnecessary) as (nearly) all studies for a given health outcome showed the same result (no effect).

**Lean body mass**

Habitual protein intake (reference): ≥0.8 to <0.9 g/kg BW/d

Against a background (habitual) intake of 0.8 to 0.9 g protein/kg BW/d, the committee evaluated five RCTs on the effect of increased protein intake on lean body mass, with a total of 18 statistically tested contrasts. Those studies involved a total of approximately 290 participants. The protein dose ranged from 0.17 to 0.82 g/kg BW/d. The risk of bias in those studies was scored as ‘some concerns’ (n=2) or ‘high’ (n=3).

In 2 of the 5 studies (40%), a beneficial effect of increased protein intake was found for at least one of the statistically tested contrasts (4 of 18 contrasts, 22%). In those studies that showed an effect, the protein dose ranged from 0.53 to 0.6 g/kg BW/d. Increased protein intake, within this domain of habitual protein intake, was not found to have any unfavourable effects on lean body mass.

Habitual protein intake (reference): ≥0.9 to <1.0 g/kg BW/d

Against a background intake of 0.9 to 1.0 g protein/kg BW/d, the committee evaluated five RCTs on the effect of increased protein intake on lean body mass, with a total of 18 statistically tested contrasts. Those studies involved a total of approximately 380 participant. The protein dose ranged from 0.28 to 0.8 g/kg BW/d. The risk of bias in those studies was scored as ‘some concerns’ (n=3) or ‘high’ (n=2).

In 2 of the 5 studies (40%), a beneficial effect of increased protein intake was found for at least one of the statistically tested contrasts (7 of 18 contrasts, 39%). In those studies that showed an effect, the protein dose ranged from 0.47 to 0.8 g/kg BW/d. Increased protein intake, within this domain of habitual protein intake, was not found to have any unfavourable effects on lean body mass.

Habitual protein intake (reference): ≥1.0 to <1.1 g/kg BW/d

Against a background intake of 1.0 to 1.1 g protein/kg BW/d, the committee evaluated five RCTs on the effect of increased protein intake on lean body mass, with a total of 19 statistically tested contrasts. Those studies involved a total of approximately 410 participants. The protein dose ranged from 0.21 to 0.49 g/kg BW/d. The risk of bias in those studies was scored as ‘some concerns’ (n=3) or ‘high’ (n=2).

In 3 of the 5 studies (60%), a beneficial effect of increased protein intake was found for at least one of the statistically tested contrasts (10 of 19 contrasts, 53%). In those studies that showed an effect, the protein dose ranged from 0.24 to 0.49 g/kg BW/d. Increased protein intake, within this domain of habitual protein intake, was not found to have any unfavourable effects on lean body mass.

Habitual protein intake (reference): ≥1.1 g/kg BW/d

Against a background intake of at least 1.1 g protein/kg BW/d, the committee evaluated one RCT on the effect of increased protein intake on lean body mass, with a total of five statistically tested contrasts. This study involved a total of 181 participants. The protein dose was 0.3 g/kg BW/d. This study, with some concerns regarding the risk of bias, found no effects of increased protein intake on lean body mass.

In two RCTs, the habitual protein intake was unclear. The committee has included those studies in the evaluation of the overall effect of protein intake, but not in the subdivision by domain of habitual protein intake.

**Muscle strength**

Because increased protein intake alone (not in the context of physical exercise) has likely no effect on muscle strength in older adults, the committee did not further subdivide those studies according to the domain of habitual protein intake. Those studies in which protein intake is combined with physical exercise (n=8) are further evaluated based on subgroups of habitual protein intake the following paragraphs (see **Supplemental Table 13** for the summary table for those eight RCTs only).

Habitual protein intake (reference): ≥0.8 to <0.9 g/kg BW/d

Against a background (habitual) intake of 0.8 to 0.9 g protein/kg BW/d, the committee evaluated two RCTs on the effect of increased protein intake (in the context of physical exercise) on muscle strength, with a total of eight statistically tested contrasts. Those studies involved a total of 172 participants. The protein dose ranged from 0.17 to 0.53 g/kg BW/d. The risk of bias in both studies was scored as ‘high’.

In 1 of the 2 studies (50%), a beneficial effect of increased protein intake was found for at least one of the statistically tested contrasts (3 of 8 contrasts, 38%). In those studies that showed an effect, the protein dose was 0.53 g/kg BW/d. Increased protein intake, within this domain of habitual protein intake, was not found to have any unfavourable effects on muscle strength.

Habitual protein intake (reference): ≥0.9 to <1.0 g/kg BW/d

Against a background intake of 0.9 to 1.0 g protein/kg BW/d, the committee evaluated two RCTs on the effect of increased protein intake (in the context of physical exercise) on muscle strength, with a total of 15 statistically tested contrasts. Those studies involved a total of 175 participants. The risk of bias in both studies was scored as ‘some concerns’.

The protein dose ranged from 0.36 to 0.38 g/kg BW/d. In 1 of the 2 studies (50%), a beneficial effect of increased protein intake was found for at least one of the statistically tested contrasts (4 of 15 contrasts, 27%). In this study that showed an effect, the protein dose was 0.38 g/kg BW/d. Increased protein intake, within this domain of habitual protein intake, was not found to have any unfavourable effects on muscle strength.

Habitual protein intake (reference): ≥1.0 to <1.1 g/kg BW/d

Against a background intake of 1.0 to 1.1 g protein/kg BW/d, the committee evaluated three RCTs on the effect of increased protein intake (in in the context of physical exercise) on muscle strength, with a total of 28 statistically tested contrasts. Those studies involved a total of 194 participants. The protein dose ranged from 0.24 to 0.49 g/kg BW/d. The risk of bias in those studies was scored as ‘some concerns’ (n=2) or ‘high’ (n=1).

In 1 of the 3 studies (33%), a beneficial effect of increased protein intake was found for at least one of the statistically tested contrasts (6 of 28 contrasts, 21%). In this study that showed a beneficial effect, the protein dose ranged from 0.38 to 0.49 g/kg BW/d and the risk of bias was scored as ‘some concerns’. One study (33%) found an unfavourable effect on muscle strength (2 of 28 contrasts, 7%). In this study that showed an unfavourable effect, the protein dose was 0.37 g/kg BW/d and the risk of bias was scored as ‘high’.

Habitual protein intake (reference): ≥1.1 g/kg BW/d

The committee found no RCTs within this domain of habitual protein intake.

In one RCT, the habitual protein intake was unclear. The committee has included this study in the evaluation of the overall effect of protein intake, but not in the subdivision by domain of habitual protein intake.

**Physical performance**

Habitual protein intake (reference): ≥0.8 to <0.9 g/kg BW/d

Against a background (habitual) intake of 0.8 to 0.9 g protein/kg BW/d, the committee evaluated two RCTs on the effect of increased protein intake on physical performance, with a total of seven statistically tested contrasts. Those studies involved a total of approximately 210 participants. The protein dose ranged from 0.17 to 0.36 g/kg BW/d. The risk of bias in those studies was scored as ‘some concerns’ (n=1) or ‘high’ (n=1). Those studies showed no effects of increased protein intake on physical performance.

Habitual protein intake (reference): ≥0.9 to <1.0 g/kg BW/d

Against a background intake of 0.9 to 1.0 g protein/kg BW/d, the committee evaluated five RCTs on the effect of increased protein intake on physical performance, with a total of 24 statistically tested contrasts. Those studies involved a total of approximately 370 participants. The protein dose ranged from 0.25 to 0.8 g/kg BW/d. The risk of bias in those studies was scored as ‘some concerns’ (n=3) or ‘high’ (n=2).

In 1 of the 5 studies (20%), a beneficial effect of increased protein intake was found for at least one of the statistically tested contrasts (1 of 24 contrasts, 4%). In this study that showed an effect, the protein dose was 0.47 g/kg BW/d. Increased protein intake, within this domain of habitual protein intake, was not found to have any unfavourable effects on physical performance.

Habitual protein intake (reference): ≥1.0 to <1.1 g/kg BW/d

Against a background intake of 1.0 to 1.1 g protein/kg BW/d, the committee evaluated four RCTs on the effect of increased protein intake on physical performance, with a total of 12 statistically tested contrasts. Those studies involved a total of approximately 200 participants. The protein dose ranged from 0.21 to 0.49 g/kg BW/d. The risk of bias in those studies was scored as ‘some concerns’ (n=2) or ‘high’ (n=2).

In 1 of the 4 studies (25%), a beneficial effect of increased protein intake was found for at least one of the statistically tested contrasts (2 of 12 contrasts, 17%). In this study that showed an effect, the protein dose ranged from 0.38 to 0.49 g/kg BW/d. Increased protein intake, within this domain of habitual protein intake, was not found to have any unfavourable effects on physical performance.

Habitual protein intake (reference): **≥1.1** g/kg BW/d

Against a background intake of at least 1.1 g protein/kg BW/d, the committee evaluated one RCT on the effect of increased protein intake on physical performance, with a total of one statistically tested contrast. This study involved a total of 181 participants. The protein dose was 0.3 g/kg BW/d. The risk of bias in this study was scored as ‘some concerns’. This study showed no effect of increased protein intake on physical performance.

**Serum lipids**

Habitual protein intake (reference): ≥0.8 to <0.9 g/kg BW/d

Against a background (habitual) intake of 0.8 to 0.9 g protein/kg BW/d, the committee evaluated three RCTs on the effect of increased protein intake on serum lipids, with a total of 15 statistically tested contrasts. These studies involved a total of approximately 140 participants. The protein dose ranged from 0.36 to 0.6 g/kg BW/d. The risk of bias in those studies was scored as ‘some concerns’ (n=1) or ‘high’ (n=2).

In 1 of the 3 studies (33%), a beneficial effect of increased protein intake was found for at least one of the statistically tested contrasts (1 of 15 contrasts, 7%). This concerned the total-to-HDL cholesterol ratio. In the study that showed this beneficial effect, the protein dose was 0.53 g/kg BW/d. One other study found an unfavourable effect of increased protein intake on serum lipids (1 of 15 contrasts, 7%), specifically concerning LDL cholesterol. This study used a protein dose of 0.6 g/kg BW/d.

Habitual protein intake (reference): ≥0.9 to <1.0 g/kg BW/d

Against a background intake of 0.9 to 1.0 g protein/kg BW/d, the committee evaluated two RCTs on the effect of increased protein intake on serum lipids, with a total of 12 statistically tested contrasts. Those studies involved a total of approximately 150 participants. The protein dose ranged from 0.28 to 0.5 g/kg BW/d. The risk of bias in those studies was scored as ‘some concerns’ (n=1) or ‘high’ (n=1).

In 1 of the 2 studies (50%), a beneficial effect was found for at least one of the statistically tested contrasts (1 of 12 contrasts, 8%), specifically concerning triglycerides. In the study that showed this beneficial effect, the protein dose was 0.5 g/kg BW/d. Increased protein intake, within this domain of habitual protein intake, was not found to have any unfavourable effects on serum lipids.

Habitual protein intake (reference): ≥1.0 to <1.1 g/kg BW/d

Against a background intake of 1.0 to 1.1 g protein/kg BW/d, the committee evaluated two RCTs on the effect of increased protein intake on serum lipids, with a total of 16 statistically tested contrasts. These studies involved a total of approximately 90 participants. The protein dose ranged from 0.24 to 0.49 g/kg BW/d. The risk of bias in those studies was scored as ‘some concerns’. Those studies showed no effects of increased protein intake on serum lipids.

References

1. Bhasin S, Apovian CM, Travison TG, Pencina K, Moore LL, Huang G, et al. Effect of Protein Intake on Lean Body Mass in Functionally Limited Older Men: A Randomized Clinical Trial. JAMA internal medicine. 2018;178(4):530-41.

2. Dillon EL, Sheffield-Moore M, Paddon-Jones D, Gilkison C, Sanford AP, Casperson SL, et al. Amino acid supplementation increases lean body mass, basal muscle protein synthesis, and insulin-like growth factor-I expression in older women. J Clin Endocrinol Metab. 2009;94(5):1630-7.

3. Ispoglou T, White H, Preston T, McElhone S, McKenna J, Hind K. Double-blind, placebo-controlled pilot trial of L-Leucine-enriched amino-acid mixtures on body composition and physical performance in men and women aged 65-75 years. European journal of clinical nutrition. 2016;70(2):182-8.

4. Kerstetter JE, Bihuniak JD, Brindisi J, Sullivan RR, Mangano KM, Larocque S, et al. The Effect of a Whey Protein Supplement on Bone Mass in Older Caucasian Adults. J Clin Endocrinol Metab. 2015;100(6):2214-22.

5. Mitchell CJ, Milan AM, Mitchell SM, Zeng N, Ramzan F, Sharma P, et al. The effects of dietary protein intake on appendicular lean mass and muscle function in elderly men: a 10-wk randomized controlled trial. Am J Clin Nutr. 2017;106(6):1375-83.

6. Ottestad I, Lovstad AT, Gjevestad GO, Hamarsland H, Saltyte Benth J, Andersen LF, et al. Intake of a Protein-Enriched Milk and Effects on Muscle Mass and Strength. A 12-Week Randomized Placebo Controlled Trial among Community-Dwelling Older Adults. The journal of nutrition, health & aging. 2017;21(10):1160-9.

7. Park Y, Choi JE, Hwang HS. Protein supplementation improves muscle mass and physical performance in undernourished prefrail and frail elderly subjects: a randomized, double-blind, placebo-controlled trial. Am J Clin Nutr. 2018;108(5):1026-33.

8. Wright CS, Zhou J, Sayer RD, Kim JE, Campbell WW. Effects of a High-Protein Diet Including Whole Eggs on Muscle Composition and Indices of Cardiometabolic Health and Systemic Inflammation in Older Adults with Overweight or Obesity: A Randomized Controlled Trial. Nutrients. 2018;10(7).

9. Zhu K, Kerr DA, Meng X, Devine A, Solah V, Binns CW, et al. Two-Year Whey Protein Supplementation Did Not Enhance Muscle Mass and Physical Function in Well-Nourished Healthy Older Postmenopausal Women. The Journal of nutrition. 2015;145(11):2520-6.

10. Hodgson JM, Zhu K, Lewis JR, Kerr D, Meng X, Solah V, et al. Long-term effects of a protein-enriched diet on blood pressure in older women. The British journal of nutrition. 2012;107(11):1664-72.

11. Zhu K, Meng X, Kerr DA, Devine A, Solah V, Binns CW, et al. The effects of a two-year randomized, controlled trial of whey protein supplementation on bone structure, IGF-1, and urinary calcium excretion in older postmenopausal women. Journal of bone and mineral research : the official journal of the American Society for Bone and Mineral Research. 2011;26(9):2298-306.

12. Arnarson A, Gudny Geirsdottir O, Ramel A, Briem K, Jonsson PV, Thorsdottir I. Effects of whey proteins and carbohydrates on the efficacy of resistance training in elderly people: double blind, randomised controlled trial. European journal of clinical nutrition. 2013;67(8):821-6.

13. Ramel A, Arnarson A, Geirsdottir OG, Jonsson PV, Thorsdottir I. Glomerular filtration rate after a 12-wk resistance exercise program with post-exercise protein ingestion in community dwelling elderly. Nutrition (Burbank, Los Angeles County, Calif). 2013;29(5):719-23.

14. Campbell WW, Crim MC, Young VR, Joseph LJ, Evans WJ. Effects of resistance training and dietary protein intake on protein metabolism in older adults. Am J Physiol. 1995;268(6 Pt 1):E1143-53.

15. Chale A, Cloutier GJ, Hau C, Phillips EM, Dallal GE, Fielding RA. Efficacy of whey protein supplementation on resistance exercise-induced changes in lean mass, muscle strength, and physical function in mobility-limited older adults. The journals of gerontology Series A, Biological sciences and medical sciences. 2013;68(6):682-90.

16. Fernandes RR, Nabuco HCG, Sugihara Junior P, Cavalcante EF, Fabro PMC, Tomeleri CM, et al. Effect of protein intake beyond habitual intakes following resistance training on cardiometabolic risk disease parameters in pre-conditioned older women. Experimental gerontology. 2018;110:9-14.

17. Sugihara Junior P, Ribeiro AS, Nabuco HCG, Fernandes RR, Tomeleri CM, Cunha PM, et al. Effects of Whey Protein Supplementation Associated With Resistance Training on Muscular Strength, Hypertrophy, and Muscle Quality in Preconditioned Older Women. International journal of sport nutrition and exercise metabolism. 2018;28(5):528-35.

18. Mitchell CJ, Oikawa SY, Ogborn DI, Nates NJ, MacNeil LG, Tarnopolsky M, et al. Daily chocolate milk consumption does not enhance the effect of resistance training in young and old men: a randomized controlled trial. Appl Physiol Nutr Metab. 2015;40(2):199-202.

19. Nabuco HCG, Tomeleri CM, Sugihara Junior P, Fernandes RR, Cavalcante EF, Antunes M, et al. Effects of Whey Protein Supplementation Pre- or Post-Resistance Training on Muscle Mass, Muscular Strength, and Functional Capacity in Pre-Conditioned Older Women: A Randomized Clinical Trial. Nutrients. 2018;10(5).

20. Nabuco HCG, Tomeleri CM, Sugihara Junior P, Fernandes RR, Cavalcante EF, Venturini D, et al. Effects of pre- or post-exercise whey protein supplementation on body fat and metabolic and inflammatory profile in pre-conditioned older women: A randomized, double-blind, placebo-controlled trial. Nutrition, metabolism, and cardiovascular diseases : NMCD. 2019;29(3):290-300.

21. Nabuco HCG, Tomeleri CM, Sugihara PJ, Fernandes RR, Cavalcante EF, Dos Santos L, et al. Effect of whey protein supplementation combined with resistance training on cellular health in pre-conditioned older women: A randomized, double-blind, placebo-controlled trial. Archives of gerontology and geriatrics. 2019;82:232-7.

22. Nabuco HCG, Tomeleri CM, Fernandes RR, Sugihara Junior P, Cavalcante EF, Cunha PM, et al. Effect of whey protein supplementation combined with resistance training on body composition, muscular strength, functional capacity, and plasma-metabolism biomarkers in older women with sarcopenic obesity: A randomized, double-blind, placebo-controlled trial. Clin Nutr ESPEN. 2019;32:88-95.

23. Ten Haaf DSM, Eijsvogels TMH, Bongers C, Horstman AMH, Timmers S, de Groot L, et al. Protein supplementation improves lean body mass in physically active older adults: a randomized placebo-controlled trial. J Cachexia Sarcopenia Muscle. 2019;10(2):298-310.

24. Thomson RL, Brinkworth GD, Noakes M, Buckley JD. Muscle strength gains during resistance exercise training are attenuated with soy compared with dairy or usual protein intake in older adults: A randomized controlled trial. Clinical nutrition (Edinburgh, Scotland). 2016;35(1):27-33.

25. Siri WE. Body composition from fluid spaces and density: analysis of methods. 1961. Nutrition (Burbank, Los Angeles County, Calif). 1993;9(5):480-91; discussion , 92.

26. Kim J, Heshka S, Gallagher D, Kotler DP, Mayer L, Albu J, et al. Intermuscular adipose tissue-free skeletal muscle mass: estimation by dual-energy X-ray absorptiometry in adults. J Appl Physiol (1985). 2004;97(2):655-60.

27. Kim J, Wang Z, Heymsfield SB, Baumgartner RN, Gallagher D. Total-body skeletal muscle mass: estimation by a new dual-energy X-ray absorptiometry method. Am J Clin Nutr. 2002;76(2):378-83.

28. Sterne JAC, Savovic J, Page MJ, Elbers RG, Blencowe NS, Boutron I, et al. RoB 2: a revised tool for assessing risk of bias in randomised trials. BMJ. 2019;366:l4898.

29. Higgins JPT, Savovic J, Page MJ, Sterne JAC. Revised Cochrane risk-of-bias tool for randomized trials (RoB 2). Guidance document for individually-randomized, parallel-group trials. 2019.

1. The branched-chain amino acids are three essential amino acids: leucine, isoleucine and valine. [↑](#footnote-ref-1)
2. The committee distinguished between a ‘hard’ and a ‘soft’ contrast. An example of a hard contrast is when the intervention group is given a protein supplement as well as a vitamin D supplement, whereas the control group is given neither. The researchers’ *intention* was to investigate the effect of the combination of protein and vitamin D. An example of a soft contrast is when the intervention group is given a food-based protein supplement (e.g. milk) to investigate the effect of higher protein intake, as this also causes the group to consume (albeit unintentionally) other nutrients that are present in the supplement/provided food. [↑](#footnote-ref-2)
